# Exploring Gallium(III) Complexes as Emerging Therapeutic Candidates for Breast Cancer

**DOI:** 10.1021/acs.jmedchem.5c03480

**Published:** 2026-04-06

**Authors:** Alberto Moreno-Fernández, Elena Domínguez-Jurado, Marc Martínez de Sarasa Buchaca, Sofía Blas, María del Mar Noblejas-López, Carmen Moya, Fernando de Andrés, Iván Bravo, Jose-Daniel Aroca-Aguilar, Jesús-José Ferre-Fernández, Julio Escribano, Antonio Rodríguez-Diéguez, Pablo Salcedo-Abraira, Santiago García-Yuste, Carlos Alonso-Moreno, Agustín Lara-Sánchez

**Affiliations:** † Universidad de Castilla-La Mancha, Departamento de Química Inorgánica, Orgánica y Bioquímica-Centro de Innovación en Química Avanzada (ORFEO-CINQA), Facultad de Ciencias y Tecnologías Químicas, Instituto Regional de Investigación Científica Aplicada-IRICA, 13071 Ciudad Real, Spain; ‡ Universidad de Castilla-La Mancha, Departamento de Química Inorgánica, Orgánica y Bioquímica-Centro de Innovación en Química Avanzada (ORFEO-CINQA), Unidad NanoDrug, Facultad de Farmacia, 02071 Albacete, Spain; § Universidad de Castilla-La Mancha, Departamento de Química Analítica y Tecnología de Alimentos, Facultad de Farmacia de Albacete, 02071 Albacete, Spain; ∥ Universidad de Castilla-La Mancha, Instituto Regional de Investigación Científica Aplicada IRICA, 13005 Ciudad Real, Spain; ⊥ Universidad de Castilla-La Mancha, Genetics, Castilla-La Mancha University Medical School, 02008 Albacete, Spain; # Universidad de Granada, Departamento de Química Inorgánica, Facultad de Ciencias, 18071 Granada, Spain

## Abstract

Despite the clinical success of platinum chemotherapeutics, severe side effects and resistance drive the search for alternative metallodrugs. Gallium compounds are promising due to their ability to mimic iron­(III) and disrupt essential cellular processes; however, poor aqueous stability and moderate cytotoxicity have limited their development. Herein, we report a new family of heteroscorpionate gallium­(III) salts, [Ga­(κ^3^-NNO)_2_]­[GaCl_4_] (**Ga1–Ga6**). NMR spectroscopy and single-crystal X-ray diffraction confirmed their molecular structures. The complexes exhibit remarkable air and moisture stability, with tunable lipophilicity and solubility governed by ligand electronics. **Ga3** and **Ga6**, bearing dimethylamino substituents, showed nanomolar cytotoxicity across breast cancer cell lines, outperforming classical platinum drugs. The lead compound **Ga6** displayed high hydrolytic stability, selective tumor-cell uptake, cytoplasmic localization, and significant tumor growth inhibition in zebrafish xenografts without observable systemic toxicity in mice, underscoring the potential of the heteroscorpionate platform.

## Graphical abstract

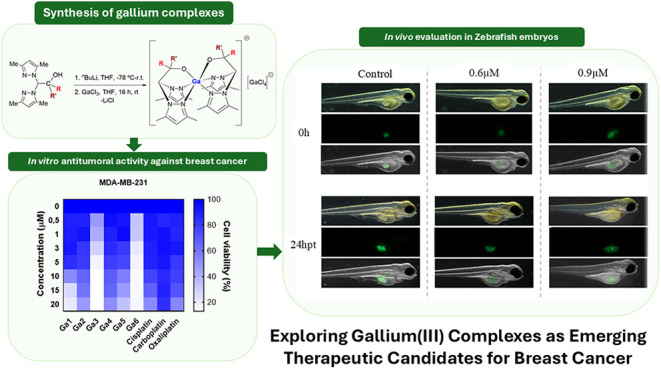

## Introduction

Breast cancer remains a leading cause of cancer-related mortality worldwide,^1^ and continues to require more effective and better-tolerated therapeutic strategies.^2–4^ Although platinum-based agents such as cisplatin, carboplatin, and oxaliplatin are widely used in the treatment of advanced and metastatic disease,^5,6^ their clinical utility is limited by severe systemic toxicity and the frequent development of resistance.^7,8^ These drawbacks have motivated the search for alternative metal-based chemotherapeutics capable of retaining potent antitumor activity while offering improved selectivity and pharmacological profiles. In this context, bioinorganic and organometallic chemistry provide powerful platforms for the rational design of structurally tunable metallodrugs.

Among nonplatinum metallodrugs,^3,9–12^ gallium­(III) compounds have attracted increasing attention due to their biochemical mimicry of iron­(III) and their ability to disrupt iron-dependent cellular processes.^13–16^ Gallium interferes with iron uptake, transport, and storage pathways, ultimately impairing DNA synthesis and mitochondrial function in rapidly proliferating cancer cells. These properties position Ga­(III) as a promising alternative metal center in oncology. Gallium isotopes such as ^67^Ga and ^68^Ga further highlight its biomedical relevance.^17–19^ However, the clinical performance of Ga-based compounds remains strongly dependent on ligand design, which governs hydrolytic stability, solubility, and cellular uptake, key parameters that continue to represent major challenges in the development of effective gallium metallodrugs.

The therapeutic relevance of gallium was first demonstrated with gallium­(III) nitrate, the earliest Ga-based compound evaluated clinically as an antineoplastic agent.^20–22^ Its activity is primarily attributed to disruption of cellular iron homeostasis through biochemical mimicry of Fe­(III).^20,23^ Subsequent development of more stable and bioavailable derivatives led to gallium maltolate (GaM)^24,25^ and tris­(8-quinolinolato)­gallium­(III) (KP46),^26,27^ the most advanced Ga-based clinical candidates. These orally administrable complexes exhibit improved hydrolytic stability and encouraging antitumor profiles,^28^ further validating gallium as a viable metal center in oncology.^29^

Despite this progress, the biological performance of Ga­(III) complexes remains highly dependent on ligand architecture. The coordination sphere governs hydrolytic stability, solubility, redox behavior, and cellular uptake,^20–22^ making ligand design central to the rational development of gallium metallodrugs.^30^ A broad range of ligand systems, including O- and N-donor chelates, macrocycles, porphyrins, and dithiocarbamates, has been explored to optimize Ga­(III) coordination.^20,24–27,31–35^ However, structurally robust Ga­(III) complexes that maintain long-term integrity in aqueous media while displaying potent antiproliferative activity remain comparatively rare. This unmet need highlights the importance of developing new coordination platforms capable of providing both structural control and tunable biological performance.

Scorpionate and heteroscorpionate ligands offer a rigid κ^3^ coordination platform capable of enforcing well-defined metal geometries while allowing systematic electronic and steric modulation.^36–43^ Although widely applied in coordination chemistry, their use in medicinal inorganic chemistry remains comparatively limited.^44–46^ The ability of these tridentate N,O-donor frameworks to generate structurally robust and electronically tunable coordination spheres suggests a promising strategy to address the stability and ligand-control challenges associated with Ga­(III) complexes. Building on our previous work demonstrating the anticancer potential of scorpionate-supported ruthenium complexes,^3,46^ we sought to translate this design concept to gallium­(III).

Herein, we report the synthesis, structural characterization, and biological evaluation of a series of alkoxide-functionalized heteroscorpionate Ga­(III) complexes (**Ga1–Ga6**). Systematic variation of ligand substituents enabled modulation of lipophilicity, solubility, and aqueous stability, directly influencing cytotoxic activity across multiple breast cancer subtypes. Among the series, **Ga3** and **Ga6** displayed nanomolar potency and favorable selectivity profiles. In vivo evaluation in zebrafish xenografts, together with preliminary murine tolerability studies, further supported the therapeutic potential of this scaffold. Collectively, these findings establish heteroscorpionate coordination as a structurally defined and tunable platform for the development of next-generation gallium-based metallodrugs for breast cancer.

## Results and Discussion

### Synthesis and Structural Characterization of the Novel Gallium-Based Metallodrugs

The new family of heteroscorpionate gallium­(III) complexes [Ga­(κ^3^-NNO)_2_]^+^[GaCl_4_]^−^ (**Ga1–Ga6**) was successfully synthesized through a straightforward two-step metathesis reaction from the corresponding lithium-heteroscorpionate precursors and GaCl_3_ in THF at room temperature (Scheme 1).^47^ The modular design of the ligands (**L1–L6**): 1,1-bis­(3,5-dimethyl-1*H*-pyrazol-1-yl)-3,3-dimethylbutan-2-ol (bpzbeH; **L1**), (2,2-bis­(3,5-dimethyl-1*H*-pyrazol-1-yl)-1-ferrocenyl)­ethan-1-ol (bpzFerrH; **L2**), 2,2-bis­(3,5-dimethyl-1*H*-pyrazol-1-yl)-1-(4-(dimethylamino)­phenyl)-1-phenylethan-1-ol (bpzappeH; **L3**), 2,2-bis­(3,5-dimethyl-1*H*-pyrazol-1-yl)-1-(naphthalen-1-yl)­ethan-1-ol (bpznapheH; **L4**), 1-(anthracen-9-yl)-2,2-bis­(3,5-dimethyl-1*H*-pyrazol-1-yl)­ethan-1-ol (bpzantheH; **L5**) and 2,2-bis­(3,5-dimethyl-1*H*-pyrazol-1-yl)-1,1-bis­(4-(dimethylamino)­phenyl)­ethan-1-ol, (bpzbdmapeH; **L6**) allowed systematic tuning of their steric and electronic environments to evaluate how subtle modifications influence the physicochemical and biological behavior of the resulting complexes. This rational approach not only enabled the incorporation of diverse aromatic and donor substituents, including ferrocenyl, naphthyl, anthracenyl, and dimethylamino groups, but also provided a direct structure–activity correlation relevant to antitumor performance. Remarkably, all compounds were isolated in high yields and exhibited exceptional stability under air and moisture, a rare feature among Ga­(III) organometallic species and a key prerequisite for their subsequent biological testing.

**Scheme 1:**
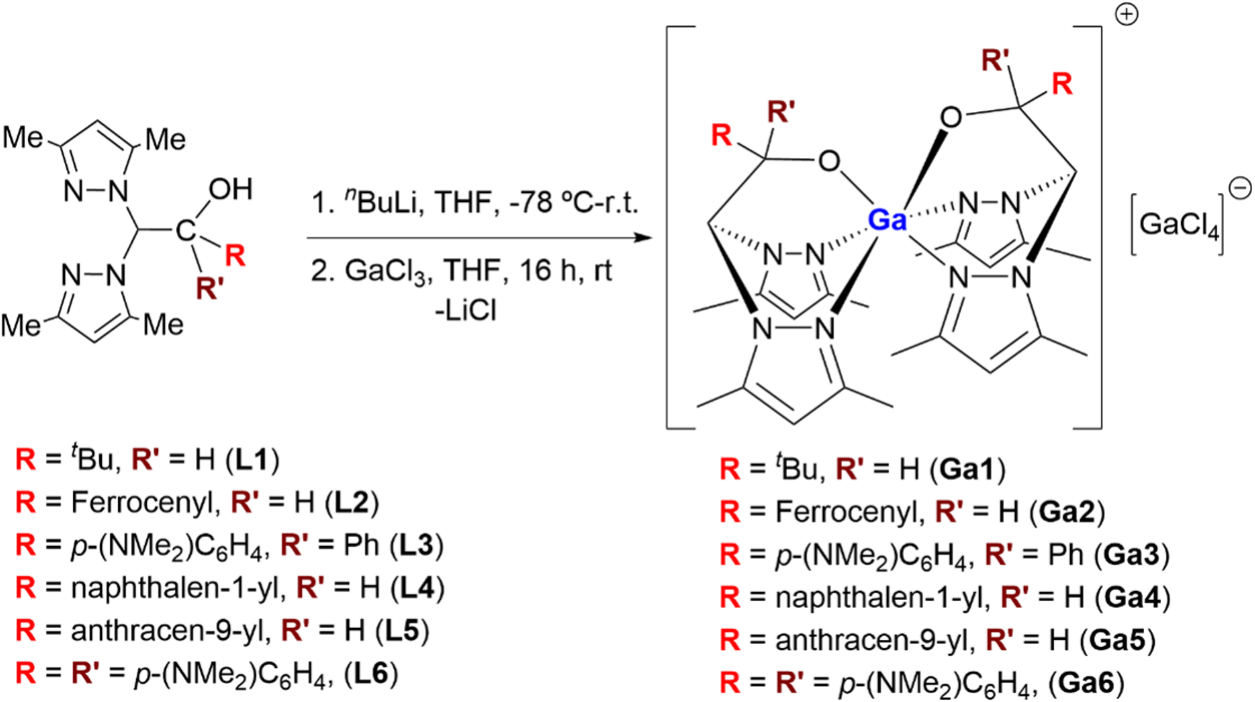
Synthetic Route for the Heteroscorpionate Gallium­(III) Complexes **Ga1–Ga6**

Spectroscopic analyses confirmed the integrity and complexity of the coordination environment. The ^1^H and ^13^C­{^1^H}-NMR spectra of **Ga1–Ga6** (Figures S1–S12, Supporting Information) displayed two distinct sets of signals for the pyrazolyl rings, consistent with their mode of coordination and the presence of a stereogenic carbon C^a^ in the ligand backbone (**Ga1–Ga5**). The presence of chiral gallium centers led to potential diastereomeric mixtures, although a predominant diastereoisomer was obtained in most cases, as exemplified by the ^1^H NMR spectrum of **Ga5** (Figure 1). The predominance of a single diastereomer is consistent with the structurally defined octahedral coordination sphere imposed by the κ^3^-NNO scaffold, which restricts conformational flexibility and favors a thermodynamically preferred ligand arrangement around the octahedral Ga­(III) center. Minor low-intensity resonances observed in the ^1^H NMR spectra are consistent with the presence of a small amount of a secondary diastereomer and do not correspond to chemical impurities.

**Figure 1:**
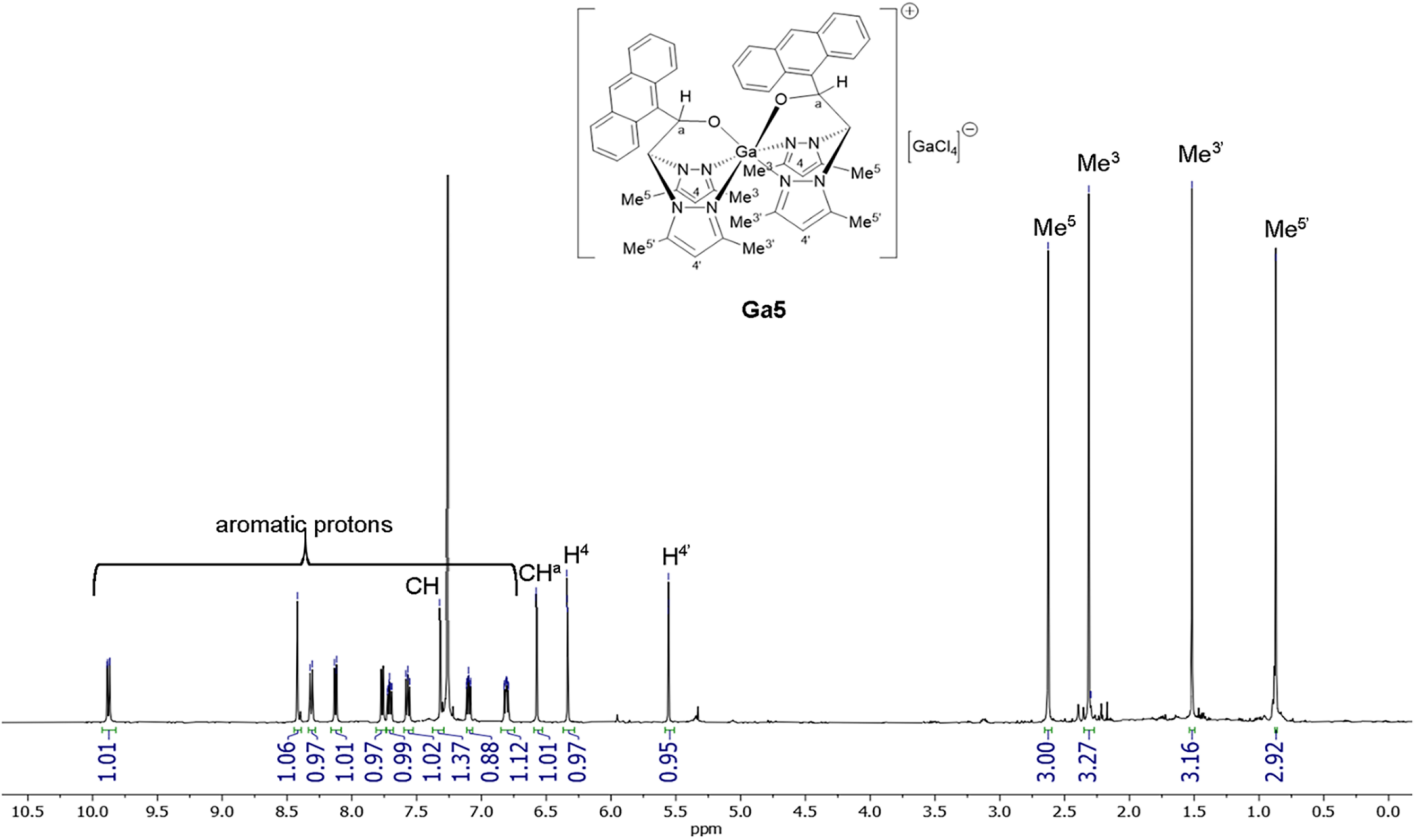
^1^H NMR spectrum of complex **Ga5** recorded in CDCl_3_ (400 MHz). Key resonances corresponding to the pyrazole and aromatic regions are highlighted.

To rigorously assess compound purity and confirm structural integrity, a comprehensive set of analytical techniques was employed. Elemental analyses (C, H, N) were performed for all gallium complexes (**Ga1–Ga6**). As summarized in Table S1 (Supporting Information), the experimentally determined values are in excellent agreement with those calculated for the proposed molecular formulations, supporting the assigned compositions and high bulk purity of the isolated materials.

High-performance liquid chromatography (HPLC) analyses were conducted for all Ga­(III) complexes (**Ga1–Ga6**). In each case, the chromatograms display a single peak corresponding to the target compound, with no detectable secondary signals above the established baseline threshold (Figures S13–S18). Purity was quantified by peak-area normalization, affording chromatographic purities exceeding 95% for all complexes, thereby confirming their suitability for biological evaluation.

High-resolution mass spectrometry (HRMS) further corroborated compound identity (Figures S19–S24). The spectra unambiguously exhibit the expected molecular ion corresponding to the intact cationic gallium species, accompanied by fragmentation patterns fully consistent with the proposed heteroscorpionate ligand framework. Minor signals observed at higher *m*/*z* values are attributable to low-abundance ionization-derived adducts or aggregates generated under electrospray ionization conditions, rather than to chemical impurities.

Single-crystal X-ray diffraction analysis provided unequivocal confirmation of the dual-metal architecture present in these salts. The ORTEP views of **Ga5** and **Ga6** (Figure 2) reveal ionic compounds featuring two distinct gallium environments: an octahedral cationic [Ga­(κ^3^-NNO)_2_]^+^ unit and a tetrahedral [GaCl_4_]^−^ anion. The gallium center at the cationic moiety exhibits an octahedral geometry with two heteroscorpionate ligands coordinated through the pyridinic nitrogen atoms of pyrazole rings and the oxygen atoms from the alkoxide groups in a cis disposition in a κ^3^-NNO coordination mode. Bond angles confirm a distorted octahedral geometry for the gallium cationic moiety. The maximum distortion is observed for the O(1)–Ga(1)–N(2) and O(1)–Ga(1)–O(2) angles with values of 97.1(1)° and 98.14(9)° for **Ga5** and **Ga6** complexes, respectively. In contrast, the second gallium center displays a tetrahedral geometry with four chloride ligands in an anionic counterion, with the dihedral angles between the Cl(1)–Ga(2)–Cl(4) and Cl(2)–Ga(2)–Cl(3) planes of 89.3° for **Ga5** and between the Cl(4)–Ga(2)–Cl(3) and Cl(1)–Ga(2)–Cl(2) planes of 88.1° for **Ga6**. Complexes **Ga5** and **Ga6** crystallized in the solid state as a racemic mixture of a single diastereoisomer. The structure of enantiomer Λ*RR* from complex **Ga5** and Λ from complex **Ga6** are shown in Figure 2.

**Figure 2:**
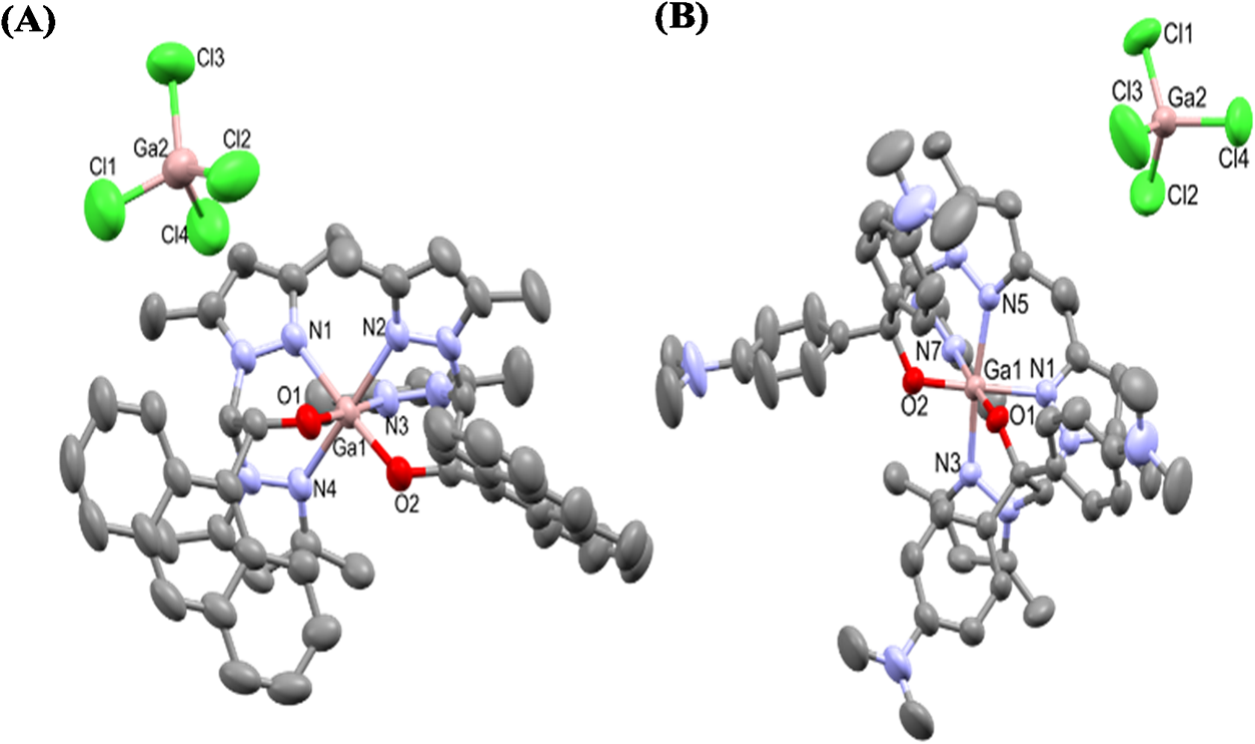
Crystal structure views for complexes [Ga­(κ^3^-bpzanthe)_2_]^+^[GaCl_4_]^−^ (**Ga5**, CCD reference number 2198294) (A) and [Ga­(κ^3^-bpzbdmape)_2_]^+^[GaCl_4_]^−^ (**Ga6**, CCD reference number 2498203) (B). Hydrogen atoms, crystallization solvent molecules, and **Ga6** disorders are omitted in terms of clarity. Thermal ellipsoids are drawn at the 50% probability level. Gallium, oxygen, carbon, nitrogen, and chloride atoms are represented in pink, red, gray, blue and green, respectively.

The crystallographic data and selected bond interatomic distances and angles are given in Tables S2–S6 of the Supporting Information. These solid-state data conclusively demonstrate the coexistence of two independent Ga­(III) sites within a single coordination framework, a unique structural feature that underpins the remarkable robustness and chemical stability of this new metallodrug family.

### Study of the Stability of Salt Gallium Complexes in Aqueous Media

Because metallodrugs are typically dissolved in aqueous or mixed DMSO/H_2_O media, where the organic cosolvent content must remain below 0.25% v/v to preserve cell viability, assessing both solubility and stability under such conditions is a critical step before biological testing. Therefore, the aqueous behavior of complexes **Ga1–Ga6** was systematically evaluated. The aqueous solubility of each compound was determined by UV–Vis spectroscopy using compound-specific calibration curves (see Experimental Section). According to the classification criteria of the International Pharmacopeia,^48,49^ the complexes displayed a wide solubility range depending on ligand substituents: **Ga1** (0.36 mg/mL) and **Ga5** (0.14 mg/mL) were classified as very slightly soluble, **Ga2** (1.67 mg/mL), **Ga4** (2.06 mg/mL), and **Ga6** (1.43 mg/mL) as slightly soluble, and **Ga3** (15.94 mg/mL) as sparingly soluble. This broad solubility spectrum illustrates how subtle ligand modifications, especially the incorporation of electron-donating NMe_2_ substituents, can significantly enhance water compatibility and, consequently, biological applicability. The **Ga3** complex, with a single dimethylamino-phenyl group, showed the highest solubility, while the anthracenyl-substituted **Ga5** was the least soluble due to its extended aromatic surface.

The stability of the complexes in biologically relevant solvents was investigated by ^1^H NMR spectroscopy in DMSO-*d*_6_ and in DMSO-*d*_6_:D_2_O (1:1) mixtures, as these conditions closely mimic those used in cell-based assays. Representative spectra are shown in Figures 3 and S25–S26 as representative examples of the series. All six complexes exhibited remarkable spectral stability, with no detectable hydrolysis or decomposition products even after several days at room temperature. The preservation of characteristic pyrazolyl and aromatic signals confirmed the structural integrity of the Ga­(III) coordination sphere in all cases.

**Figure 3:**
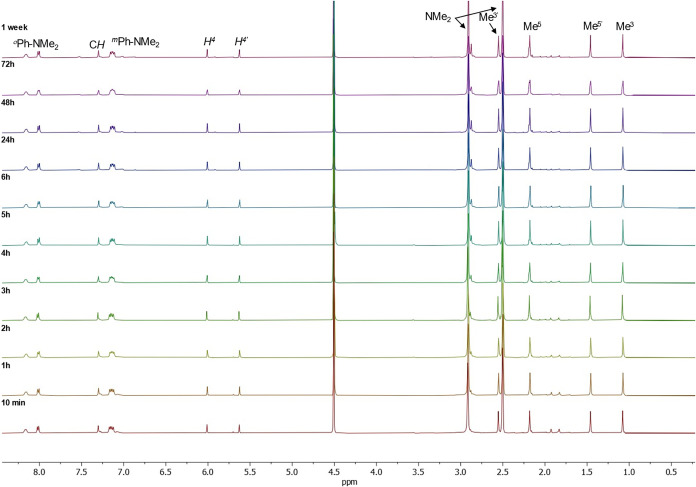
^1^H NMR spectra showing the stability of [Ga­(κ^3^ bpzbdmape)_2_]^+^[GaCl_4_]^−^ (**Ga6**) in DMSO-*d*_6_:D_2_O (1:1) over 1 week at room temperature.

Among the series, **Ga6**, bearing two para-dimethylaminophenyl substituents, displayed the highest resistance to dissociation under aqueous conditions, maintaining an unchanged NMR profile over 1 week. This superior hydrolytic stability could be attributed to intramolecular electronic donation from the amine substituents, which reinforces the Ga–O and Ga–N bonds and stabilizes the [Ga­(κ^3^-NNO)_2_]^+^ cationic core. Such behavior represents a notable advancement over conventional Ga­(III) complexes, which frequently undergo partial hydrolysis or ligand exchange in water, limiting their biomedical translation.^20,50,51^

Although clinically investigated Ga­(III) systems display sufficient stability for biological evaluation, preservation of a structurally defined coordination sphere under aqueous conditions remains critical for establishing reliable structure–activity relationships. In the present system, the heteroscorpionate κ^3^-NNO framework enforces a well-defined and kinetically persistent octahedral environment around Ga­(III), as confirmed by ^1^H NMR experiments in DMSO-*d*_6_/D_2_O (1:1), which showed no detectable ligand exchange or decomposition over 1 week.

The isolated compounds crystallize as [Ga­(κ^3^-NNO)_2_]^+^[GaCl_4_]^−^ salts; however, [GaCl_4_]^−^ is not stable in aqueous media and undergoes rapid chloride dissociation and hydrolysis to generate aqua and hydroxo Ga­(III) species under neutral or mildly acidic conditions.^20,21^ Consequently, under the biological assay conditions employed herein, the counterion is not expected to persist as an intact [GaCl_4_]^−^ species. The biologically relevant entity, therefore, corresponds to the kinetically robust heteroscorpionate [Ga­(κ^3^-NNO)_2_]^+^ cation, whereas the counterion primarily fulfills a structural role in the solid state.

To further correlate solubility and transport properties, the lipophilicity of **Ga1–Ga6** was determined by the shake-flask method, calculating the logarithmic octanol/water partition coefficient (log *P*) for each complex (see Experimental Section, Table S7, and Figure S27).^52^ The results demonstrated that the introduction of bulky aromatic substituents or dimethylamine groups systematically increased log *P* values, confirming the tunability of the molecular scaffold. This parameter is particularly relevant because it influences membrane permeability and intracellular accumulation, key determinants of in vitro activity. Overall, these findings highlight that the heteroscorpionate framework confers exceptional chemical robustness to Ga­(III) complexes, enabling their dissolution, handling, and biological testing under aqueous conditions without degradation, a critical experimental milestone rarely achieved in gallium-based coordination chemistry.^35,50^

### Preclinical In Vitro Evaluation of the Novel Gallium-Based Metallodrugs

#### Antiproliferative Activity of Heteroscorpionate Gallium­(III) Complexes in Breast Cancer Cell Lines

The antiproliferative activity of the six heteroscorpionate gallium­(III) complexes (**Ga1–Ga6**) was evaluated by MTT assays across a representative panel of human breast cancer cell lines encompassing distinct molecular subtypes: Triple-negative (BT549 and MDA-MB-231), HER2-positive (SKBR3), and Hormone receptor–positive (MCF7). For benchmarking, cisplatin, oxaliplatin, and carboplatin were included as classical platinum-based references (Figure 4). The resulting heatmaps summarize cell viability after 72 h of exposure, with blue intensity reflecting lower viability and thus higher cytotoxic potency. The data revealed pronounced differences in activity among the gallium complexes. **Ga3** and **Ga6** emerged as the most potent derivatives, exhibiting nanomolar IC_50_ values and outperforming all three platinum controls across the entire panel. Detailed IC_50_ data for each compound and cell line are presented in Table 1, and dose–response curves are provided in Figure S28 of the Supporting Information.

**Figure 4:**
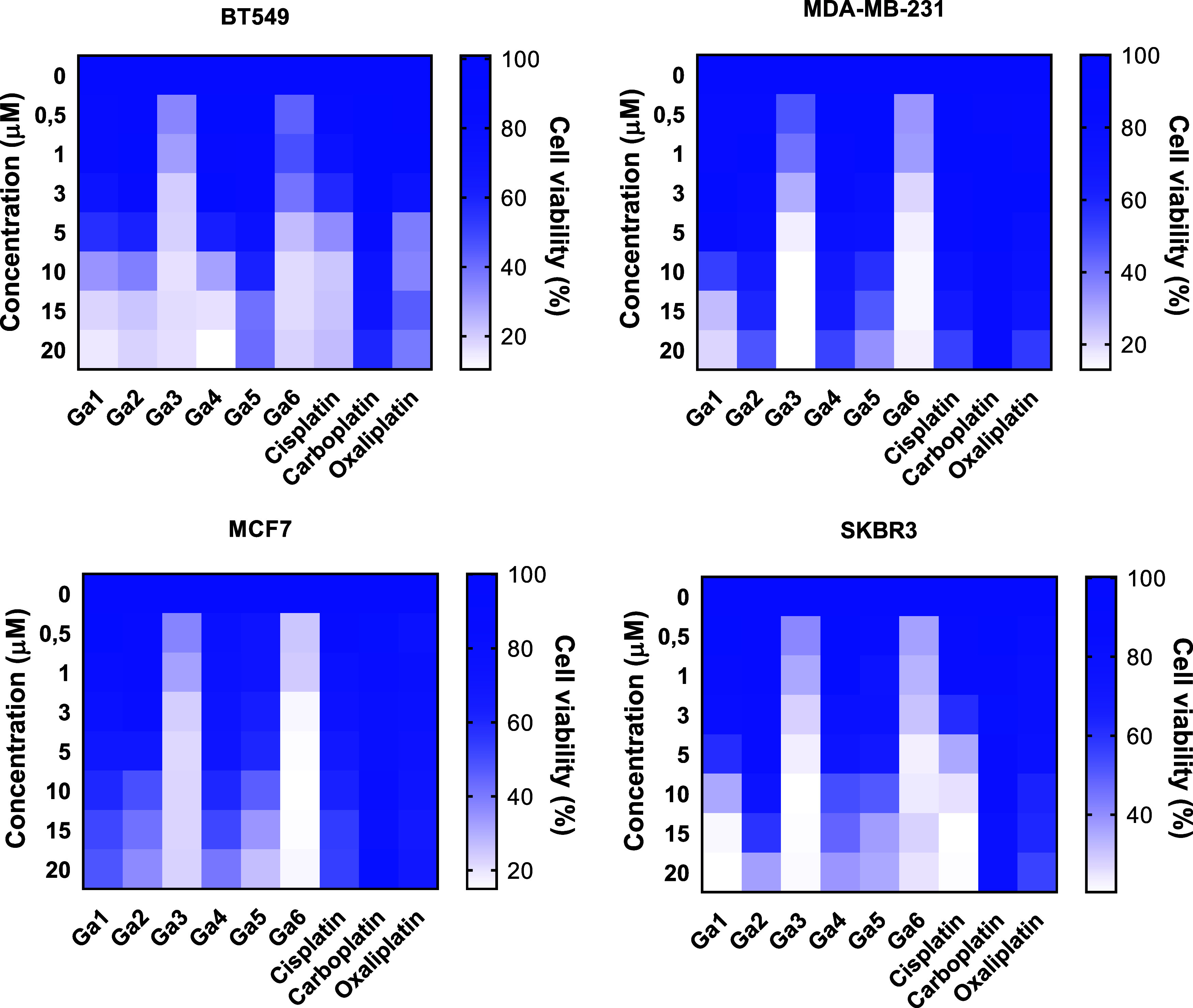
Heatmaps illustrating the antiproliferative effects of gallium complexes **Ga1–Ga6** and platinum-based drugs (cisplatin, carboplatin, and oxaliplatin) against four human breast cancer cell lines: BT549, MDA-MB-231, MCF7, and SKBR3. The color scale reflects relative cell viability after 72 h of treatment (white indicates lower viability). Data represent the mean ± SD from three independent experiments performed in triplicate.

**Table 1:** IC_50_ Values for the Gallium Complexes and Platinum References in Four Breast Cancer Cell Lines and Two Non-Trasformed Cell Lines^a^

	IC_50_ values ± SD (μM)					
Compound	BT549	MDA-MB-231	SKBR3	MCF7	HaCaT	MCF10A
**Ga1**	8.36 ± 0.849	12.20 ± 0.396	7.39 ± 0.906	10.59 ± 0.143		
**Ga2**	9.48 ± 0.030	16.75 ± 0.970	5.84 ± 1.825	8.36 ± 1.801		
**Ga3**	0.29 ± 0.022	0.19 ± 0.078	0.42 ± 0.034	0.18 ± 0.078	0.10 ± 0.021	0.07 ± 0.004
**Ga4**	6.89 ± 0.686	17.42 ± 0.983	18.98 ± 0.826	16.79 ± 2.795		
**Ga5**	12.97 ± 0.582	13.95 ± 1.643	13.10 ± 1.695	16.87 ± 0.849		
**Ga6**	0.39 ± 0.029	0.27 ± 0.057	0.43 ± 0.076	0.16 ± 0.070	0.24 ± 0.009	0.16 ± 0.066
**Cisplatin**	2.94 ± 0.959	16.99 ± 3.22	3.88 ± 0.810	19.47 ± 0.738	1.15 ± 0.134	3.79 ± 0.861
**Carboplatin**	>20	>20	>20	>20		
**Oxaliplatin**	5.03 ± 0.615	>20	>20	>20		

a Values are means ± SDs obtained by the MTT assay (exposure time: 72 h).

Both **Ga3** and **Ga6** contain electron-donating dimethylamino substituents, a feature likely responsible for their enhanced cytotoxicity through increased electronic density at the gallium center, strengthening metal–ligand bonds and facilitating cellular uptake.^10,53–55^ Notably, the complexes maintained their potency across all breast cancer subtypes, demonstrating a broad and subtype-independent mechanism of action.

To evaluate the safety profile and selectivity of the most active complexes, in vitro assays were extended to nontransformed epithelial cells, namely, HaCaT keratinocytes and MCF10A mammary epithelial cells, using cisplatin as a reference. As illustrated in Figure 5A, both **Ga3** and **Ga6** displayed IC_50_ values in nonmalignant cells that were comparable to or slightly higher than those observed in tumor lines. Although no complete reduction in off-target toxicity was detected relative to cisplatin, the gallium compounds maintained cytotoxic levels consistent with those expected for active anticancer agents, while avoiding signs of acute toxicity. It is important to note that the apparent toxicity of the gallium complexes in nontransformed cell lines is an expected outcome under 2D in vitro conditions, particularly for metal-based compounds.^56–58^ In monolayer cultures, cells are in direct and continuous contact with the compound, without the protective barriers, gradients, or clearance mechanisms present in vivo. Under these conditions, even clinically approved metallodrugs such as cisplatin or carboplatin display significant cytotoxicity toward healthy cells, reflecting an overestimation of nonspecific toxicity that does not necessarily correlate with systemic effects in living organisms. In addition, the absence of a tumor microenvironment and extracellular matrix in 2D cultures prevents differential uptake and retention phenomena that, in vivo, often favor the accumulation of metal complexes in malignant tissues.^56,58^ Therefore, the comparable IC_50_ values observed for **Ga3** and **Ga6** in nontumorigenic cells should be interpreted as a methodological limitation intrinsic to direct-contact 2D assays, rather than as evidence of true systemic toxicity.^56^ The subsequent in vivo experiments in zebrafish and murine models, which incorporate physiological distribution and metabolism, will provide a more realistic and reliable assessment of the biocompatibility and therapeutic window of these gallium complexes.

**Figure 5:**
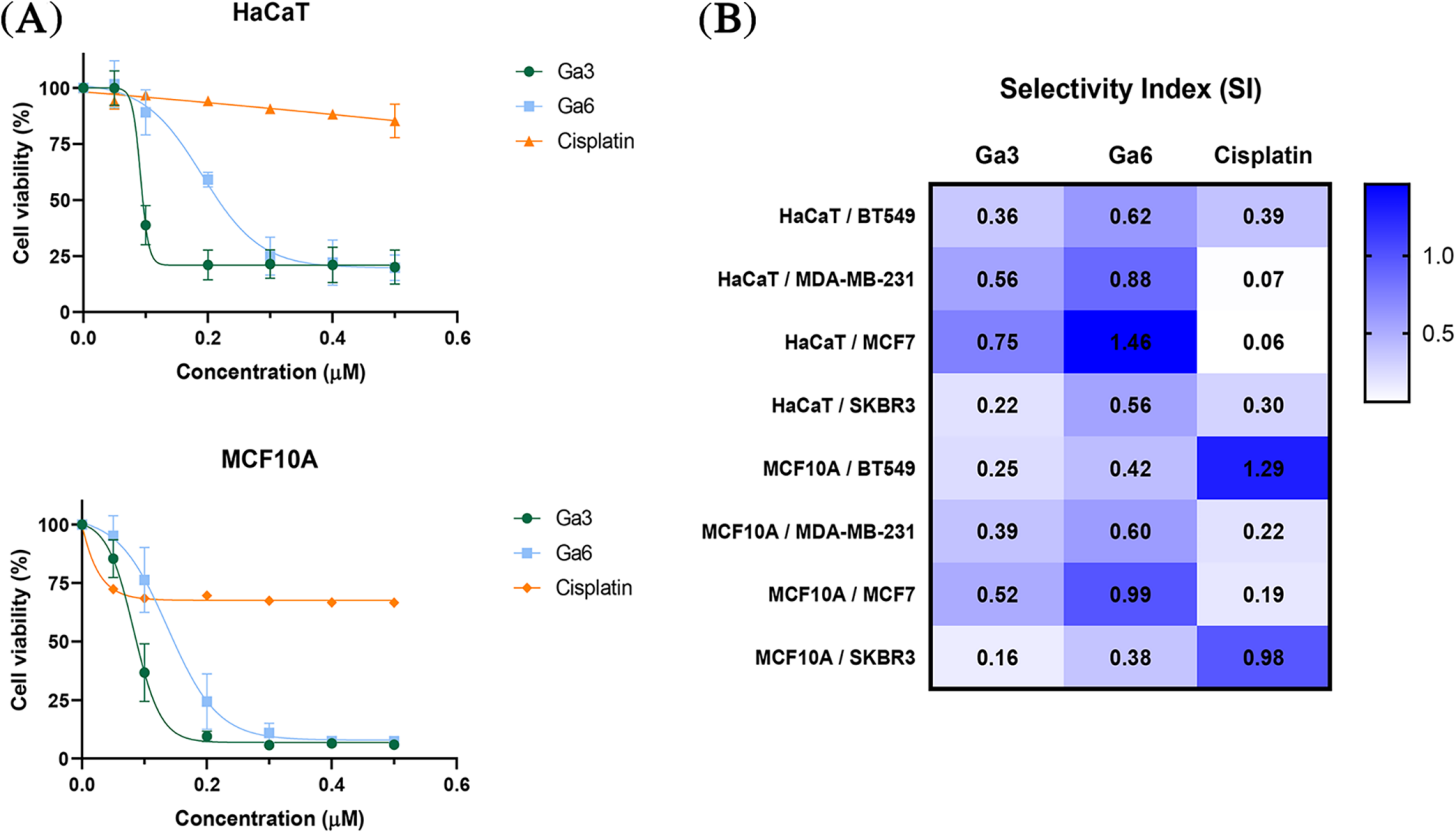
(A) Dose–response curves of **Ga3** and **Ga6** compared to cisplatin in nontransformed cell lines HaCaT and MCF10A. (B) Selectivity Index values for **Ga3**, **Ga6** and cisplatin. Data represent the mean ± SD from three independent experiments performed in triplicate.

To quantify tumor selectivity, the Selectivity Index (SI) was calculated as the ratio of SI = IC_50_(normal)/IC_50_(cancer).^59–61^ The **Ga6** complex showed SI values at least 1.5-fold higher than those of **Ga3** and equal to or superior to those of cisplatin (Figure 5B). This enhanced selectivity, coupled with its superior aqueous stability, may justify the choice of **Ga6** as the lead candidate for subsequent in vivo assessment.

#### Antiproliferative Activity of Heteroscorpionate Gallium­(III) Complexes in 3D Matrix Context

A Matrigel test, mainly composed of laminin and collagen proteins of the extracellular matrix, was conducted with the aim of generating a three-dimensional context closer to the nature of tumors.^62^ Both tumoral and nontumoral breast cell lines were grown in this matrix, promoting their ability to grow three-dimensionally by forming branched structures. Gallium­(III) compounds significantly reduce the size of the three-dimensional structures generated by MDA-MB-231 and MCF7 tumor cells compared to the nontreated control (Figure 6). This effect was maintained in the nontumor MCF10A cell line. These results, together with the previous ones, support the potent antitumoral activity of Heteroscorpionate Gallium­(III) Complexes and the need to evaluate toxicity in a living organism.

**Figure 6:**
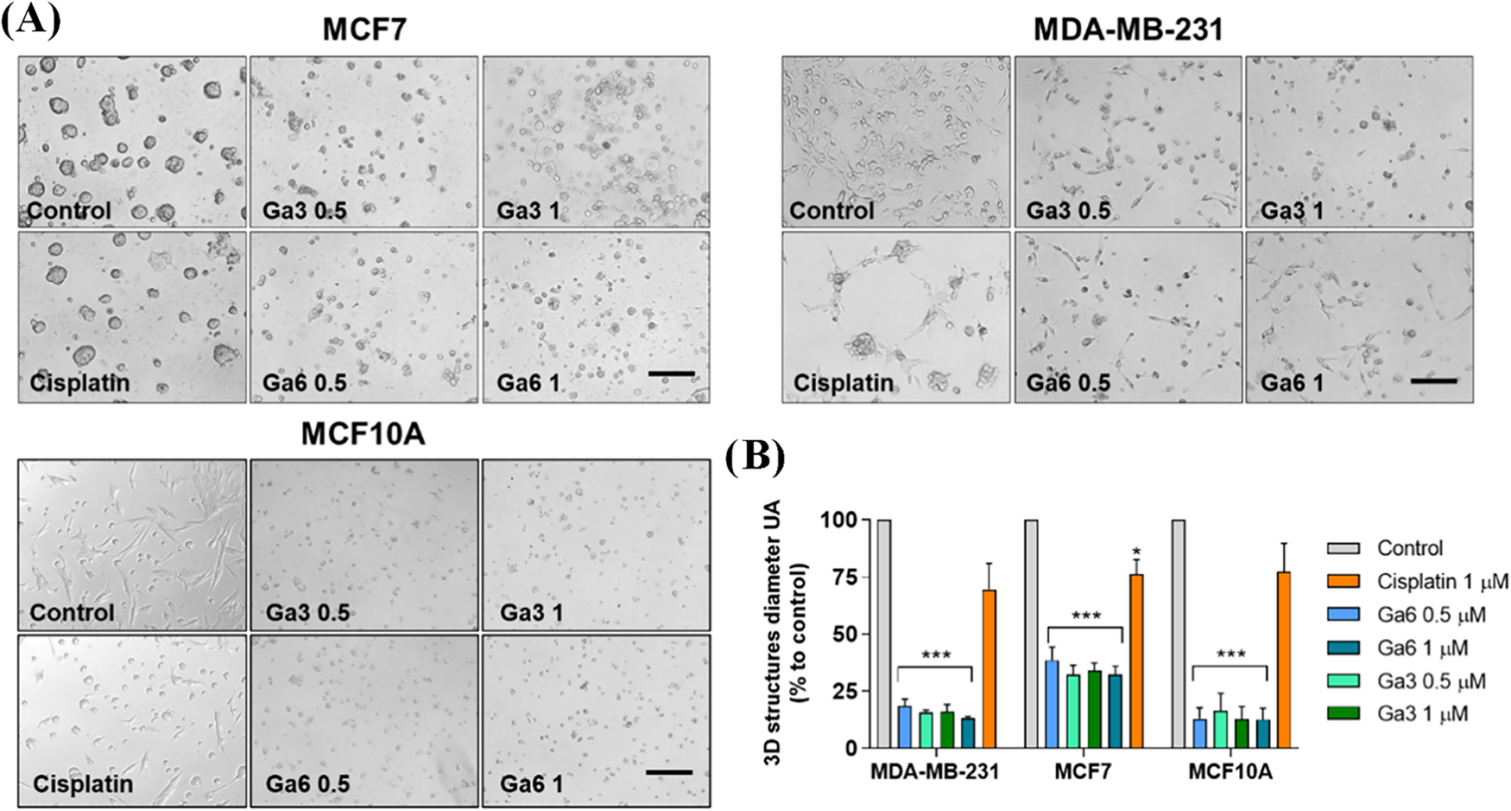
Matrigel assay. (A) 3D structures of MCF7, MDA-MB-231 and MCF10A seeded in Matrigel matrix and treated with compound at indicated doses for 72-h. Scale bar = 250 μm. (B) The diameter of 3D structures was quantified using ImageJ. Data are the average ± SEM of three independent experiments. The values for the statistical analyses are * *p* ≤ 0.05; *** *p* ≤ 0.001.

#### Assessment of Mechanism of Action: ROS Generation, GSH/GSSG Content, Redox-Independent Cytotoxicity and Mitochondrial Dysfunction by Interference with Iron Metabolism

Given that many metal-based drugs exert cytotoxicity via redox-mediated mechanisms,^3,63,64^ the potential involvement of reactive oxygen species (ROS) in the activity of **Ga1–Ga6** was investigated.^46^ ROS generation assays were performed in MDA-MB-231 and MCF7 cells using H_2_O_2_ as a positive control and cisplatin as a reference metallodrug. Each compound was tested at its respective IC_50_ concentration. As shown in Figure 7A, none of the gallium complexes produced significant ROS levels relative to the negative control, indicating that their cytotoxic mechanism is not linked to oxidative stress. These results suggest that the antiproliferative effect of **Ga3** and **Ga6** arises from direct biochemical interactions rather than nonspecific redox activity, which is advantageous for minimizing oxidative side effects.

**Figure 7:**
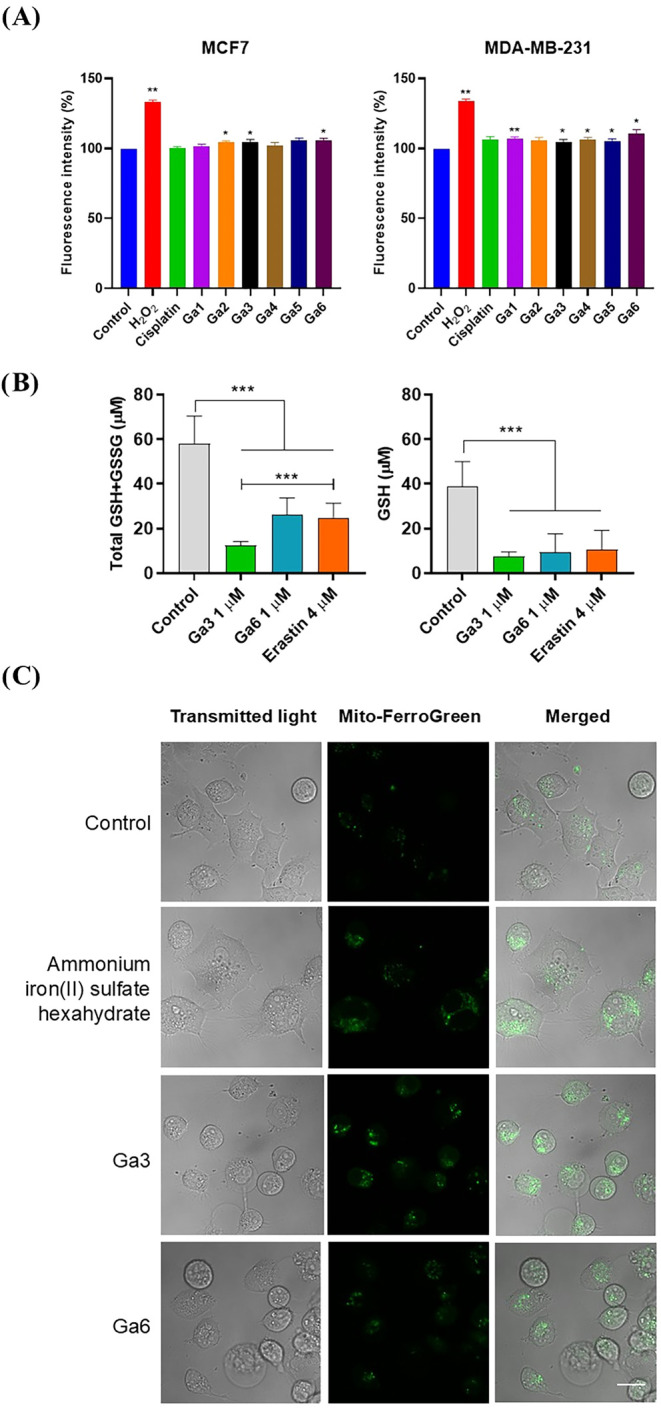
(A) Fluorescence Intensity (%) as a measurement of ROS generation by **Ga1–Ga6**, compared to cisplatin and H_2_O_2_ as a positive control. (B) The total GSH + GSSG and GSH level (in μM) was analyzed with a GSH assay kit under 24-h treatment with **Ga3 and Ga6** (1 μM) and Erastin (4 μM) as a positive control in MDA-MB-231 cells. (C) Mitochondrial Fe^2+^ was determined by Mito-FerroGreen probe after 6-h treatment with **Ga3 and Ga6** (1 μM); Ammonium iron­(II) sulfate hexahydrate was used as a positive control. Cells were observed in a confocal microscope. Scale bar = 40 μm. Data are the average ± standard deviation (SD) of three independent experiments performed in triplicate. To determine significant statistical differences, a Student’s *t* test was used. The values for the statistical analyses are * *p* ≤ 0.05; ** *p* ≤ 0.01; *** *p* ≤ 0.001.

Previous studies have reported that certain gallium compounds interfere with iron homeostasis, ultimately leading to mitochondrial dysfunction.^65–68^ In view of the superior cytotoxic potency observed for **Ga3** and **Ga6**, these two derivatives were selected for further mechanistic investigation. First, the total cellular GSH/GSSG and GSH levels were quantified after 24 h treatment with **Ga3** and **Ga6**. As depicted in Figure 7B, the total GSH/GSSG and GSH level was significantly lower than control nontreated cells and similar than Erastin-treated cells. Next, the release of intramitochondrial ferrous iron (Fe^2+^) was assessed using the Mito-FerroGreen fluorescent probe and confocal microscopy. Ammonium iron­(II) sulfate hexahydrate was used as a positive control. As shown in Figure 7C, treatment with **Ga3** and **Ga6** resulted in a significant increase in mitochondrial Fe^2+^ fluorescence intensity compared to untreated cells. These findings support that **Ga3** and **Ga6** compounds induce a depletion of intracellular GSH, which disrupts mitochondrial redox buffering and iron–sulfur cluster stability, leading to accumulation of mitochondrial labile Fe^2+^. This iron dysregulation promotes mitochondrial dysfunction and activation of the intrinsic caspase-dependent apoptotic pathway, independently of detectable global ROS production.^69^

#### Analysis of Cell-Cycle Perturbations and Apoptosis Response Induced by Ga­(III) Complexes

To elucidate the mechanistic basis of growth inhibition, cell-cycle analysis was conducted in MDA-MB-231 and MCF7 cells following 24 h of treatment with **Ga3** (0.2 μM and 0.4 μM), **Ga6** (0.5 μM and 1 μM), and cisplatin (1 μM) as a control. Cells were stained with propidium iodide in the presence of RNase to quantify DNA content. The results, summarized in Figures 8A and S29, revealed that both gallium complexes induced a significant accumulation of cells in the G0/G1 phase, indicative of cell-cycle arrest at this checkpoint. In MCF7 (ER^+^/PR^+^) cells, **Ga3** and **Ga6** caused a more pronounced G0/G1 arrest than cisplatin, which produced a broader, less specific distribution across phases. In MDA-MB-231 (TNBC, Triple Negative Breast Cancer) cells, the G0/G1 accumulation was moderate but still greater than that induced by cisplatin. The concomitant decrease in the S-phase population suggests interference with DNA replication and progression into mitosis. Collectively, these results demonstrate that **Ga3** and **Ga6** act as potent inhibitors of cell-cycle progression, with a mechanistic profile distinct from classical platinum agents.

**Figure 8:**
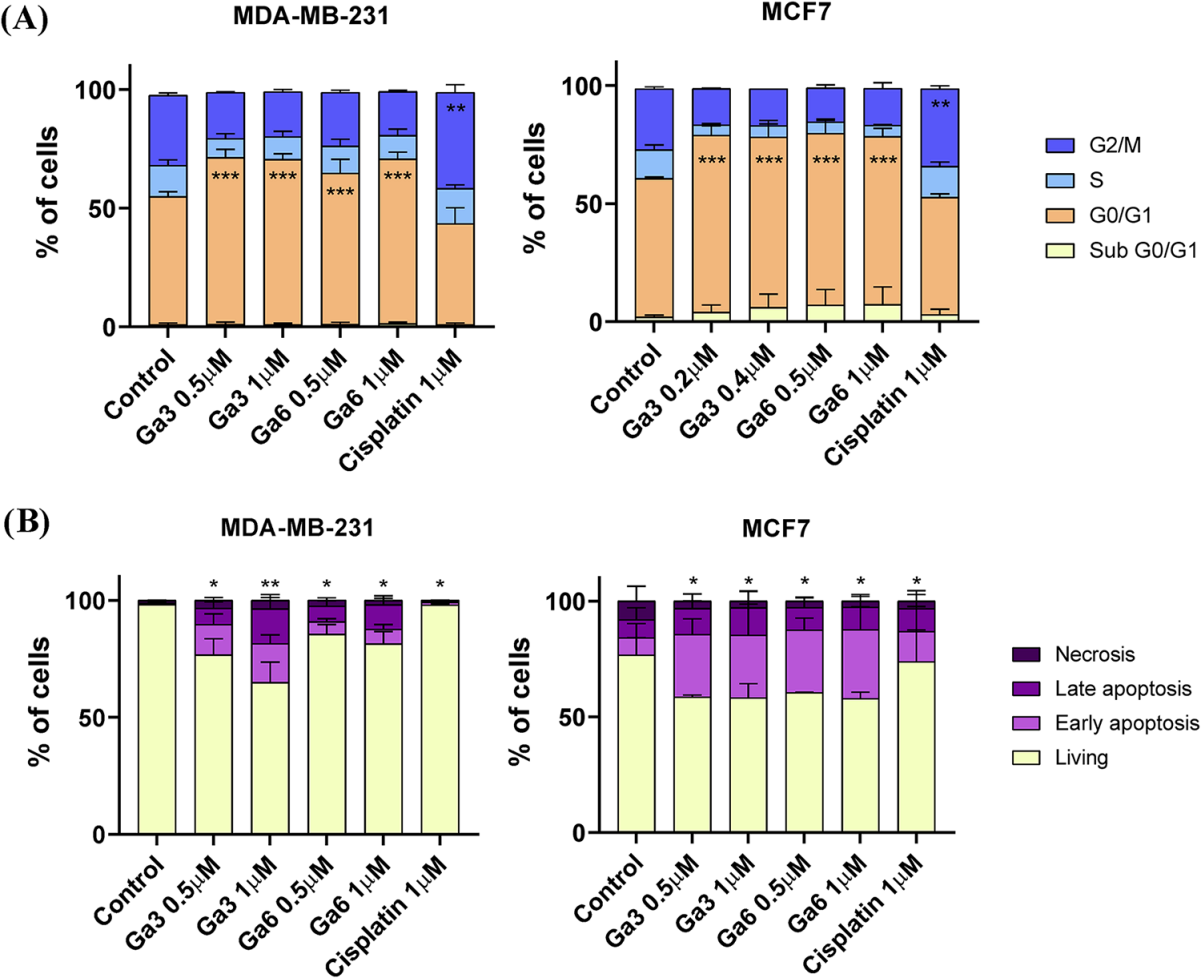
(A) Cell cycle analysis of MDA-MB-231 and MCF7 cell lines after 24h of treatment with **Ga3** and **Ga6**. (B) Apoptosis analysis of MDA-MB-231 and MCF7 cell lines after 72h of treatment with **Ga3** and **Ga6**. Cisplatin was tested at 1 μM, a concentration below its reported IC_50_ value (≈8–12 μM) in MDA-MB-231 cells; therefore, only a limited effect on cell viability and apoptosis is expected under these conditions. Data are the average ± standard deviation (SD) of three independent experiments performed in triplicate. To determine significant statistical differences, Student’s *t* test was used. The values for the statistical analyses are *, *p* ≤ 0.05; **, *p* ≤ 0.01; ***, *p* ≤ 0.001.

The apoptotic response to **Ga3** and **Ga6** was subsequently examined by Annexin V/Propidium Iodide flow cytometry after 72 h of treatment (0.5 μM and 1 μM, respectively). As illustrated in Figures 8B and S30, both complexes markedly increased the proportion of early and late apoptotic cells relative to untreated controls and cisplatin. The apoptotic effect was particularly pronounced in MCF7 cells, consistent with their greater sensitivity observed in viability assays. Importantly, the necrotic population remained minimal, confirming that cell death was predominantly apoptotic rather than necrotic.

Administration of a pan-caspase inhibitor Q-VD-OPh reverted apoptosis (Figure S31), suggesting that the mechanism was mainly caspase-dependent which, together with previous results, supports the idea that gallium compounds cause mitochondrial dysfunction that leads to the induction of the intrinsic pathway of apoptosis.

These results reinforce the hypothesis that the gallium complexes act through programmed cell death pathways, offering a mechanistic advantage over cisplatin, which often induces broader, less selective cytotoxicity.

Taken together, the in vitro data highlight several key advances achieved by this new family of Ga­(III) metallodrugs: (1) Exceptional aqueous stability, enabling their reliable evaluation under physiological conditions; (2) Potent, nanomolar-range cytotoxicity surpassing platinum standards; (3) Subtype-independent antitumor activity across diverse breast cancer models; (4) Predominant induction of apoptosis via G0/G1 cell-cycle arrest, without ROS involvement; (5) Improved selectivity indices, particularly for the **Ga6** complex.

These findings position **Ga3** and **Ga6**, especially **Ga6**, as the most promising candidates for preclinical translation, combining high potency with structural stability, a combination seldom achieved in gallium coordination chemistry.

### Preclinical In Vivo Evaluation of the Novel Gallium-Based Metallodrugs

Given the outstanding in vitro activity and stability of the heteroscorpionate gallium complexes, **Ga6**, the most potent and selective derivative, was selected for preliminary in vivo evaluation of toxicity and antitumor efficacy. The study employed zebrafish (Danio rerio) embryos as an alternative vertebrate model, offering several practical and ethical advantages over conventional rodent systems.^70^ Zebrafish combine high genetic and physiological homology with humans, transparency that allows noninvasive imaging, and the capacity to absorb compounds directly from the surrounding medium. These features make them ideally suited for early stage pharmacological screening and toxicity assessment, providing robust statistical data while significantly reducing the number of higher vertebrates required for drug development.^71–74^

#### Acute Toxicity and Determination of LC_50_ and LC_10_ Values in Zebrafish Embryos

The acute toxicity of **Ga6** was determined following the OECD Test No. 236 (Fish Embryo Acute Toxicity Test) guidelines.^75^ Fertilized embryos at 4 h postfertilization (hpf) were exposed to increasing concentrations of **Ga6** (0–8 μM) at 28 °C, and lethality was assessed at 24, 48, 72, and 96 hpf. Four apical parameters were monitored: egg coagulation, lack of somite formation, failure of tail detachment, and absence of heartbeat. Among these, only embryo coagulation was observed at 24 hpf in the highest concentrations tested; no additional morphological alterations were detected up to 96 hpf, (Figure 9A).

**Figure 9:**
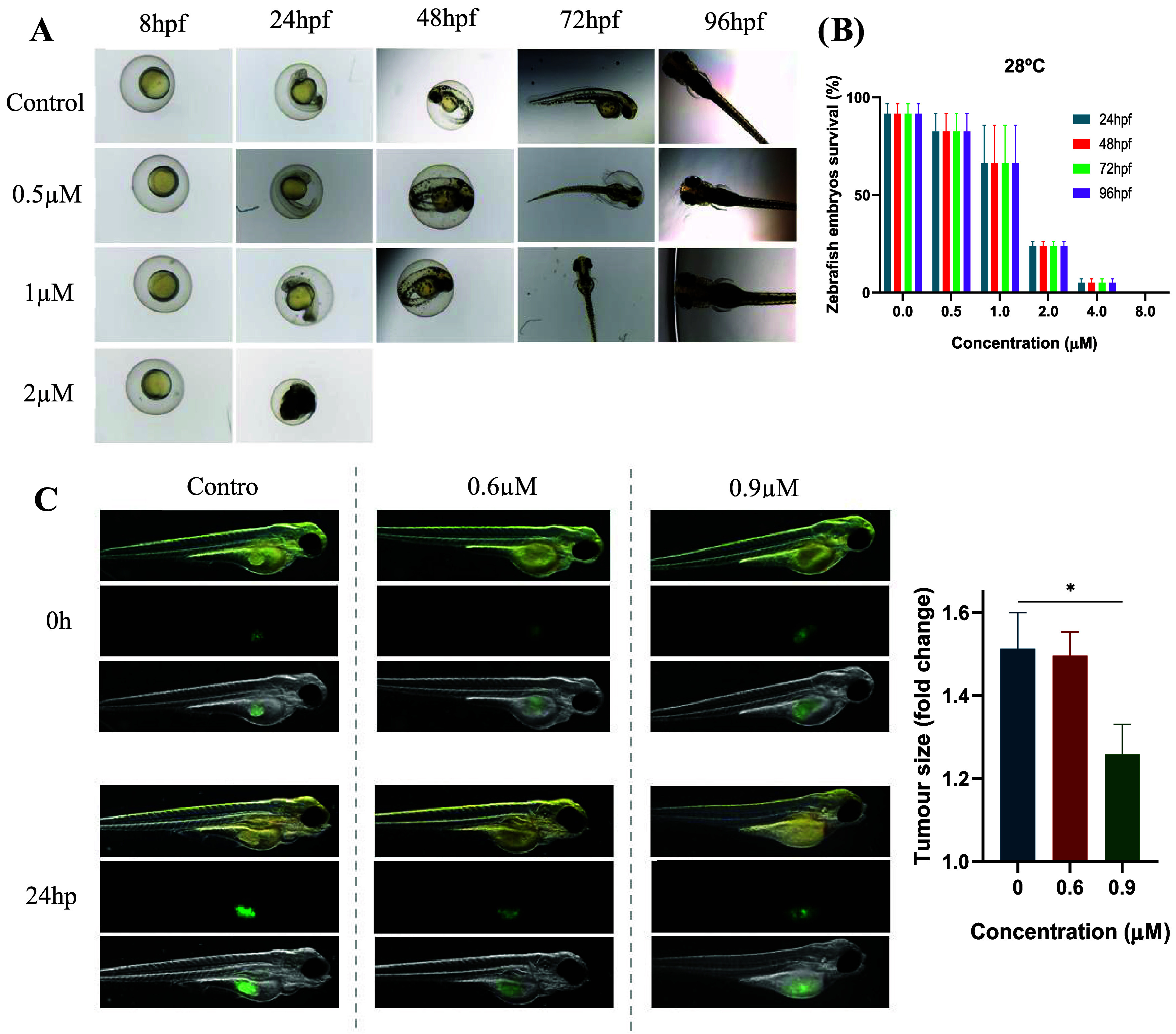
(A) Evolution and survival rate of zebrafish embryos exposed to different concentrations of **Ga6** at 28 °C up to 96hpf. For each experiment 168 embryos were used, divided into the different groups (*n*_total_ = 504 embryos). (B) Dose-dependent effects of **Ga6** on zebrafish embryo survival at 28 °C. (C) Antitumor activity of Ga6 in a zebrafish xenograft model. *Left panel:* Representative fluorescence micrographs of xenografts at 0 and 24 h post-treatment (hpt). *Right panel:* Quantification of tumor progression, expressed as fold change in fluorescence intensity relative to the 0 hpt baseline. For each experiment 72 embryos were used, divided into the different groups (*n*_total_ = 216 embryos). Data are presented as mean ± SEM. To determine significant statistical differences, ANOVA Dunnett’s multiple comparison test was used. The value for the statistical analyses is *, *p* ≤ 0.05.

Figure 9B shows the dose-dependent effects of **Ga6** on zebrafish embryo survival. Bar graph shows the percentage of surviving zebrafish embryos exposed to increasing concentrations of **Ga6** (0, 0.5, 1.0, 2.0, 4.0, and 8.0 μM) at four developmental time points: 24, 48, 72, and 96 hpf. Quantitative analysis revealed LC_50_ and LC_10_ values of 1.539 μM and 0.640 μM, respectively. These thresholds are 3–9 times higher than the IC_50_ values obtained in tumor cell lines, confirming that **Ga6** exerts strong antitumor effects at concentrations well below its embryotoxic range. The absence of a significant increase in lethality after 24 hpf is noteworthy. Embryos at initial developmental stages have been traditionally considered at critical life stages in zebrafish embryos^76^ and sensitivity to cytotoxic agents has been reported to be dependent on the developmental stage of the embryo when exposed to the cytotoxic agent.^77^ While some substances show increased toxicity after hatching (>48hpf), likely due to access limitations imposed by the chorion, particularly for compounds with a very high molecular weight,^78^ many anticancer agents exert their highest toxicity during early stages such as gastrulation.^79^ This observed plateau in lethality for **Ga6** aligns with the latter pattern and can be interpreted within the context of **Ga6**’s antitumor activity, which targets rapidly dividing cells. At 24 hpf, the zebrafish embryo consists predominantly of highly proliferative, less specialized blastomeres, which share genetic, metabolic, and proliferative characteristics with tumor cells,^80^ making them particularly susceptible.^79^ As development proceeds, cellular differentiation increases, and the population of such highly vulnerable, undifferentiated cells decreases. Consequently, embryos that survive the initial 24-h exposure likely represent a population that is either less susceptible due to natural biological variation or has passed the most vulnerable window, leading to the observed plateau in toxicity. This interpretation is further supported by the fact that the calculated LC_50_ in embryos is higher than the values obtained in homogeneous, actively cycling tumor cell lines in vitro. The latter represents a pure culture of immortalized, neoplastic cells, explaining their heightened sensitivity compared to the complex, developing embryo, where tissue architecture, emerging compensatory mechanisms, and cellular heterogeneity confer relative protection at later stages. An additional assay conducted at 34 °C (see the survival rate in Figure S32 of the Supporting Information), to mimic the temperature used in xenograft experiments, produced comparable values (LC_50_= 1.564 μM; LC_10_ = 0.551 μM), further supporting the compound’s stability and predictable dose–response behavior under physiological conditions.

#### Antitumoral Efficacy in Zebrafish Xenografts

The antitumor efficacy of **Ga6** was evaluated in zebrafish embryos xenografted with GFP-expressing MDA-MB-231 breast cancer cells. This system provides a relevant, live imaging model that allows the quantification of tumor mass over time in a transparent, vascularized host. After microinjection of tumor cells into the yolk of 48 hpf embryos and incubation at 34 °C for 24 h, the xenografted larvae (72 hpf) were divided into three treatment groups: control, 0.6 μM **Ga6** (LC_10_), and 0.9 μM **Ga6** (1.5 × LC_10_).

Tumor progression was quantified by measuring the fluorescence intensity of the xenografted area at 0 and 24 h post-treatment (hpt) and expressed as fold change relative to the initial (0H) measurement. As shown in Figure 9C, the control and 0.6 μM groups displayed similar increase in tumor size (∼1.5-fold), whereas the 0.9 μM **Ga6** treatment produced a marked reduction in tumor growth (∼1.25-fold), demonstrating a clear dose-dependent response. Importantly, no differences in embryo survival or morphological abnormalities were detected at these concentrations, indicating that the antitumor effect occurred within the nontoxic range. These results confirm that **Ga6** retains its biological activity under in vivo conditions and efficiently limits tumor expansion in a living vertebrate system. Future validation in immunocompromised mouse xenograft models will be essential to further characterize the therapeutic potential and pharmacokinetic profile of **Ga6**.

#### Preliminary Toxicity in Murine Models

To further evaluate systemic tolerance, BALB/c nu/nu mice were used for a short-term toxicity assessment of **Ga6**, as the lead compound of the series. Two dosage regimens (2.5 mg kg^–1^ and 5 mg kg^–1^, administered intraperitoneally once weekly for 3 weeks) were compared to a vehicle control consisting of 10% (2-hydroxypropyl)-β-cyclodextrin in water. Throughout the 21-day study, no statistically significant body weight loss or signs of distress were observed in any treated group (Figure 10). Minor variations in individual weights remained within physiological limits and showed no dose-dependent trend. The absence of behavioral or morphological alterations suggests that **Ga6** does not induce overt systemic toxicity under the tested conditions.

**Figure 10:**
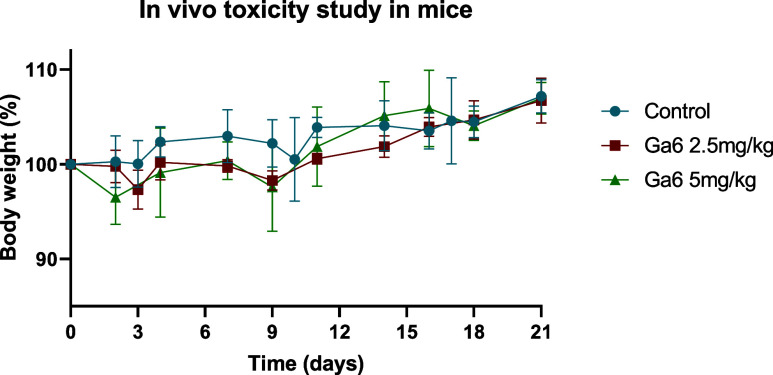
In vivo toxicity evaluation of **Ga6** in mice at two different doses, 2.5 mg/kg (*n* = 3) and 5 mg/kg (*n* = 3), over 21 days of experiment, and compared to a control group without treatment (*n* = 3). Data represent the mean ± SD across replicates.

This favorable safety profile, combined with the nanomolar in vitro potency and stability of **Ga6**, highlights a rare balance between efficacy and tolerability among gallium-based metallodrugs. Ga­(III) compounds are known for suffering hydrolysis processes that hinder their systemic administration,^20^ whereas the heteroscorpionate framework used here confers sufficient robustness to withstand biological conditions without decomposition. The capacity to evaluate a Ga­(III) complex from in vitro cell culture to in vivo xenograft and mammalian models represents a significant experimental milestone, reinforcing the translational potential of this family of compounds.

### Cellular Uptake and Intracellular Localization

**Ga5** was selected for cellular uptake and imaging studies due to the presence of the anthracene moiety, which provides intrinsic fluorescence and enables direct visualization of intracellular localization. Although **Ga6** was identified as the most potent compound, it lacks a fluorescent handle; therefore, **Ga5** was employed as a structurally analogous fluorescent surrogate of the heteroscorpionate Ga­(III) scaffold.

The absorption and emission spectra of **Ga5** (Figure S33 of the Supporting Information) show a broad absorption band centered at approximately 210 nm, characteristic of metal-to-ligand charge-transfer (MLCT) transitions, and a structured fluorescence emission between 350 and 550 nm, arising exclusively from the anthracene moiety.^46,81,82^ Upon excitation at 368 nm, **Ga5** displayed a monoexponential fluorescence decay with a lifetime of 4.5 ns in aqueous solution, indicative of a photophysically stable emissive state. This robust and well-defined fluorescence signal allowed for reliable quantification of the compound’s intracellular behavior using Fluorescence Lifetime Imaging Microscopy (FLIM), a highly sensitive technique capable of distinguishing chemical environments based on lifetime variations rather than intensity alone.^81^

FLIM imaging was carried out in T47D luminal breast cancer cells, enabling direct visualization of **Ga5** and its free ligand under identical conditions. The fluorescence lifetime pseudocolor maps (Figure 11A,C) were scaled from green (τ ≈ 4 ns) to red (τ ≈ 7 ns) to visualize local lifetime heterogeneity across the cytoplasm. Both **Ga5** and its ligand were efficiently internalized by the cells and exhibited a predominantly cytoplasmic distribution, with no detectable nuclear accumulation, even after prolonged incubation (1–24 h).

**Figure 11:**
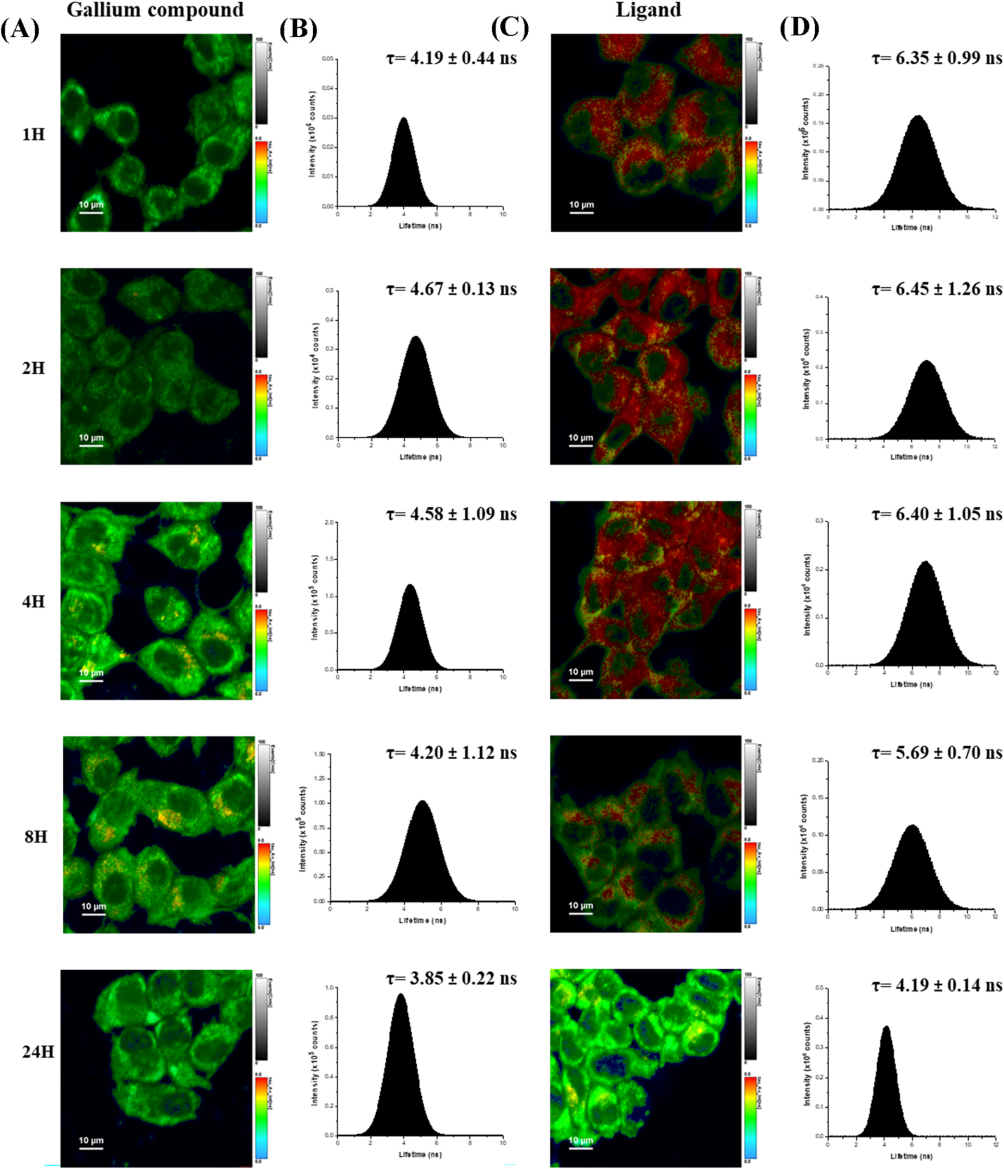
(A) FLIM images of T47D cells treated with the gallium compound at various incubation times; (B) fluorescence lifetime distribution histograms with τav (±2σ) values corresponding to the FLIM images in panel (A); (C) FLIM images of T47D cells treated with the ligand at the same incubation times as those treated with the gallium compound; (D) fluorescence lifetime distribution histograms with τav (±2σ) values corresponding to the FLIM images in panel (C).

Quantitative lifetime histograms (Figure 11B,11D) provided further mechanistic insight. After 1 h of incubation, **Ga5** displayed a narrow monomodal distribution centered at 4.2 ns, consistent with a single, well-defined emissive species within a homogeneous microenvironment. In contrast, the free ligand exhibited a broader, multimodal distribution centered around 6.4 ns, suggesting the coexistence of monomeric and aggregated emissive states. Over time, the ligand’s histogram gradually narrowed and shifted toward shorter lifetimes (∼4 ns), likely due to aggregation and subsequent quenching processes during intracellular accumulation. Remarkably, **Ga5** maintained a stable monoexponential fluorescence lifetime (∼4.2 ns) throughout the 24-h incubation, confirming that its photophysical properties remain unaltered upon cellular internalization. This value is consistent with the characteristic lifetime of free anthracene reported in other metal–organic complexes where no direct charge transfer to the metal center takes place, further supporting that the emissive state of **Ga5** arises predominantly from the ligand-centered transition.^46^ Conversely, the free ligand appears to undergo aggregation processes during initial uptake, where local accumulation promotes anthracene–anthracene interactions, leading to a longer average lifetime and a multimodal distribution attributed to the coexistence of monomeric and dimeric species, as previously reported in the literature.^82^

These facts reflect the structural rigidity and steric protection afforded by the heteroscorpionate framework, which prevents π–π stacking and nonradiative deactivation processes typical of free anthracene derivatives. Consequently, **Ga5** exhibits exceptional photostability and environmental homogeneity inside cells, validating its use as a reliable fluorescent reporter for gallium-based metallodrugs.

Taken together, these results demonstrate that heteroscorpionate coordination not only stabilizes gallium­(III) in aqueous and biological media but also preserves the emissive properties of the incorporated chromophore, enabling direct optical monitoring of metal–ligand complexes in live cells. This dual chemical and photophysical stability represents a significant advancement in the design of Ga-based metallopharmaceuticals, bridging inorganic synthesis, photophysics, and bioimaging within a single molecular platform.

## Conclusions

This study describes the design, synthesis, and biological evaluation of a new family of alkoxide-functionalized heteroscorpionate Ga­(III) complexes, introducing a structurally defined κ^3^-NNO coordination platform for gallium-based therapeutics. Comprehensive synthetic, spectroscopic, crystallographic, and analytical characterization established the formation of discrete, air- and moisture-stable Ga­(III) architectures suitable for biological investigation.

Systematic modulation of ligand substituents enabled controlled variation of lipophilicity, solubility, and aqueous stability, which translated into distinct cytotoxic profiles across multiple breast cancer subtypes. Within this series, **Ga3** and **Ga6** displayed nanomolar antiproliferative activity and favorable selectivity indices relative to cisplatin, while maintaining coordination integrity under biologically relevant conditions.

The lead compound **Ga6** further demonstrated selective cellular uptake, cytoplasmic localization, and significant tumor growth inhibition in zebrafish xenografts, together with preliminary tolerability in murine models. These findings highlight the heteroscorpionate framework as a robust and tunable coordination environment capable of supporting biologically active Ga­(III) complexes.

Collectively, this work expands the coordination chemistry toolbox available for gallium-based drug design and provides a foundation for the continued development of structurally defined Ga­(III) metallodrugs with improved physicochemical control and preclinical potential.

## Experimental Section

### General Procedures

The synthesis of organometallic compounds has been carried out under an inert atmosphere, using N-50 type nitrogen, supplied by Air Liquide (O_2_ < 2 ppm; H_2_O < 2 ppm; CO/CO_2_ < 0.5 ppm). To maintain these conditions, the synthesis of organometallic complexes has been carried out in a vacuum line provided with double shut-off valves (vacuum/inert gas), employing Schlenk or GloveBox techniques, in a Braun Labmaster glovebox. Elemental analyses were performed using a PerkinElmer 2400 CHN analyzer. The solvents employed for all the preparations have been purified and dried according to the methods described in the bibliography,^83^ distilled and stored under an inert atmosphere before their use. Deuterated solvents were stored over activated 4 Å molecular sieves and degassed by several freeze–thaw cycles. NMR spectra were acquired in two different spectrometers: Bruker Advanced 11.7 T (500 MHz for ^1^H NMR and 126 MHz for ^13^C NMR) and Bruker Advanced 9.4 T (400 MHz for ^1^H NMR and 101 MHz for ^13^C NMR). Chemical shifts (δ) are expressed in ppm with respect to TMS, taking as internal reference the deuterated solvent signals. Coupling constants (*J*) are expressed in Hz. The UV–vis absorption spectra were recorded at room temperature using a JASCO V-750 Spectrophotometer. Trimethylacetaldehyde, ferrocenecarboxaldehyde, 4-(dimethylamino)­benzophenone, 1-naphthaldehyde, 9-anthracenecarboxaldehyde and 4,4′-bis­(dimethylamino)-benzophenone were purchased from Sigma-Aldrich. Ligands were synthesized as previously described in literature.^47^

### Synthesis and Characterization

#### Synthesis of [Ga­(κ^3^-bzpbe)_2_]^+^[GaCl_4_]^−^ (**Ga1**)

In a 100 cm^3^ Schlenk tube, bpzbeH (1 g, 3.4 mmol) was dissolved in 30 mL of dry THF and cooled down to −78 °C. Then, a solution of *^n^* BuLi (1.6 M in hexane, 2.0 mL, 3.62 mmol) was added dropwise, and the reaction mixture was stirred for one hour at room temperature. After that time, the lithiated adduct was transferred via cannula to a precooled (0 °C) suspension of GaCl_3_ (0.64 g, 3.62 mmol) in 20 mL of THF. The resulting mixture was warmed to room temperature and left stirring overnight. Solvent was then removed in vacuo and the remaining solid was taken up in CH_2_Cl_2_ and filtered through Celite to eliminate the LiCl salt generated. Solvent was again removed under vacuum. Complex Ga1 was obtained as a white solid. Yield: 2.50 g (85%). Anal. Calcd for C_32_H_50_Cl_4_Ga_2_N_8_O_2_: C, 44.7; H, 5.9; N, 13.0. Found: C, 44.9; H, 6.2; N, 12.9. ^1^H NMR (400 MHz, CDCl_3_) δ 6.42 (s, 2H, C*H*), 6.07, 5.98 (s, 2H, *H* ^4,4′^) 3.76 (s, 2H, C*H* ^a^), 2.55, 2.42 (s, 6H, Me^5,5′^), 2.08, 2.07 (s, 6H, Me^3,3′^), 0.95 (s, 18H, ^t^Bu). ^13^C NMR (101 MHz, CDCl_3_) δ 151.24, 150.14, (s, C^3,3′^), 141.41, 139.34, (s, C^5,5′^), 107.54, 107.17 (s, C^4,4′^), 86.57 (s, C^a^), 62.53 (s, *C*H), 35.99 (s, C-^t^Bu), 26.60 (s, *^t^* Bu), 14.07, 12.85 (s, Me^3,3′^), 11.86, 11.27 (s, Me^5,5′^). LC-MS *m*/*z* for C_32_ H_50_ Ga N_8_ O_2_: 647.3312 (100%).

#### Synthesis of [Ga­(κ^3^-bpzFerr)_2_]^+^[GaCl_4_]^−^ (**Ga2**)

Complex Ga2 was obtained following the same procedure as for complex Ga1 by reaction of bpzFerrH (1.00 g, 2.39 mmol), ^n^BuLi (1.6 M in hexanes, 1.55 mL, 2.51 mmol), and GaCl_3_ (0.44 g, 2.51 mmol) as an orange solid. Yield: 2.19 g (82%). Anal. Calcd for C_44_H_50_Cl_4_Fe_2_Ga_2_N_8_O_2_: C, 47.4; H, 4.5; N, 10.0. Found: C, 47.8; H, 4.7; N, 9.8. ^1^H NMR (400 MHz, CDCl_3_) δ 6.22 (s, 2H, C*H*), 6.05, 5.91 (s, 2H, *H* ^4,4′^), 4.77 (s, 2H, C*H* ^a^), 4.48 (s, 2H, Cp), 4.35 (s, 10H, Cp′), 4.29 (s, 2H, Cp), 4.18 (s, 2H, Cp), 4.14 (s, 2H, Cp), 2.56, 2.15 (s, 6H, Me^3,3′^), 2.05, 1.37 (s, 6H, Me^5,5′^). ^13^C NMR (101 MHz, CDCl_3_) δ 150.58, 149.18 (s, C^3,3′^), 141.79, 140.70 (s, C^5,5′^), 107.56, 106.89 (s, C^4,4′^), 91.81 (s, Cp), 75.60 (s, C^a^), 71.11 (s, *C*H), 69.05–68.88 (s, Cp, Cp′), 12.55, 11.81 (s, Me^3,3′^), 11.55, 11.00 (s, Me^5,5′^). LC-MS *m*/*z* for C_44_ H_50_ Fe_2_ Ga N_8_ O_2_: 903.2016 (100%).

#### Synthesis of [Ga­(κ^3^-bpzappe)_2_]^+^[GaCl_4_]^−^ (**Ga3**)

Complex **Ga3** was obtained following the same procedure as for complex **Ga1** by reaction of bpzappeH (1.00 g, 2.33 mmol), ^n^BuLi (1.6 M in hexanes, 1.53 mL, 2.44 mmol) and GaCl_3_ (0.43 g, 2.44 mmol) as a red solid. Yield: 2.07g (78%). Anal. Calcd for C_52_H_60_Cl_4_Ga_2_N_10_O_2_: C, 54.9; H, 5.3; N, 12.3. Found: C, 55.2; H, 5.5; N, 12.1. ^1^H NMR (500 MHz, CDCl_3_) δ 8.05, 7,83 (m, 4H, Ph), 7.86, 7.76 (d, *J*_HH_ = 7.7 Hz, 4H, Ph) 7.36 (t, *J*_HH_ = 7.7 Hz, 4H, Ph), 7.24 (s, 2H, C*H*), 7.15 (t, *J*_HH_ = 7.7 Hz, 2H, Ph), 7.02 (t, *J*_HH_ = 7.3 Hz, 2H, Ph), 6.79 (d, *J*_HH_ = 8.3 Hz, 4H, Ph), 6.58 (d, *J*_HH_ = 9 Hz, 4H, Ph), 5,93 (s, 4H, C*H* ^4,4′^) 5.60, 5.51 (s, 2H, C*H* ^4,4′^), 2.95, 2.85 (s, 12H, NMe_2_), 2.72, 2.71, 2.27, 2.26 (s, 6H, Me^3,3′^), 1.69, 1.54, 1.23, 1.21 (s, 6H, Me^5,5′^). ^13^C NMR (126 MHz, CDCl_3_) δ 150.97, 150.93, 149.77, 149.70 (s, C^3,3′^), 149.25, 149.03, 146.99, 146.27 (s, C^5,5′^), 135.16–112.29 (s, Ph), 107.93, 108.00, 106.72, 106.47 (s, C^4,4′^), 83.80 (s, C^a^), 67.68 (s, *C*H), 41.13, 40.88 (s, NMe_2_), 12.93, 12.90, 12.57, 11.59 (s, Me^3,3′^), 13.03, 12.80, 11.78, 11.75 (s, Me^5,5′^). LC-MS *m*/*z* for C_52_ H_60_ Ga N_10_ O_2_: 925.4155 (100%).

#### Synthesis of [Ga­(κ^3^-bpznaft)_2_]^+^[GaCl_4_]^−^ (**Ga4**)

Complex **Ga4** was obtained following the same procedure as for complex **Ga1** by reaction of bpznaftH (0.250 g, 0.722 mmol), *^n^* BuLi (1.6 M in hexanes, 0.48 mL, 0.758 mmol) and GaCl_3_ (0.127 g, 0.722 mmol) as a white solid. Yield: 0.48 g (67%). Anal. Calcd for C_44_H_46_Cl_4_Ga_2_N_8_O_2_: C, 52.8; H, 4.6; N, 11.2. Found: C, 53.2; H, 5.0; N, 10.9. ^1^H NMR (500 MHz, CDCl_3_) δ 8.10 (d, *J*_HH_ = 8.7 Hz, 2H, C_10_H_7_), 7.94 (d, *J*_HH_ = 7.9 Hz, 2H, C_10_H_7_), 7.74 (d, *J*_HH_ = 8.1 Hz, 4H, C_10_H_7_), 7.69 (d, *J*_HH_ = 7.1 Hz, 2H, C_10_H_7_), 7.57 (t, *J*_HH_ = 7.6 Hz, 2H, C_10_H_7_), 7.32 (t, *J*_HH_ = 8.0 Hz, 2H, C_10_H_7_), 6.71 (br, 2H, C*H*), 6.53 (br, 2H, C*H* ^a^), 6.25, 5.74 (s, 2H, *H* ^4,4′^), 2.68, 2.23 (s, 6H, Me^3,3′^), 1.41, 1.20 (s, 6H, Me^5,5′^). ^13^C NMR (126 MHz, CDCl_3_) δ 150.68, 150.32 (s, C^3,3′^), 141.76, 140.92 (s, C^5,5′^), 136.25 (s, C-*ipso*), 133.39 (s, C_5_–C_10_H_7_), 129.96, 129.35, 128.49, 127.12, 126.39, 125.62, 119.95 (s, C_10_H_7_), 125.24 (s, C_10_–C_10_H_7_), 108.45, 106.68 (s, C^4,4′^), 75.56 (s, C^a^), 65.90 (s, *C*H), 11.85, 11.38 (s, Me^3,3′^), 12.75, 9.56 (s, Me^5,5′^). LC-MS *m*/*z* for C_44_ H_46_ Ga N_8_ O_2_: 787.2996 (100%).

#### Synthesis of [Ga­(κ^3^-bpzantra)_2_]^+^[GaCl_4_]^−^ (**Ga5**)

Complex **Ga5** was obtained following the same procedure as for complex **Ga1** by reaction of bpzantraH (0.7 g, 1.70 mmol), ^n^BuLi (1.6 M in hexanes, 1.1 mL, 1.78 mmol) and GaCl_3_ (0.3 g, 1.70 mmol) as a white solid. Yield: 1.34 g (72%). Suitable crystals for X-ray analysis were obtained at −25 °C from a hexane/THF solution. Anal. Calcd for C_52_H_50_Cl_4_Ga_2_N_8_O_2_: C, 56.8; H, 4.6; N, 10.2. Found: C, 57.0; H, 4.9; N, 10.0. ^1^H NMR (500 MHz, CDCl_3_) δ 9.88 (d, *J*_HH_ = 9.4 Hz, 2H, C_14_H_9_), 8.43 (s, 2H, C_14_H_9_), 8.32 (d, *J*_HH_ = 9.1 Hz, 2H, C_14_H_9_), 8.13 (d, *J*_HH_ = 8.4 Hz, 2H, C_14_H_9_), 7.77 (d, *J*_HH_ = 8.3 Hz, 2H, C_14_H_9_), 7.72 (ddd, *J*_HH_ = 8.8, 6.6, 1.4 Hz, 2H, C_14_H_9_), 7.58 (dd, *J*_HH_ = 8.5, 6.5 Hz, 2H, C_14_H_9_), 7.32 (s, 2H, C*H*), 7.10 (ddd, *J*_HH_ = 8.2, 6.5, 1.1 Hz, 2H, C_14_H_9_), 6.81 (ddd, *J*_HH_ = 9.5, 6.4, 1.5 Hz, 2H, C_14_H_9_), 6.58 (d, *J*_HH_ = 1.2 Hz, 2H, C*H* ^a^), 6.34, 5.56 (s, 2H, H^4,4′^), 2.63, 2.32 (s, 6H, Me^3,3′^), 1.52, 0.88 (s, 6H, Me^5,5′^). ^13^C NMR (126 MHz, CDCl_3_) δ 150.87, 150.68 (s, C^3,3′^), 141.25, 140.90 (s, C^5,5′^), 132.39, 132.13 (s, C-*ipso*), 130.95, 130.88 (s, C^8,9^), 130.80, 128.86, 128.05, 127.73, 127.46, 127.22, 125.46, 125.10, 124.69, 120.99 (s, C_14_H_9_) 108.72, 107.20 (s, C^4,4′^), 79.83 (s, C^a^), 68.14 (s, *C*H), 12.88, 9.38 (s, Me^3,3′^) 12.07, 11.50 (s, Me^5,5′^). LC-MS *m*/*z* for C_52_ H_50_ Ga N_8_ O_2_: 887.330 (100%).

#### Synthesis of [Ga­(κ^3^-bpzapape)_2_]^+^[GaCl_4_]^−^ (**Ga6**)

Complex **Ga6** was obtained following the same procedure as for complex **Ga1** by reaction of bpzapapeH (0.324 g, 0.690 mmol), ^n^BuLi (1.6 M in hexanes, 0.45 mL, 0.720 mmol) and GaCl_3_ (0.121 g, 0.690 mmol) as a green solid. Yield: 0.55 g (65%). Suitable crystals for X-ray analysis were obtained at 3 °C from an CHCl_3_/hexane solution. Anal. Calcd for C_56_H_70_Cl_4_Ga_2_N_12_O_2_: C, 54.9; H, 5.8; N, 13,7. Found: C, 55.3; H, 6.1; N, 13.4. ^1^H NMR (400 MHz, CDCl_3_) δ 7.84 (d, *J*_HH_ = 8.3 Hz, 4H, ^*o*^ *H*Ph), 7.71 (d, *J*_HH_ = 8.6 Hz, 4H, ^*o*^ *H*Ph), 7.19 (s, 2H, C*H*), 6.74 (d, *J*_HH_ = 8.3 Hz, 4H, ^*m*^ *H*Ph), 6.54 (d, *J*_HH_ = 8.6 Hz, 4H, ^*m*^ *H*Ph), 5.90, 5.57 (s, 2H, C*H* ^4,4′^), 2.93 (s, 12H, NMe_2_), 2.82 (s, 12H, NMe_2_), 2.70 (s, 3H, Me^5^), 2.27 (s, 3H, Me^3′^), 1.67 (s, 3H, Me^5′^), 1.23 (s, 3H, Me^3^). ^13^C NMR (101 MHz, CDCl_3_) δ 150.76, 149.37 (s, C^3,3′^), 141.99, 140.91 (s, C^5,5′^), 135.56–129.07 (s, *C*_4_Ph-NMe_2_), 127.10, 127.01 (s, ^*o*^Ph-NMe_2_), 112.25, 112.13 (s, ^*m*^Ph-NMe_2_), 107.70 (s, C_4_), 106.51 (s, C_4′_) 83.41 (s, C^a^), 68.10 (s, C*H*) 40.96, 40.77 (s, NMe_2_) 13.04 (s, Me^5′^), 12.51 (s, Me^5^) 11.71­(s, Me^3^), 11.56 (s, Me^3′^). LC-MS *m*/*z* for C_56_ H_70_ Ga N_12_ O_2_: 1011.499 (100%).

#### X-ray Crystallographic Structure Determination

Single crystal X-ray diffraction (SCXRD) data were collected on a Bruker D8 Venture diffractometer equipped with Mo Kα radiation (λ = 0.71073) and a Photon3 detector. Suitable single crystals of **Ga5** and **Ga6** were mounted on MyTeGen polymer loops and data were acquired at room temperature. The data collection and reduction were performed using APEX4 software and the absorption correction was performed using SADABS. Crystalline structures were solved and refined using SHELX software package^84,85^ integrated in OLEX2 ^86^ as graphical interface (Tables S1–S6). All non-H atoms were refined anisotropically with exception of the solvent molecules. H atoms were placed in idealized calculated positions and refined with idealized geometries. For **Ga6** compound, one of the aromatic rings of the ligand was found to be disordered and thus modeled in concordance. Crystallographic data (excluding structure factors) have been deposited with the Cambridge Crystallographic Data Centre as supplementary publication (CCDC reference numbers 2498204 and 2498203 for **Ga5** and **Ga6**, respectively). Copies of the data can be obtained free of charge on application to the Director, CCDC, 12 Union Road, Cambridge, CB2 1EZ, U.K. (Fax: + 44–1223–335033; e-mail: deposit@ccdc.cam.ac.uk).

#### Analytical Purity Determination by HPLC and LC–MS

The analytical purity of all gallium complexes (**Ga1–Ga6**) used in biological assays was determined by analytical HPLC-DAD using peak-area normalization. Analyses were performed on an Agilent 1200 series HPLC system equipped with a G1311A binary pump, a G1329A autosampler, a G1322A degasser and a G1316A column oven. The DAD detector was operated in both scan mode and at fixed wavelengths of 210, 254, and 280 nm. Separation was achieved using an Infinity Lab Poroshell 120 SB-C18 column (75 mm length x 3 mm internal diameter; 2.7 μm particle size) from Agilent Technologies that was maintained at 25 °C. The mobile phase consisted of water and acetonitrile, using an isocratic method of 90% acetonitrile over 5 min, at a flow rate of 0.450 mL·min^–1^. Injection volume was 2 μL. In all cases, chromatographic purity exceeded 95% based on peak percentage area. The exact batches employed in the biological studies were subjected to purity analysis before use.

Mass spectrometry analyses were performed on a 6546 LC/Q-TOF mass spectrometer from Agilent Technologies, equipped with an atmospheric pressure electrospray ionization (ESI) interface operating in positive mode. The spectra show the expected molecular ion corresponding to the intact cationic gallium complex together with characteristic ligand-related fragmentation patterns. Minor signals observed at higher *m*/*z* values are attributed to ionization-induced species formed during the electrospray process. When identifiable solvent or sodium adducts were detected, their relative abundance was minimal and did not affect the overall purity assessment.

HPLC chromatograms and corresponding mass spectra are provided in the Supporting Information.

#### Stability Studies

5 mg of each gallium compound was dissolved in a mixture of DMSO-*d*_6_:D_2_O (1:1), and the stability over time was evaluated by NMR spectroscopy.

#### Lipophilicity Studies: Determination of log *P* Values

For the calibration curve, 1 mg of each gallium compound was dissolved in the proper octanol volume. Then, 2 mg of each compound were dissolved in octanol, mixed with the equal volume of water and stirred. The biphasic solution was left without stirring for one night. Then, the concentration of the corresponding compound in the octanol phase was determined via UV–vis absorption spectroscopy.

### Preclinical Evaluation In Vitro

The compounds were dissolved in dimethyl sulfoxide (DMSO) before performing each experiment. The maximal concentration used was 20 μM, due to limited water solubility. The same volume of solvent was added to control conditions and did not exceed 0.5% v/v.

#### Cell Culture Studies

The cell lines MCF7, SKBR3, BT549, and MDA-MB-231, the immortalized nontransformed keratinocyte cell line HaCaT, and the human mammary epithelial cell line MCF10A were acquired from ATCC. MCF7, SKBR3, MDA-MB-231, and HaCaT cell lines were grown in DMEM, and BT549 cell line was grown in RPMI. Both mediums were supplemented with 10% fetal bovine serum (FBS) and 1% penicillin/streptomycin. MCF10A cell line was grown with DMEM F12, containing 5% horse serum, 0.1% insulin, 0.05% hydro and 0.02% human Epidermal Growth Factor (hEGF). All the cells were incubated at 37 °C and 5% CO2. Cisplatin, oxaliplatin and carboplatin were purchased from BLDpharm.

#### MTT Metabolization Assays

For viability assessment of the ligands and Ga1–Ga6, cell proliferation was assayed by MTT (3-(4, 5-dimethylthiazol-2-yl)-2, 5 diphenyltetrazolium bromide) (Sigma-Aldrich). Cell lines were plated at 2.000, 3000, 5000, and 7500 cells per well in 96-multiwell plates for BT549 and MDA-MB-231; HaCaT; SKBR3 and MCF7; and MCF10A, respectively. Twenty-four h later, the cells were treated with increasing doses of each compound for 72 h. After that, 20 μL of MTT 1.5 mg/mL was added to each well for a final concentration of 0.25 mg/mL, and plates were incubated for 45–60 min in growth conditions. The medium was removed, and MTT crystals were solubilized with 0.1 mL of dimethyl sulfoxide (DMSO) (Sigma-Aldrich). Absorbance (Abs_570nm_ – Abs_690nm_) was recorded in a spectrophotometer multiwell plate reader (MultiskanTM GO Microplate Spectrophotometer (Thermo Scientific, Finland)).

#### Matrigel 3D Assay

Breast cancer cell lines MDA-MB-231 and MCF7 (tumoral) and MCF10A (nontumoral) (5,000 or 10,000 cells) were seeded on a layer (1 mm) of Matrigel (48-well plates). After 24 h of incubation, cells were treated for 72 h with **Ga3** and **Ga6** compounds and cisplatin as a control. An inverted microscope was used to monitor the morphology of invading cultures, and ImageJ software was used to quantify the diameter of 3D structures.

#### Generation of ROS Species

The fluorescence intensity related to the generation of ROS was measured with a FLUOstar OPTIMA microplate reader. 2′,7′-dichlorodihydrofluorescein diacetate (H2DCFDA) from Sigma-Aldrich was used as the ROS indicator. MDA-MB-231 and MCF7 cell lines were plated at 2000 cells per well in 96-well plates using DMEM without phenol red and supplemented with 10% fetal bovine serum (FBS) and 1% penicillin/streptomycin and were incubated for 24 h at 37 °C and 5% of CO_2_. After that, the medium was aspirated, and the cells were treated at the IC_50_ value of each gallium complex and cisplatin and at 8 μM of H_2_O_2_ as the positive control. 24h later, 20 μL of H2DCFDA 90 μM in DMEM without phenol red were added to each well to obtain a final concentration of 15 μM. The plate was incubated for 30 min, and the fluorescence intensity was recorded at 520 nm.

#### Intracellular Fe­(II) Detection with Mito-FerroGreen

MDA-MB-231 cells were seeded in a black 24-multiwell plate with a glass coverslip bottom with excellent optics (100,000 cells) and cultured at standard conditions for 24 h. Then, cells were treated with **Ga3** and **G6** for 6 h. Ammonium iron­(II) sulfate hexahydrate (Sigma-Aldrich) was used as a positive control at a final concentration of 10 μM for 1 h. Cells were washed with PBS (3x) and incubated with Mito-FerroGreen solution (MedchemExpress) (5 μM in DMEM serum-free and w/o phenol red) (30 min, at 37 °C with 5% of CO_2_ in darkness). After washing by PBS (3 x), cells were imaged by ZEISS LSM710 Confocal Laser Scanning Microscope.

#### Intracellular GSH/GSSG Detection

MDA-MB-231 cell lines were seeded in 6-multiwell plates (100,000 cells) after 24 h the cells were treated by **Ga3** and **Ga6** compounds for 24 h. Erastin at 4 μM was used as positive control. The cells were collected using trypsin-EDTA 0.25% (Sigma-Aldrich) and counted under microscopy. Total GSH/GSSG and GSH were detected using GSH/GSSG assay kit (MedchemExpress) following the manufacturer’s protocol.

#### Cell Cycle and Apoptosis Analysis

For cell cycle evaluation, MDA-MB-231 and MCF7 cell lines were plated at 100.000 cells per well in 6 multiwell plates and were incubated for 24h at 37 °C and 5% of CO_2_. After that, cells were treated at 0.5 and 1 μM for gallium complexes and 1 μM for cisplatin. 24h later, cells were fixed by incubation with 70% ethanol for 30 min. Then, the cells were washed in 2% BSA in PBS and centrifuged at 2000 rpm for 5 min. The pellets were resuspended and stained with Propidium iodide/RNase staining solution for 1 h under dark conditions (Immunostep S.L., Salamanca, Spain).

For apoptosis analysis, MDA-MB-231 and MCF7 cell lines were plated at 50.000 cells per well in 6 multiwell plates and, after 24h of incubation time, were treated for 72h at the same concentrations as for the cell cycle analysis. Then, adherent and floating cells were centrifuged, and the pellets were incubated in annexin V binding buffer (Immunostep S.L.) for 1 h in the dark with annexin V and PI staining solution (Immunostep S.L). The percentage of dead cells was determined considering early apoptotic (annexin V-positive, PI-negative), late apoptotic (annexin V-positive and PI-positive) and residual necrotic (annexin V-negative, PI-positive) cells, which were included as dead cells in the analysis.

For caspase-dependence analysis, MDA-MB-231 cells were pretreated with pan-capase inhibitor Q-VD-OPh (QVD) (Sigma-Aldrich) at a final concentration of 10 μM, 45 min before drug exposure. The apoptosis assay was then performed as detailed before.

Flow cytometry assays were evaluated in a MACSQuant Analyzer 16 flow cytometer (Miltenyi Biotec). Histogram and dot-plot representations were performed using the MACSQuantify Software 2.13.

### Preclinical Evaluation In Vivo

#### Zebrafish Maintenance

Wild-type AB zebrafish (Danio rerio) were maintained at 28 °C with a 10 h dark cycle and were fed a standard diet according to established protocols.^87^ Zebrafish embryos were raised at 28 °C in egg water (60 mg/L sea salts, Instant Ocean).^87^ When necessary, larvae were anesthetized with 0.02% tricaine methanesulfonate (#886–86–2, MS222, Sigma-Aldrich, St. Louis, MO, USA) and immobilized in 2% methylcellulose solution for analysis and photography on a Nikon SMZ18 microscope and a DS-Ri2 camera. All animal care and experiments adhered to the guidelines set by the Institutional Animal Research Committee of the University of Castilla-La Mancha and were approved under the reference number PR-2023–08 and PR-2023–27.

#### Toxicological Analysis in Zebrafish

Experimental design for toxicological analysis followed OECD Fish Embryo Acute Toxicity Test guidelines (Test No. 236, OECD) and was based on the exposition of newly fertilized zebrafish embryos for 96 h with increasing (0–8 μM) concentrations of **Ga6** dissolved in 0.5% v/v dimethyl sulfoxide (DMSO)/egg water. Fertilized embryos (4 hpf) were randomly distributed in seven 24-well plates with one embryo each well and containing 2 mL of the corresponding solution (control, solvent control, 0.5, 1, 2, 4, and 8 μM of **Ga6**). Four embryos were included on each plate as additional internal solvent controls. For each experiment 168 embryos were used (*n*_total_ = 504 embryos). Embryos were grown either at 28 or 34 °C. Toxicity evaluation was also carried out following the OECD guidelines. Apical observations every 24h from 4hpf to 96hpf using a Fluorescence stereomicroscope SMZ18 (Nikon) equipped with DS-RI2 camera (Nikon) were done to detect coagulation of embryos, lack of somite formation, nondetachment of the tail or lack of heartbeat.

#### Zebrafish Xenografts

The MDA-MB-231 cell line expressing GFP was used to develop the xenograft model. The cells were acquired from Innoprot and were grown in DMEM, supplemented with 10% fetal bovine serum (FBS) and 1% penicillin/streptomycin. Cells were incubated at 37 °C and 5% CO_2_. On the day of microinjection, cells were detached from the culture plates and 10 million cells were resuspended in 100 uL of 2% PVP (poly­(vinylpyrrolidone), SigmaAldrich) in PBS.^88^

For the xenograft experiment, the embryos were grown at 28 °C up to 48hpf. At this age, embryos were dechorionated with 2 mg/mL Pronase solution and anesthetized with tricaine. The cells suspension was microinjected (Femtojet, Eppendorf) in the yolk of 48hpf zebrafish embryos. The xenografted embryos were incubated at 34 °C during 24h. After this time, the 72 hpf embryos with the most homogeneous tumors were selected and randomly distributed into three 24-well plates with one embryo each well and containing 2 mL of the corresponding solution (control, 0.6, and 0.9 μM of **Ga6**). For each experiment 72 embryos were used (*n*_total_ = 216 embryos). The tumor growth was evaluated after 24 h post treatment (hpt) of incubation at 34 °C. The fluorescence of each tumor at initial (0 hpt) and final time (24 hpt) was recorded using a Fluorescence stereomicroscope SMZ18 (Nikon) equipped with DS-RI2 camera (Nikon). QuantiFish 2.1 software was used to quantify tumor growth based on fluorescence increment.

#### Toxicological Analysis in Nontumor-Bearing Mice

For the in vivo toxicity study, nine 7–8-week-old female BALB/c nu/nu mice were randomly divided into three groups (*n* = 3 per group): control, 2.5 mg/kg, and 5 mg/kg treatment groups. Body weight was monitored four times per week to assess potential weight changes across all groups. Mice were treated once weekly via intraperitoneal injection at a dose of 2.5 mg/kg, and 5 mg/kg of Ga6. (2-Hydroxypropyl)-β-cyclodextrin (10% in Milli-Q water) served as the vehicle for **Ga6** administration. The control group received intraperitoneal injections of the vehicle alone, administered once weekly. All animals were humanely euthanized via CO_2_ inhalation in an appropriate chamber. The study was approved by the Animal Experimentation Ethics Committee (PR-2024–06) and was conducted under the supervision of this Ethics Committee and the veterinary staff of the University of Castilla-La Mancha. Animal handling and monitoring were performed in accordance with applicable institutional and national regulations.

### Uptake Studies

#### Spectral Equipment and Measurements

Steady-state fluorescence (SSF) spectra were recorded on an FLS920 spectrofluorometer (Edinburgh Instruments) equipped with an MCPPMT (microchannel plate-photomultiplier tube) detector (R3809 model) and a TCSPC (time-correlated single photon counting) data acquisition card (TCC900 model). A Xe lamp of 450 W was used as the light source for SSF spectra, and a subnanosecond pulsed Light-Emitting Diode, EPLED-360 (Edinburgh Photonics), was employed as a light source for Time-resolved fluorescence decays (TRF). A TLC 50 temperature-controlled cuvette holder (Quantum Northwest) was used to keep the temperature at 25 °C during the spectra acquisition. Excitation and emission slits were both fixed at 4 nm, the step 1 nm, and the dwell time was 0.1 s. The excitation wavelength (λ_ex_) was 368 nm, the emission wavelength (λ_em_) was 520 nm and the Δλ_em_ was 10 nm. All measurements were performed using a 10 mm quartz cuvette (Hellma Analytics).

The fluorescence intensity decay, *I*(*t*), was fitted to the following multiexponential function using an iterative least-squares fit method.

1 $$I\left(t\right)=\sum_{i=1}^{n}\alpha_{i}\exp\left(-t/\tau i\right)$$

where α*_i_* and τ*_i_* are the amplitude and lifetime for each *i*th term. The mean lifetime of the decay was then calculated as

2 $$\tau_{m}=\frac{\sum_{i=1}^{n}\alpha_{i}\tau_{i}^{2}}{\sum_{i=1}^{n}\alpha_{i}\tau_{i}}$$

#### Cell Cultures for FLIM Microscopy

Luminal A T-47D breast cancer cell line was obtained from the American Type Culture Collection (ATCC HTB-133) and maintained in DMEM medium with phenol red, supplemented with 10% fetal bovine serum (FBS) and 1% penicillin/streptomycin. Cells were cultured at 37 °C in a 5% CO_2_ atmosphere and seeded onto 20 mm glass coverslips in 6-well plates. Once the cells reached 50–80% confluence, they were incubated with Gallium compound or with the ligand (50 nM) in serum-free medium for various incubation times, ranging from 1 to 24 h at 37 °C. After incubation, the cells were washed three times with PBS.

#### Fluorescence Lifetime Imaging (FLIM)

FLIM imaging was performed using a MicroTime 200 confocal microscope (PicoQuant). Excitation was achieved using a pulsed diode laser at 375 nm. Each FLIM image covered an 80 × 80 μm^2^ area with a pixel resolution of 520 nm and a dwell time of 2 ms. SymphoTime64 software was used to process the lifetime distribution data, and Gaussian fitting was applied to determine the peak fluorescence lifetime for each sample. A minimum of three images per sample were taken.

#### Statistical Analysis

The in vitro and in vivo experiment data are the average of three independent experiments performed in triplicate, with error bars showing the standard deviation of the triplicates. To determine if there are statistical differences, a Student’s *t* test was performed for the in vitro assays, and an ANOVA Dunnett’s multiple comparison test was conducted for the in vivo assays. The values for the statistical analyses are *, *p* ≤ 0.05; **, *p* ≤ 0.01; ***, *p* ≤ 0.001.

## Supplementary Material





## Data Availability

The data that support the findings of this study are available on request from the corresponding author.

## Notes

Procedures for catalytic intramolecular hydroalkoxylation, spectra of the ^1^H and ^13^C­{^1^H}-NMR, UV–vis chromatograms, mass spectra and complete elemental analysis for complexes **Ga1–Ga6**, kinetic measurements, and representative kinetic plots and X-ray crystallographic data for compounds **Ga5** and **Ga6** (major isomer). For the preclinical in vitro evaluation, dose–response curves, cell cycle, and apoptosis analysis are included. For the in vivo studies, the dose–response effect of **Ga6** in zebrafish embryos at 34 °C (PDF)
Molecular Formula Strings: SMILES formula of each gallium complex and the associated biochemical and biological data (CSV)

## Abbreviations Used

ESI: electrospray ionization
FLIM: fluorescence lifetime imaging microscopy
GaM: gallium maltolate
hEGF: human epidermal growth factor
H_2_DCFDA: 2′,7′-dichlorodihydrofluorescein diacetate
hpf: hours postfertilization
hpt: hours post-treatment
KP46: tris­(8-quinolinolato)­gallium­(III)
log P: logarithmic octanol/water partition coefficient
MCPPMT: microchannel plate photomultiplier tube
MLCT: metal-to-ligand charge transfer
MTT: 3-(4,5-dimethylthiazol-2-yl)-2,5-diphenyltetrazolium bromide
ORTEP: Oak Ridge thermal ellipsoid plot
SD: standard deviation
SEM: standard error of the mean
SSF: steady-state fluorescence
TCSPC: time-correlated single photon counting
TNBC: triple-negative breast cancer
TRF: time-resolved fluorescence decays
