# Supplementary material for: Exploring Gallium(III) Complexes as Emerging Therapeutic Candidates for Breast Cancer
Source: J Med Chem. 2026 Apr 6;69(8):9051–70. doi: 10.1021/acs.jmedchem.5c03480 (PMC13126684; doi:10.1021/acs.jmedchem.5c03480)
Supplement: Supplementary file 1 [file jm5c03480_si_001.pdf]

SUPPORTING INFORMATION FOR:

Exploring Gallium(III) Complexes as Emerging Therapeutic Candidates for Breast Cancer

Alberto Moreno-Fernández,[a] Elena Domínguez-Jurado,[b] Marc Martínez de Sarasa Buchaca,[a] Sofía Blas,[b] María del Mar Nobljas-López,[b] Carmen Moya,[b] Fernando de Andrés,[c,d] Iván Bravo,[b] Jose-Daniel Aroca-Aguilar,[e] Jesús-José Ferre-Fernández,[e] Julio Escribano,[e] Antonio Rodríguez-Diéguez,[f] Pablo Salcedo-Abraira,[f] Santiago García-Yuste,[,a] Carlos Alonso-Moreno,[*,b] Agustín Lara-Sánchez[*,a]*

[a]Universidad de Castilla-La Mancha, Departamento de Química Inorgánica, Orgánica y Bioquímica-Centro de Innovación en Química Avanzada (ORFEO-CINQA), Facultad de Ciencias y Tecnologías Químicas, Instituto Regional de Investigación Científica Aplicada-IRICA, 13071-Ciudad Real, Spain.

[b]Universidad de Castilla-La Mancha, Departamento de Química Inorgánica, Orgánica y Bioquímica-Centro de Innovación en Química Avanzada (ORFEO-CINQA), Unidad nanoDrug, Facultad de Farmacia, 02071-Albacete, Spain.

[c]Universidad de Castilla-La Mancha, Departamento de Química Analítica y Tecnología de Alimentos, Facultad de Farmacia de Albacete, 02071 Albacete, Spain.

[d]Universidad de Castilla-La Mancha, Instituto Regional de Investigación Científica Aplicada IRICA, 13005 Ciudad Real, Spain.

[e]Universidad de Castilla-La Mancha, Genetics, Castilla-La Mancha University Medical School, 02008-Albacete, Spain.

[f]Universidad de Granada, Departamento de Química Inorgánica, Facultad de Ciencias, 18071-Granada, Spain.

Corresponding Authors' emails: Santiago.Gyuste@uclm.es, Carlos.AMoreno@uclm.es and Agustin.Lara@uclm.es.

Table of contents

Fig. S1. ^{1}H-NMR (400 MHz) spectrum of **Ga1**............S5

Fig.S2. ^{13}C-NMR (400 MHz) spectrum of **Ga1**............S5

Fig. S3. ^{1}H-NMR (400 MHz) spectrum of **Ga2**............S6

Fig. S4. ^{13}C-NMR (400 MHz) spectrum of **Ga2**............S6

Fig. S5. ^{1}H NMR (400 MHz) spectrum of **Ga3**.............S7

Fig. S6. ^{13}C-NMR (400 MHz) spectrum of **Ga3**............S7

Fig. S7. ^{1}H NMR (500 MHz) spectrum of **Ga4**.............S8

Fig. S8. ^{13}C NMR (500 MHz) spectrum of **Ga4**.............S8

Fig. S9. ^{1}H NMR (500 MHz) spectrum of **Ga5**.............S9

Fig. S10. ^{13}C NMR (500 MHz) spectrum of **Ga5**.............S9

Fig. S11. ^{1}H NMR (400 MHz) spectrum of **Ga6**.............S10

Fig. S12. ^{13}C NMR (400 MHz) spectrum of **Ga6**.............S10

Fig. S13. UV-Vis chromatogram of **Ga1** assayed by HPLC. Retention time of the peak corresponding to the complex is indicated.............S11

Fig. S14. UV-Vis chromatogram of **Ga2** assayed by HPLC. Retention time of the peak corresponding to the complex is indicated.............S11

Fig. S15. UV-Vis chromatogram of **Ga3** assayed by HPLC. Retention time of the peak corresponding to the complex is indicated.............S12

Fig. S16. UV-Vis chromatogram of **Ga4** assayed by HPLC. Retention time of the peak corresponding to the complex is indicated.............S12

Fig. S17. UV-Vis chromatogram of **Ga5** assayed by HPLC. Retention time of the peak corresponding to the complex is indicated. S13

Fig. S18. UV-Vis chromatogram of **Ga6** assayed by HPLC. Retention time of the peak corresponding to the complex is indicated. S13

Fig. S19. Mass spectrum of cationic form of **Ga1** (C_{32} H_{50} Ga N_8 O_2). Molecular structure for the peaks found corresponding to the molecular ion (M+) and of potential fragment ions observed are indicated. S14

Fig. S20. Mass spectrum of cationic form of each **Ga2** (C_{44} H_{50} Fe_2 Ga N_8 O_2). Molecular structure for the peaks found corresponding to the molecular ion (M+) and of potential fragment ions observed are indicated. S15

Fig. S21. Mass spectrum of cationic form of each **Ga3** (C_{52} H_{60} Ga N_{10} O_2). Molecular structure for the peaks found corresponding to the molecular ion (M+) and of potential fragment ions observed are indicated. S15

Fig. S22. Mass spectrum of cationic form of each **Ga4** (C_{44} H_{46} Ga N_8 O_2). Molecular structure for the peaks found corresponding to the molecular ion (M+) and of potential fragment ions observed are indicated. S16

Fig. S23. Mass spectrum of cationic form of each **Ga5** (C_{52} H_{50} Ga N_8 O_2). Molecular structure for the peaks found corresponding to the molecular ion (M+) and of potential fragment ions observed are indicated. S17

Fig. S24. Mass spectrum of cationic form of each **Ga6** (C_{56} H_{70} Ga N_{12} O_2). Molecular structure for the peaks found corresponding to the molecular ion (M+) and of potential fragment ions observed are indicated. S17

Table S1. Complete elemental analysis of each gallium compound, depicting calculated and found values S17

Table S2. Crystallograsphic data for **Ga5** and **Ga6**. S18

Table S3. Selected bond lengths for **Ga5.** S19

Table S4. Selected bond angles for **Ga5.** S19

Table S5. Selected bond lengths **Ga6.** S20

Table S6. Selected bond angles for **Ga6.** S20

Fig. S25. Stability of **Ga6** in DMSO-d_6 over 1 week. ^{1}H NMR spectra at different times. S21

Fig. S26. Stability of **Ga6** in DMSO-d_6:D_2O (1:1) over 1 week. ^{1}H NMR spectra at different times. S22

Fig. S27. Graph of the calculated lipophilicity of **Ga1-Ga6** complexes by logP. S22

Table S7. logP data for each complex. S23

Fig. S28. Dose-response curves of **Ga1-Ga6**, cisplatin, carboplatin and oxaliplatin in four breast cancer cell lines S23

Fig. S29. Representative example of diagrams of cell populations in each phase of the cell cycle after 24h of treatment of MDA-MB-231 (A) and MCF7 (B) with **Ga3** and **Ga6**. Red histogram is for the treatment and green histogram is for the control. Histograms are normalized to the area. PE-A: propidium iodide...S24

Fig. S30. Representative example of dot plot graphs from the apoptosis analysis of MDA-MB-231 (A) and MCF7 (B) after 72h of treatment with **Ga3** and **Ga6**. PE-A: propidium iodide. APC-A: annexin V. S24

Fig. S31. Apoptosis assay was performed using pan-caspase inhibitor Q-VD-OPh before treatment with gallium compounds. A) Quantification of early (AV+/PI-) and late (AV+/PI+) apoptotic cells. To determine statistically significant differences, a Student's t-test was used to compare AV+ cells between conditions. The values for the statistical analyses are * $p \leq 0.05$; ** $p \leq 0.01$. Representative example of dot plot graphs for MDA-MB-231 (B) and MCF7 (C). PE-A: propidium iodide. APC-A: annexin V. S24

Fig. S32. Dose-dependent effects of **Ga6** on zebrafish embryo survival at 34ºC. Data are represented as mean ± SEM from three independent experiments. S26

Fig. S33. Absorption (A) and emission (B) spectra of **Ga5**...S26

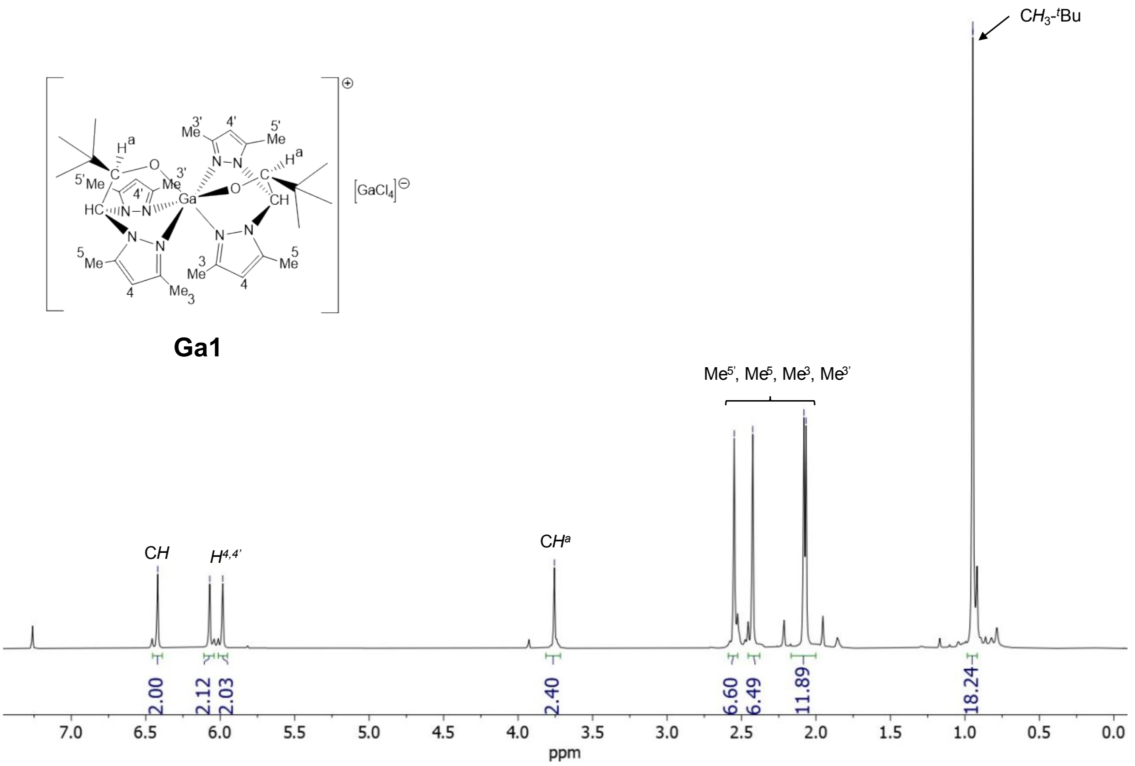

Fig. S1. ^{1}H-NMR (400 MHz) spectrum of **Ga1**.

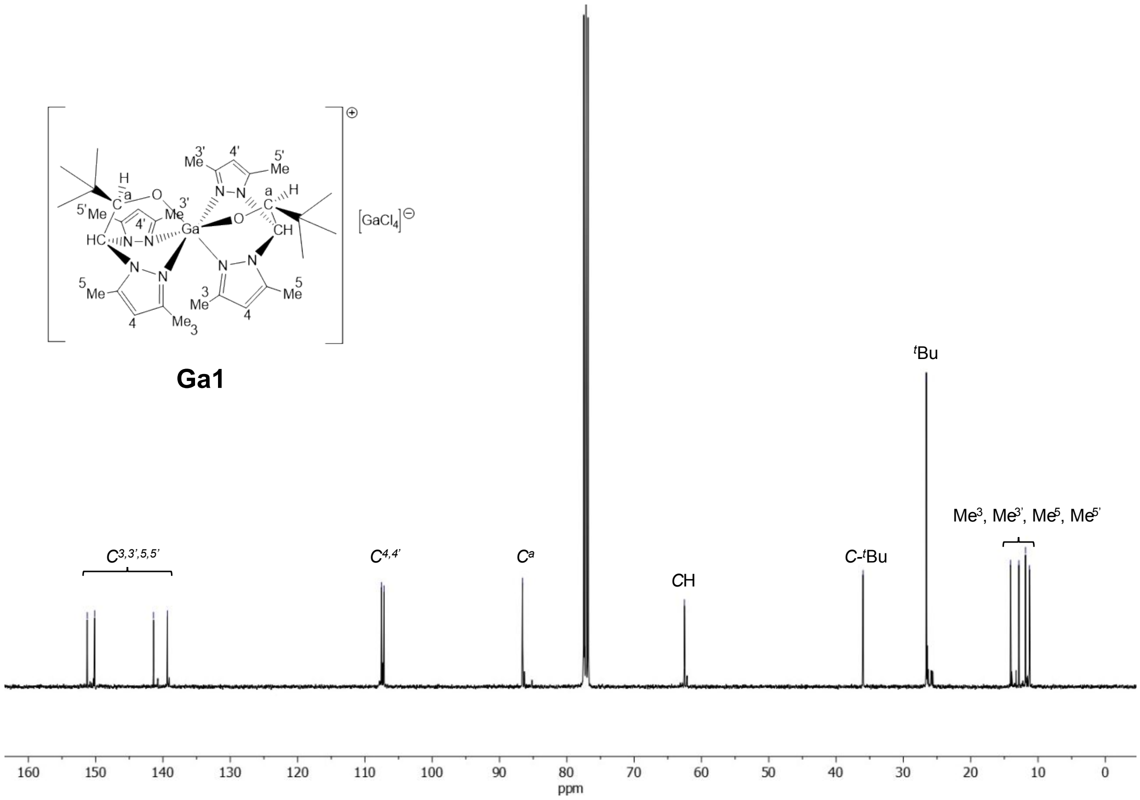

Fig.S2. ^{13}C-NMR (400 MHz) spectrum of **Ga1**.

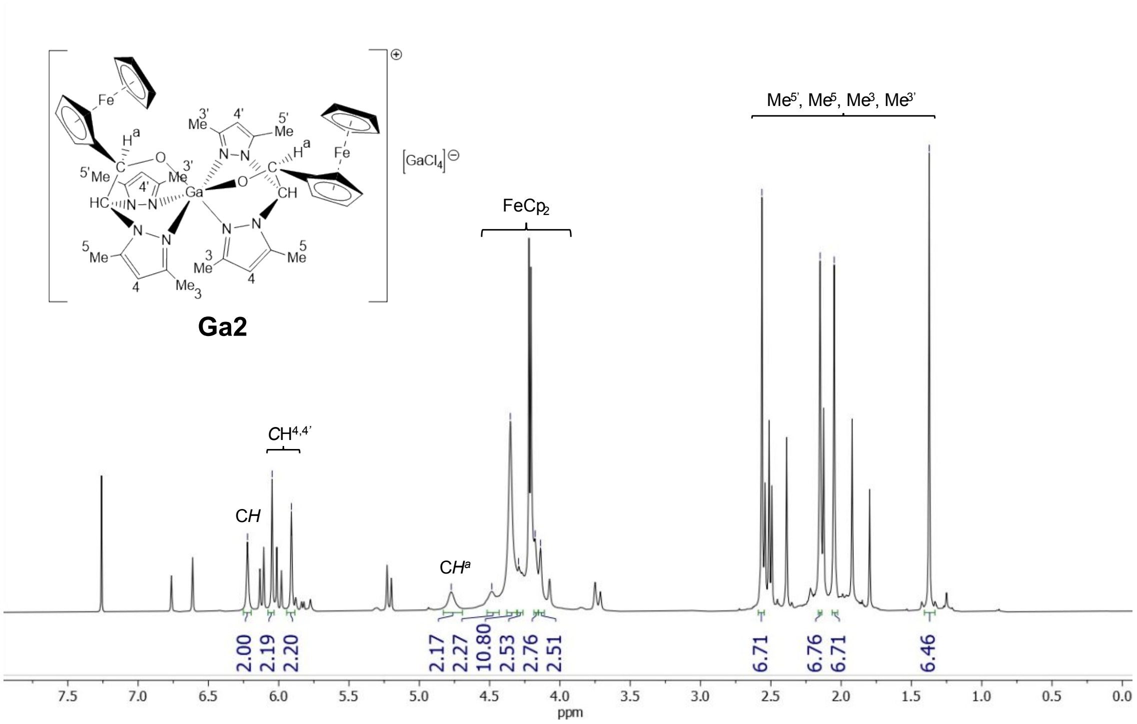

Fig. S3. ^{1}H-NMR (400 MHz) spectrum of **Ga2**.

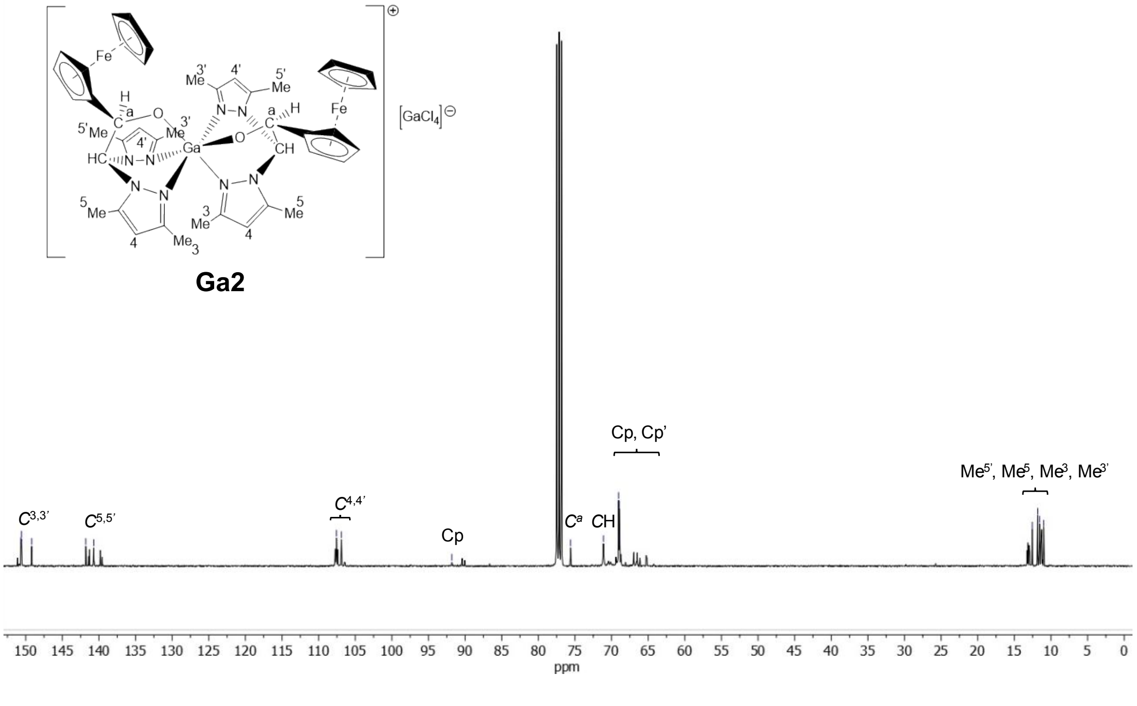

Fig. S4. ^{13}C-NMR (400 MHz) spectrum of **Ga2**.

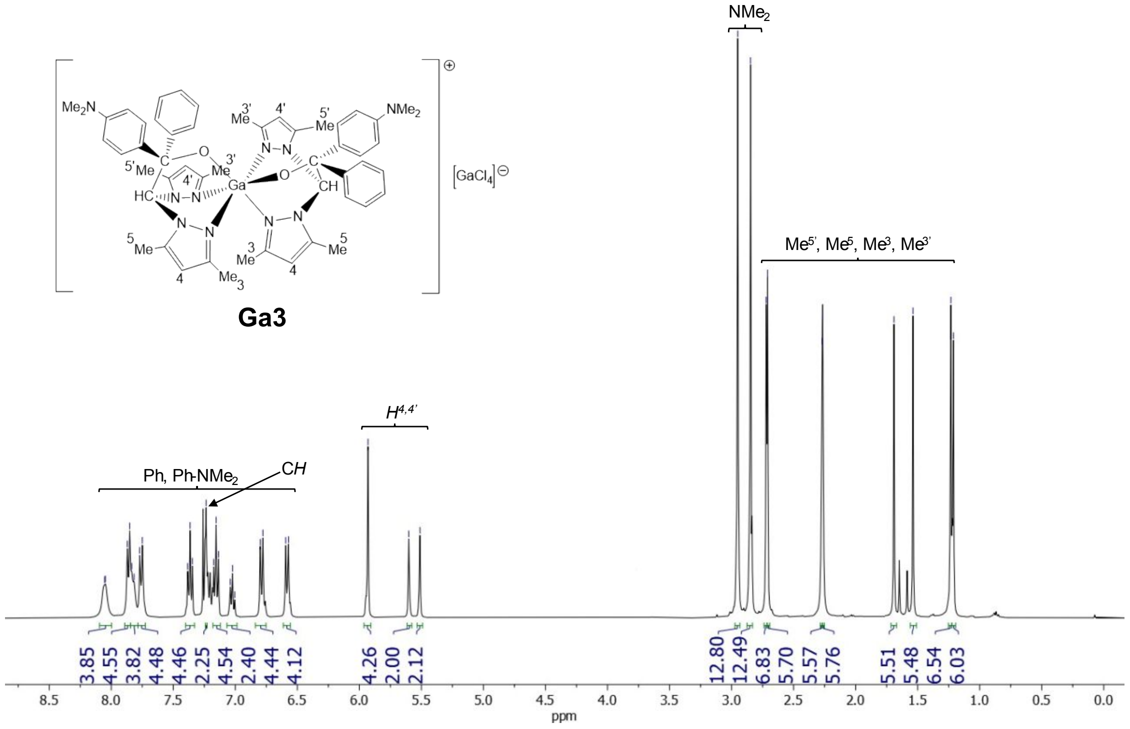

Fig. S5. ^{1}H NMR (400 MHz) spectrum of **Ga3**.

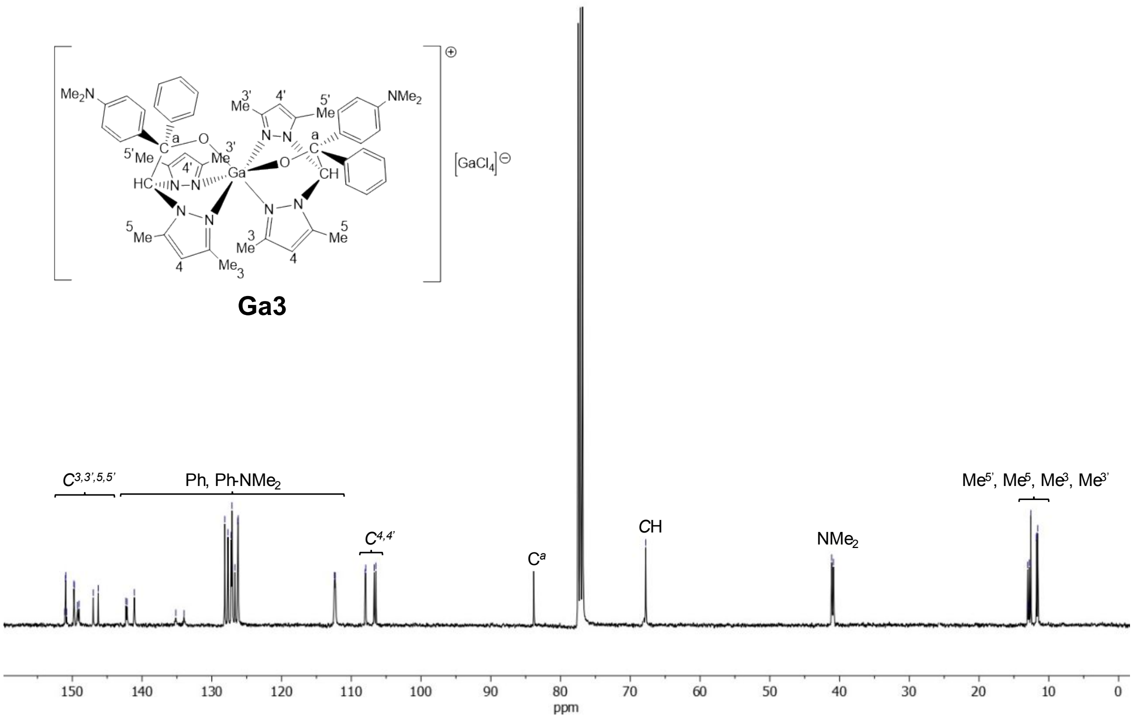

Fig. S6. ^{13}C-NMR (400 MHz) spectrum of **Ga3**.

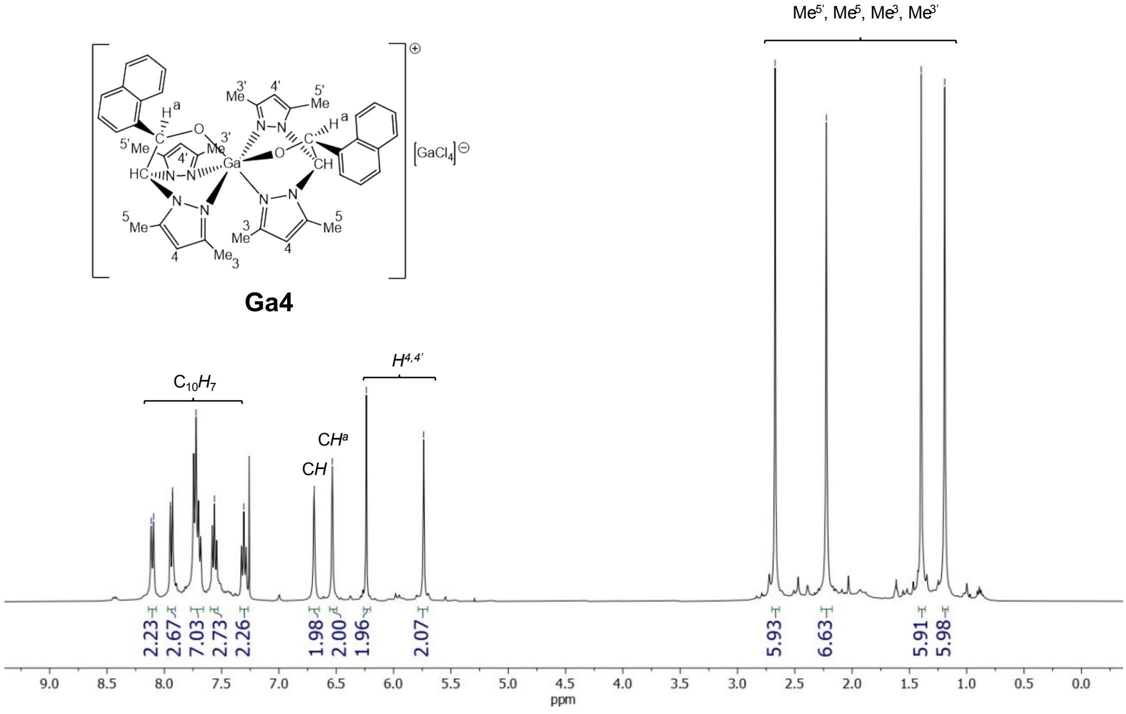

Fig. S7. ^{1}H NMR (500 MHz) spectrum of **Ga4**.

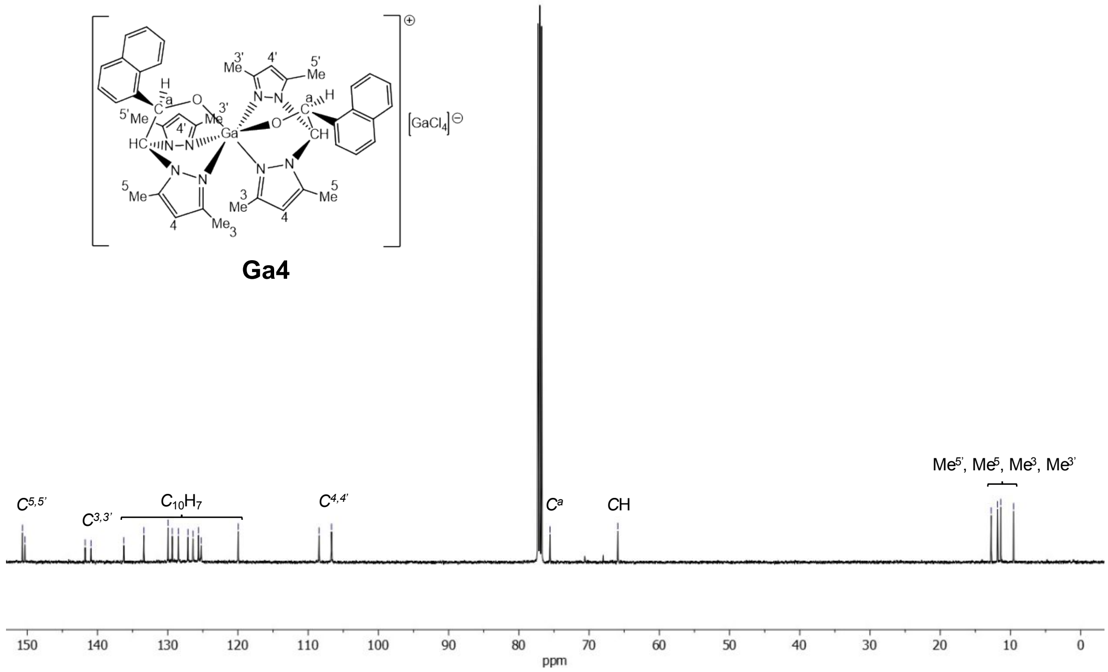

Fig. S8. ^{13}C NMR (500 MHz) spectrum of **Ga4**.

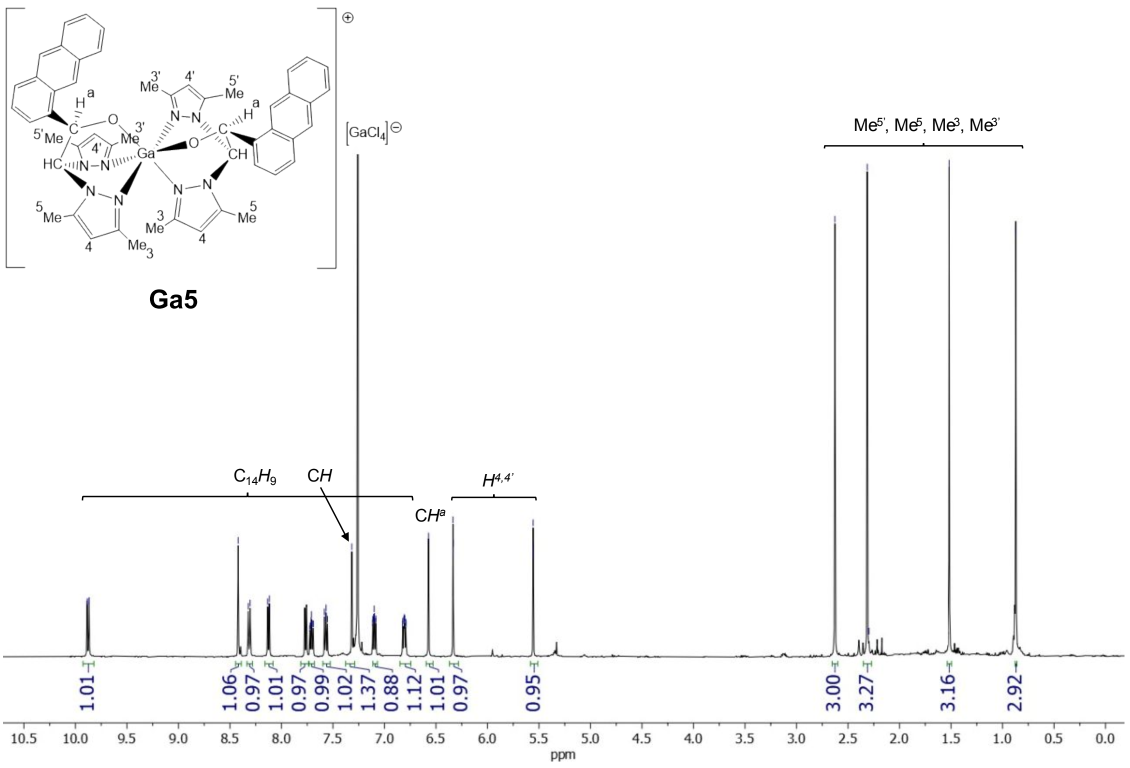

Fig. S9. ^{1}H NMR (500 MHz) spectrum of **Ga5**.

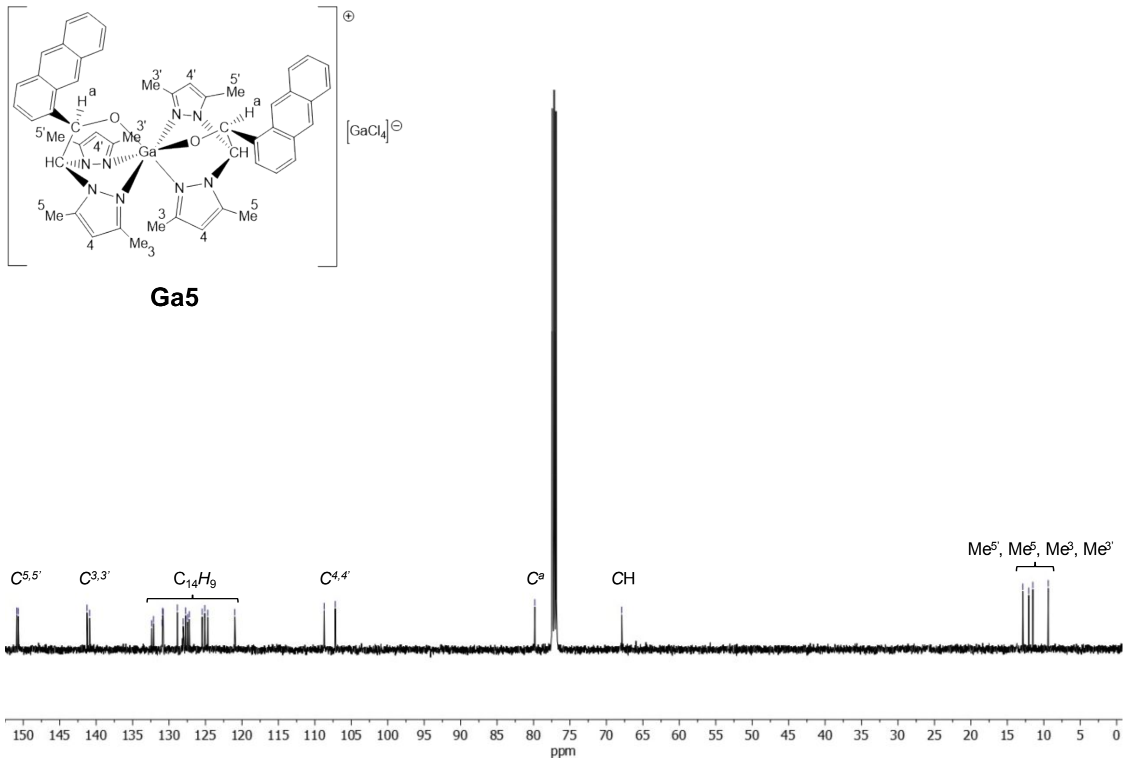

Fig. S10. ^{13}C NMR (500 MHz) spectrum of **Ga5**.

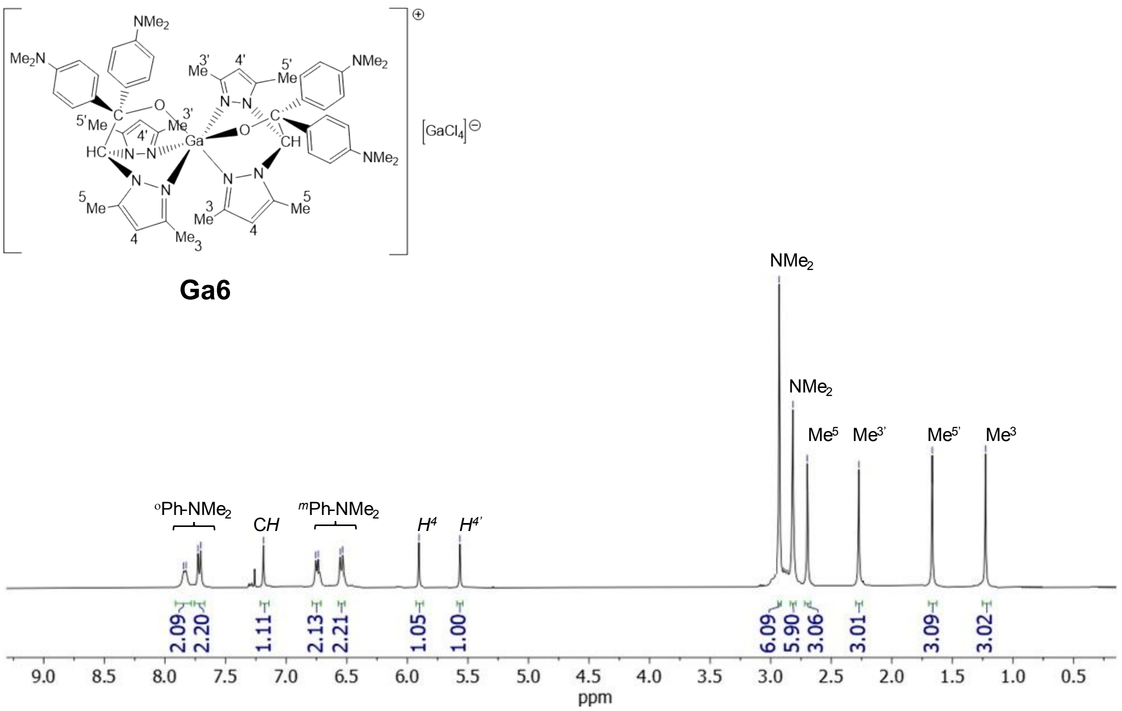

Fig. S11. ^{1}H NMR (400 MHz) spectrum of **Ga6**.

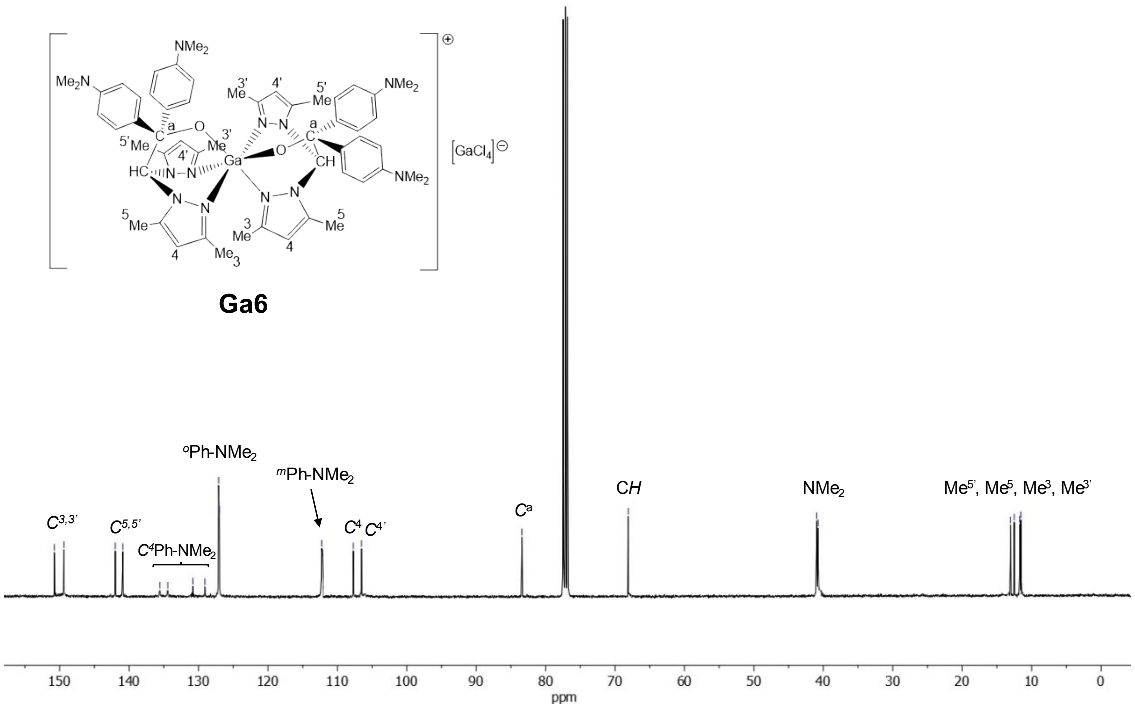

Fig. S12. ^{13}C NMR (400 MHz) spectrum of **Ga6**.

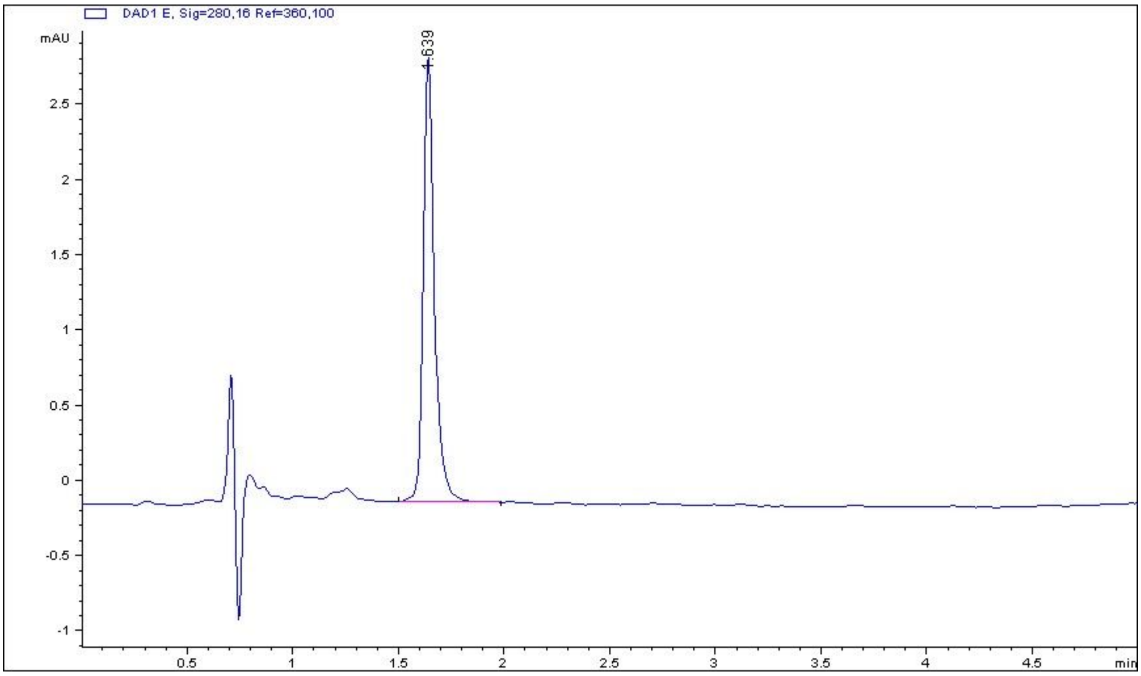

Fig. S13. UV-Vis chromatogram of **Ga1** assayed by HPLC. Retention time of the peak corresponding to the complex is indicated.

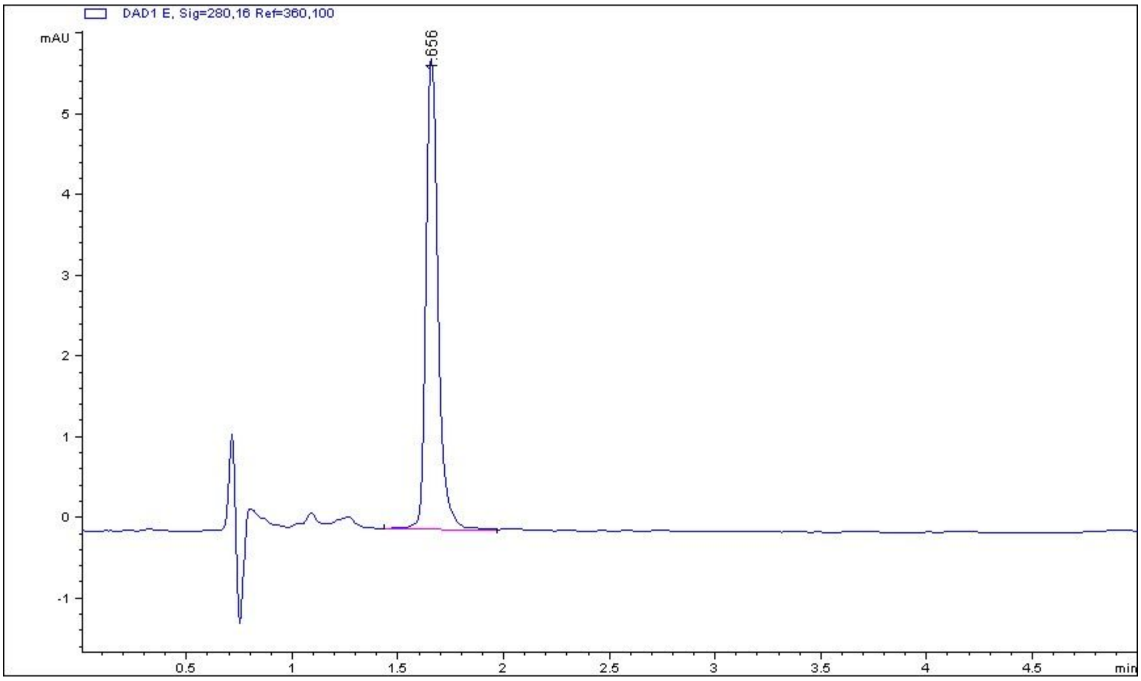

Fig. S14. UV-Vis chromatogram of **Ga2** assayed by HPLC. Retention time of the peak corresponding to the complex is indicated.

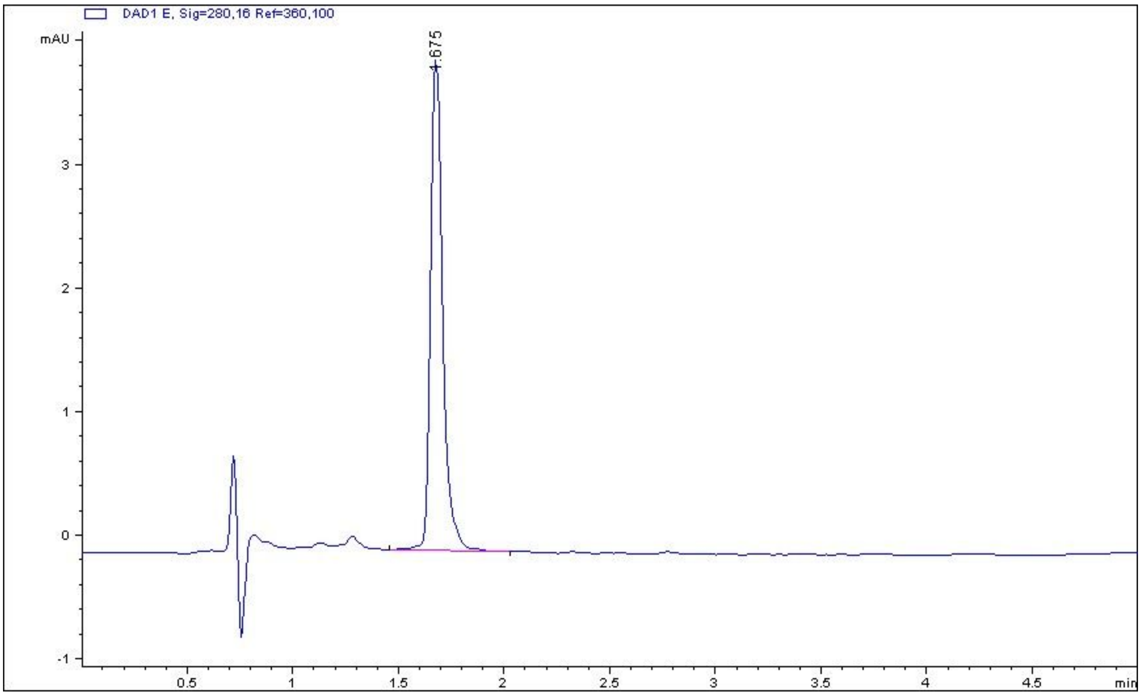

Fig. S15. UV-Vis chromatogram of **Ga3** assayed by HPLC. Retention time of the peak corresponding to the complex is indicated.

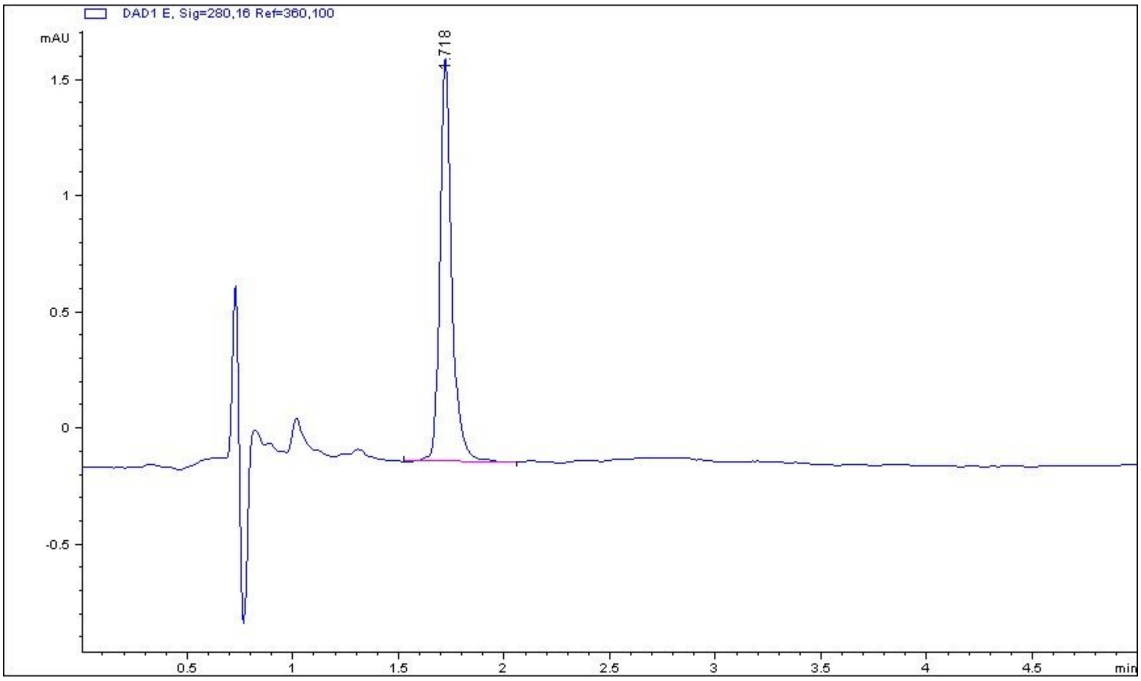

Fig. S16. UV-Vis chromatogram of **Ga4** assayed by HPLC. Retention time of the peak corresponding to the complex is indicated.

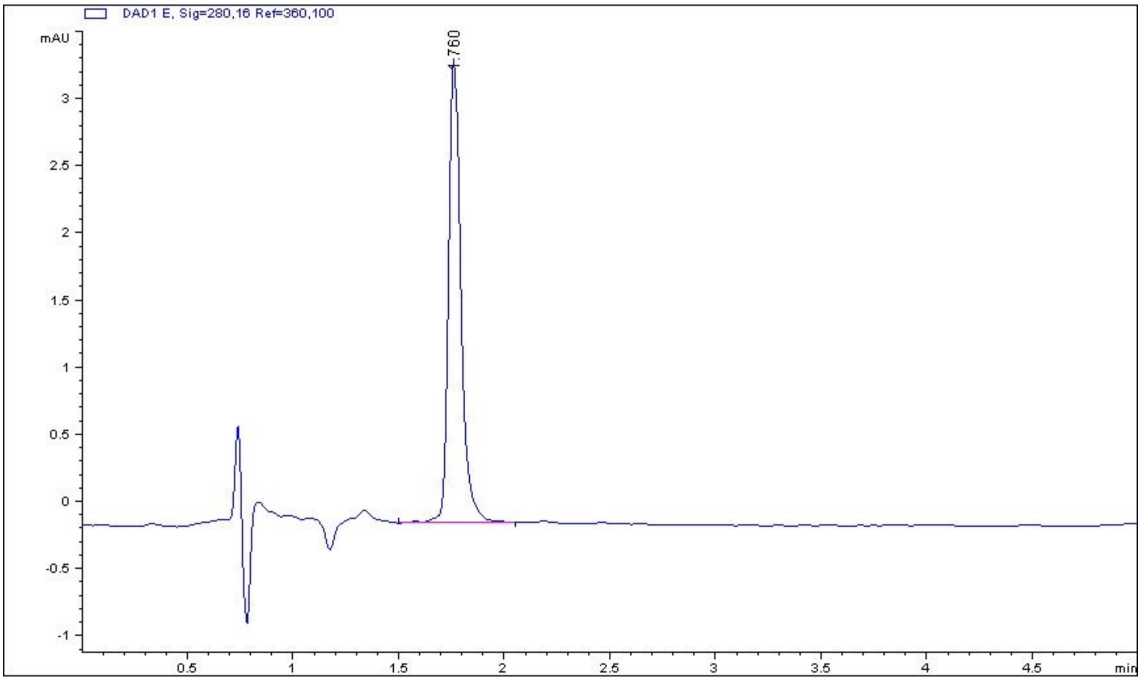

Fig. S17. UV-Vis chromatogram of **Ga5** assayed by HPLC. Retention time of the peak corresponding to the complex is indicated.

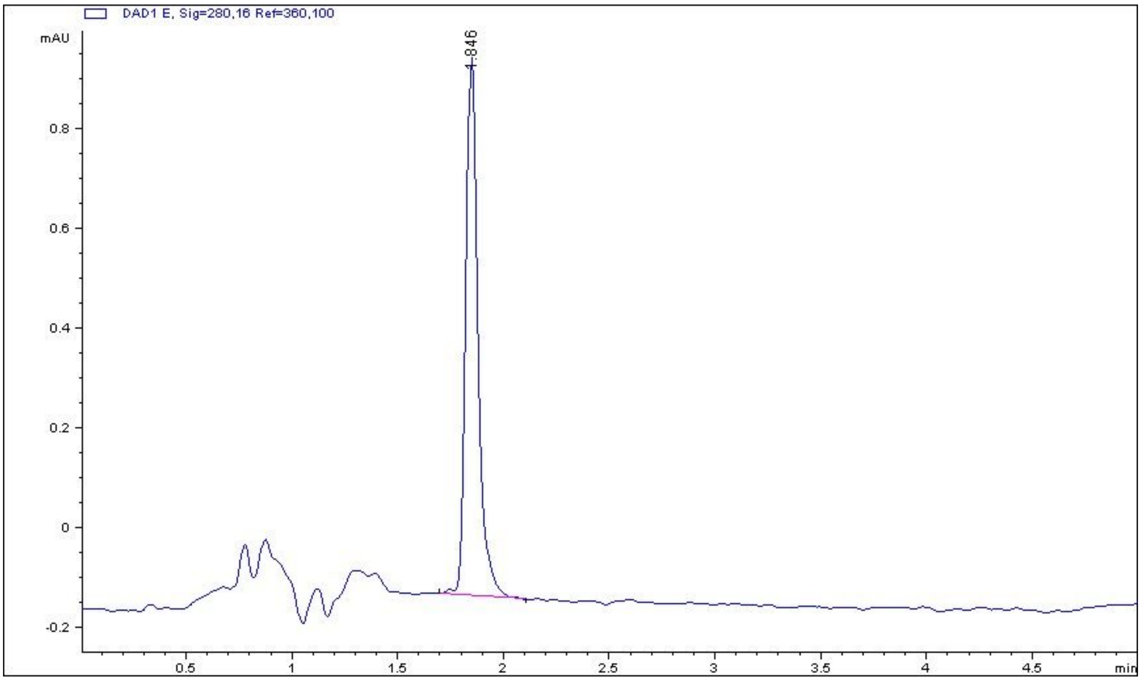

Fig. S18. UV-Vis chromatogram of **Ga6** assayed by HPLC. Retention time of the peak corresponding to the complex is indicated.

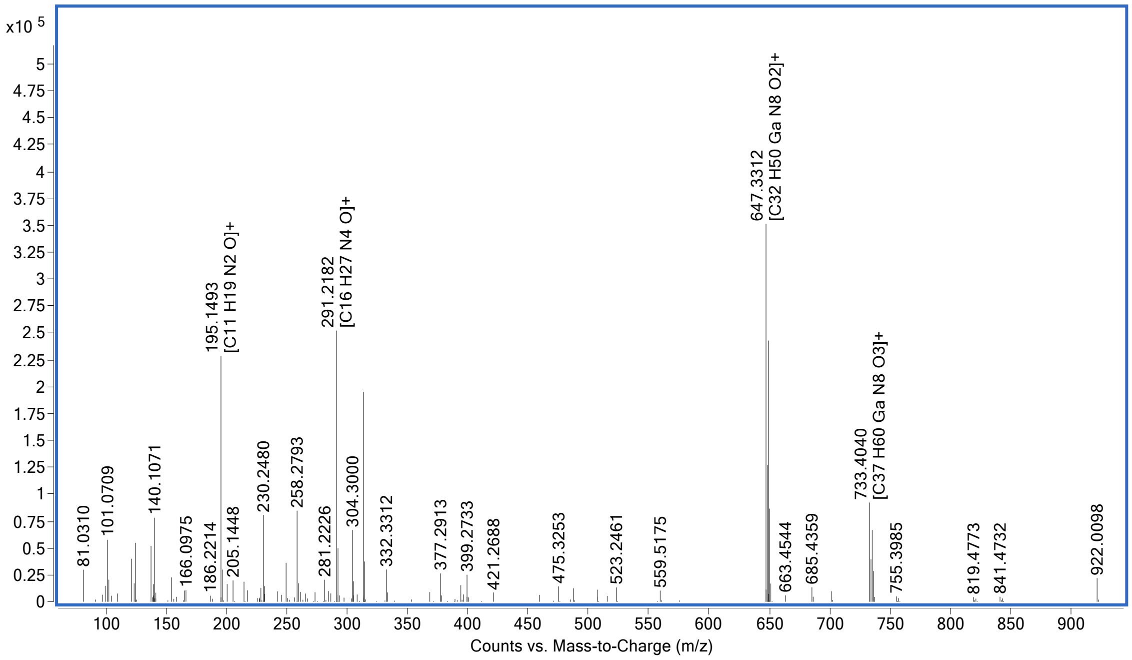

Fig. S19. Mass spectrum of cationic form of **Ga1** ($C_{32} H_{50} Ga N_8 O_2$). Molecular structure for the peaks found corresponding to the molecular ion (M+) and of potential fragment ions observed are indicated.

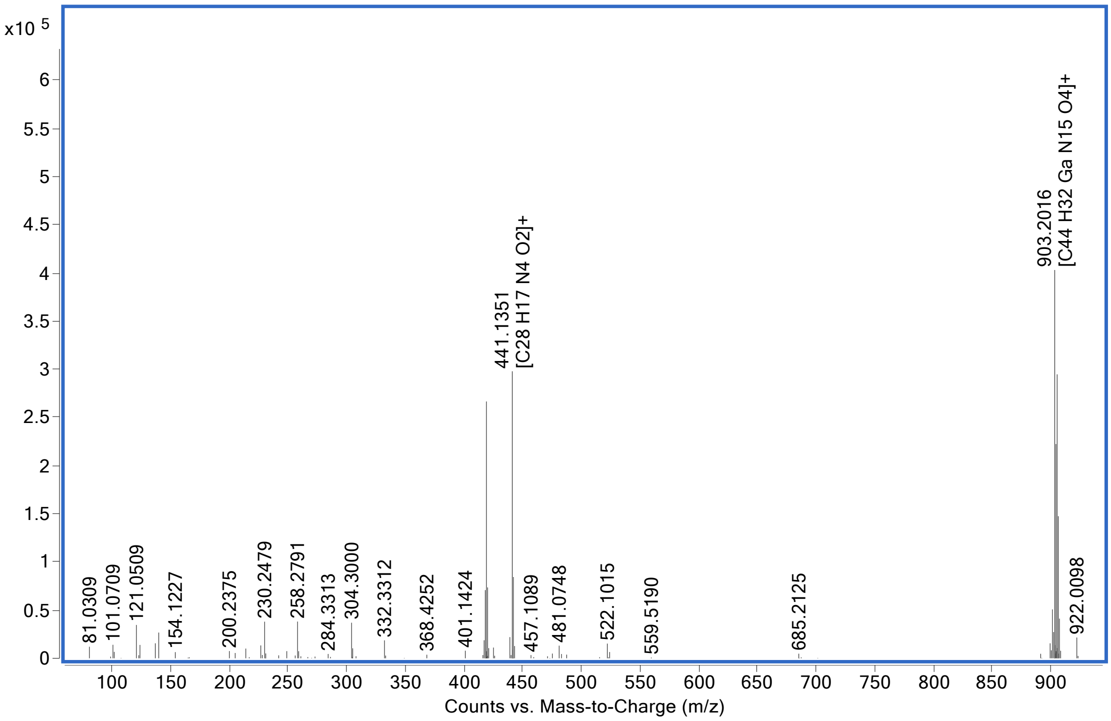

Fig. S20. Mass spectrum of cationic form of each **Ga2** (C_{44} H_{50} Fe_2 Ga N_8 O_2). Molecular structure for the peaks found corresponding to the molecular ion (M+) and of potential fragment ions observed are indicated.

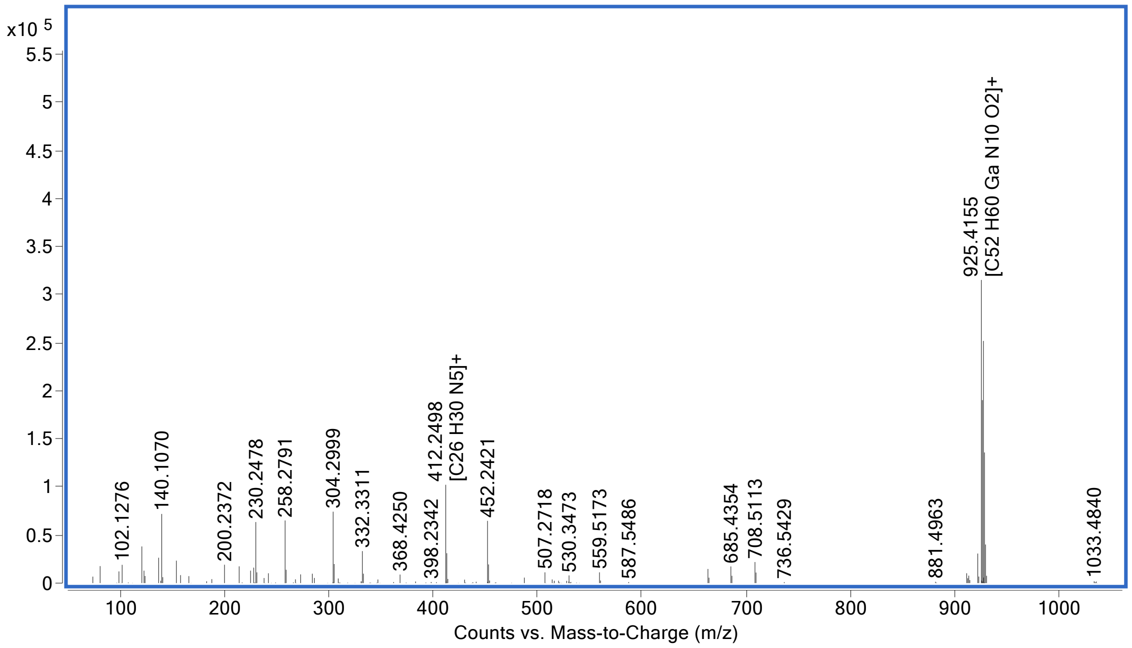

Fig. S21. Mass spectrum of cationic form of each **Ga3** (C_{52} H_{60} Ga N_{10} O_2). Molecular structure for the peaks found corresponding to the molecular ion (M+) and of potential fragment ions observed are indicated.

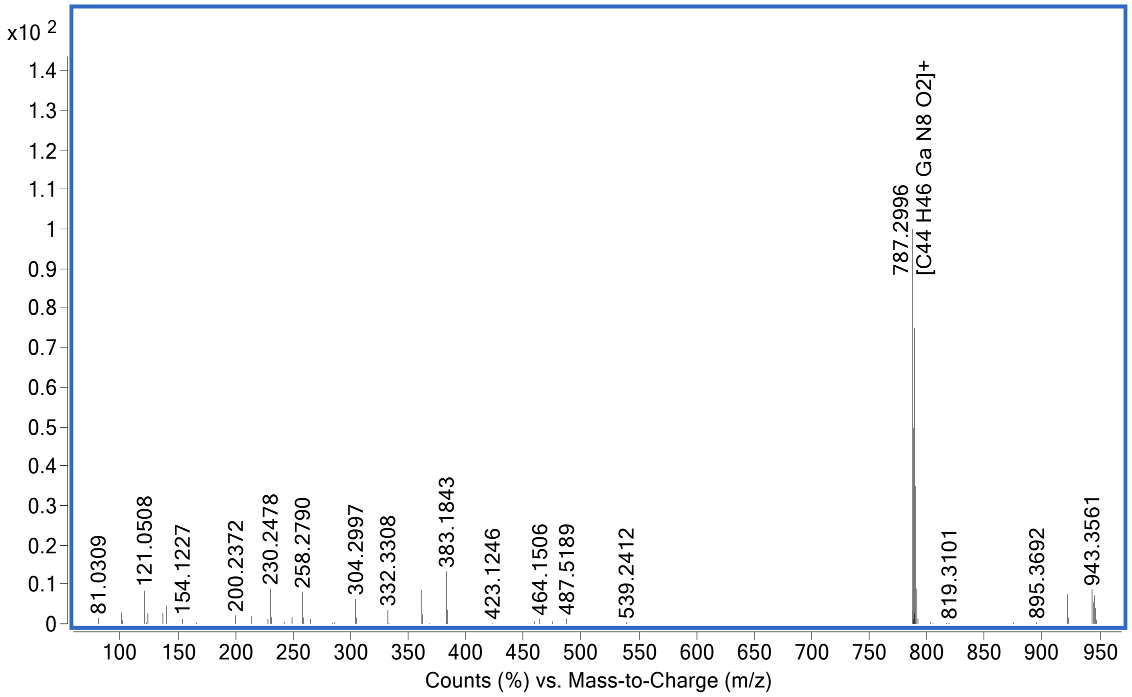

Fig. S22. Mass spectrum of cationic form of each **Ga4** (C_{44} H_{46} Ga N_8 O_2). Molecular structure for the peaks found corresponding to the molecular ion (M+) and of potential fragment ions observed are indicated.

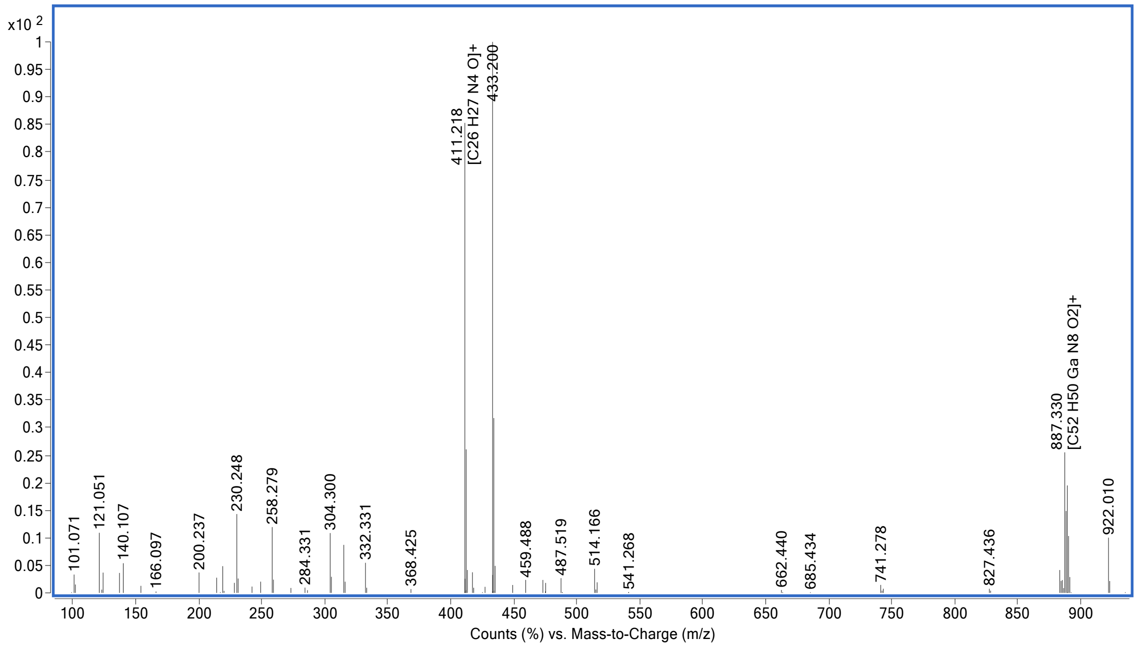

Fig. S23. Mass spectrum of cationic form of each **Ga5** (C_{52} H_{50} Ga N_8 O_2). Molecular structure for the peaks found corresponding to the molecular ion (M+) and of potential fragment ions observed are indicated.

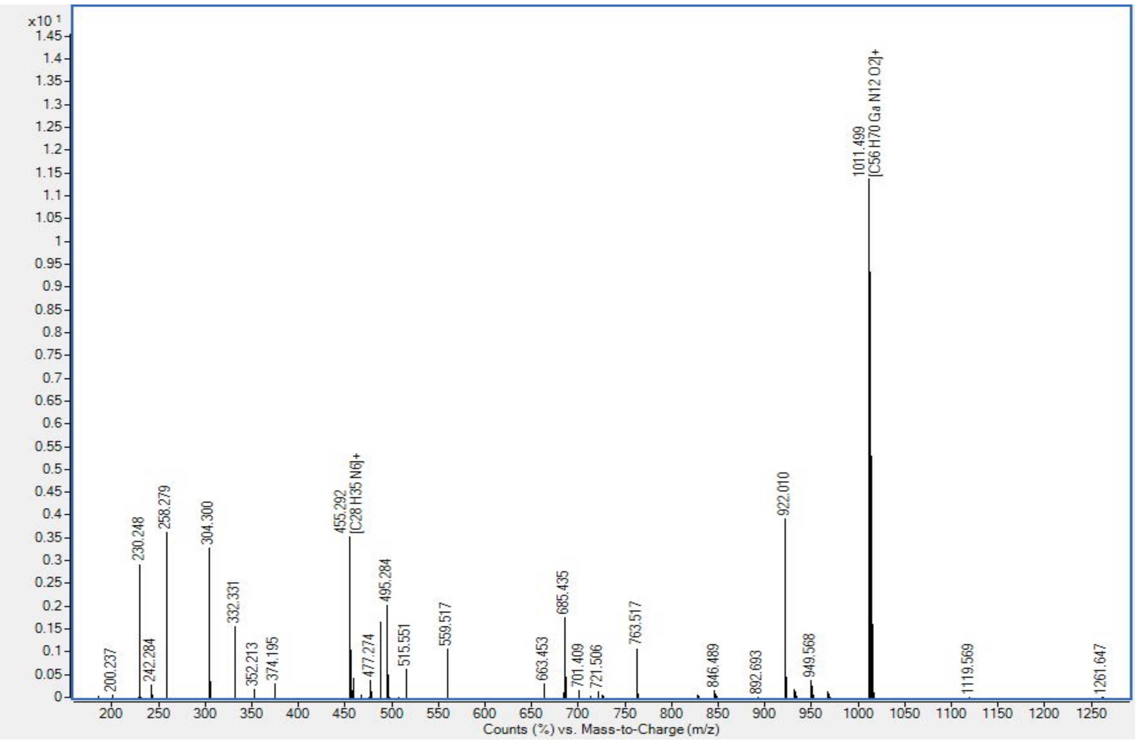

Fig. S24. Mass spectrum of cationic form of each **Ga6** (C_{56} H_{70} Ga N_{12} O_2). Molecular structure for the peaks found corresponding to the molecular ion (M+) and of potential fragment ions observed are indicated.

Table S1. Complete elemental analysis of each gallium compound, depicting calculated and found values.

Gallium complex	Formula	Anal. Calcd	Anal. Found
Ga1	$C_{32}H_{50}Cl_4Ga_2N_8O_2$	C, 44.7; H, 5.9; N, 13.0	C, 44.9; H, 6.2; N, 12.9
Ga2	$C_{44}H_{50}Cl_4Fe_2Ga_2N_8O_2$	C, 47.4; H, 4.5; N, 10.0	C, 47.8; H, 4.7; N, 9.8
Ga3	$C_{52}H_{60}Cl_4Ga_2N_{10}O_2$	C, 54.9; H, 5.3; N, 12.3	C, 55.2; H, 5.5; N, 12.1

Ga 4	$C_{44}H_{46}Cl_4Ga_2N_8O_2$	C, 52.8; H, 4.6; N, 11.2	C, 53.2; H, 5.0; N, 10.9
Ga 5	$C_{52}H_{50}Cl_4Ga_2N_8O_2$	C, 56.8; H, 4.6; N, 10.2	C, 57.0; H, 4.9; N, 10.0
Ga 6	$C_{56}H_{70}Cl_4Ga_2N_{12}O_2$	C, 54.9; H, 5.8; N, 13,7	C, 55.3; H, 6.1; N, 13.4

Table S2. Crystallograsphic data for **Ga5** and **Ga6**.

**Identification code**	**Ga5**	**Ga6**
CCDC number	2498204	2498203
Empirical formula	$Ga_2C_{61}H_{71}Cl_4N_8O_2$	$Ga_2C_{56}H_{70}Cl_4N_{12}O_2$
Formula weight	1229.49	1224.48
Temperature/K	296.15	296.15
Crystal system	monoclinic	triclinic
Space group	$P2_1/c$	*P*-1
a/Å	15.851(3)	12.5047(16)
b/Å	21.114(4)	13.6634(17)
c/Å	18.554(3)	18.204(2)
α/°	90	87.582(4)
β/°	93.940(6)	80.980(4)
γ/°	90	89.556(5)
Volume/Å^3	6195(2)	3069.0(6)
Z	4	2
ρ_{calc}g/cm^3	1.318	1.325
μ/mm^{-1}	1.090	1.101
F(000)	2556.0	1272.0
Crystal size/mm^3	0.28 × 0.16 × 0.04	0.22 × 0.21 × 0.03
Radiation	MoKα (λ = 0.71073)	MoKα (λ = 0.71073)
2Θ range for data collection/°	3.996 to 54.976	4.284 to 52.842
Index ranges	$-20 \le h \le 20$<br>$-24 \le k \le 27$<br>$-22 \le l \le 24$	$-15 \le h \le 15$<br>$-17 \le k \le 17$<br>$-22 \le l \le 21$
Reflections collected	80273	65272
Independent reflections	14145 [R_{int} = 0.1278, R_{sigma} = 0.0791]	12579 [R_{int} = 0.0476, R_{sigma} = 0.0366]
Data/restraints/parameters	14145/78/658	12579/733/761
Goodness-of-fit on F^2	1.031	1.035
Final R indexes [I>=2σ (I)]	R_1 = 0.0676, wR_2 = 0.1797	R_1 = 0.0482, wR_2 = 0.1236

Final R indexes [all data]	R_1 = 0.1353, wR_2 = 0.2280	R_1 = 0.0682, wR_2 = 0.1394
Largest diff. peak/hole / e Å^{-3}	0.63/-0.57	0.59/-0.45

Table S3. Selected bond lengths for **Ga5.**

Number	Atom	Atom	Length/Å
1	Ga1	N1	2.110(3)
2	Ga1	O1	1.889(3)
3	Ga1	O2	1.877(3)
4	Ga1	N3	2.140(4)
5	Ga1	N4	2.084(3)
6	Ga1	N2	2.110(4)
7	Ga2	Cl3	2.139(3)
8	Ga2	Cl2	2.160(3)
9	Ga2	Cl4	2.165(3)
10	Ga2	Cl1	2.168(3)

Table S4. Selected bond angles for **Ga5.**

Number	Atom	Atom	Atom	Angle/°
1	N1	Ga1	N3	86.2(1)
2	O1	Ga1	N1	89.4(1)
3	O1	Ga1	N3	175.0(1)
4	O1	Ga1	N4	88.4(1)
5	O1	Ga1	N2	97.1(1)
6	O2	Ga1	N1	175.8(1)
7	O2	Ga1	O1	94.1(1)
8	O2	Ga1	N3	90.4(1)
9	O2	Ga1	N4	96.2(1)
10	O2	Ga1	N2	86.7(1)
11	N4	Ga1	N1	86.2(1)
12	N4	Ga1	N3	89.2(1)
13	N4	Ga1	N2	173.6(1)
14	N2	Ga1	N1	90.5(1)
15	N2	Ga1	N3	85.1(1)
16	Cl3	Ga2	Cl2	109.9(1)
17	Cl3	Ga2	Cl4	110.2(1)
18	Cl3	Ga2	Cl1	108.8(1)
19	Cl2	Ga2	Cl4	110.6(1)

20	Cl2	Ga2	Cl1	111.1(1)
21	Cl4	Ga2	Cl1	106.2(1)

Table S5. Selected bond lengths **Ga6.**

Number	Atom	Atom	Length/Å
1	Ga1	O1	1.858(2)
2	Ga1	O2	1.857(2)
3	Ga1	N3	2.058(2)
4	Ga1	N5	2.101(2)
5	Ga1	N7	2.136(2)
6	Ga1	N1	2.183(2)
7	Ga2	Cl1	2.126(1)
8	Ga2	Cl4	2.168(1)
9	Ga2	Cl2	2.147(2)
10	Ga2	Cl3	2.151(2)

Table S6. Selected bond angles for **Ga6.**

Number	Atom	Atom	Atom	Angle/°
1	O1	Ga1	N3	89.92(9)
2	O1	Ga1	N5	94.18(9)
3	O1	Ga1	N7	176.25(9)
4	O1	Ga1	N1	85.90(9)
5	O2	Ga1	O1	98.14(9)
6	O2	Ga1	N3	96.25(9)
7	O2	Ga1	N5	92.73(9)
8	O2	Ga1	N7	85.30(9)
9	O2	Ga1	N1	175.90(9)
10	N3	Ga1	N5	169.5(1)
11	N3	Ga1	N7	91.18(9)
12	N3	Ga1	N1	82.95(9)
13	N5	Ga1	N7	84.14(9)
14	N5	Ga1	N1	87.70(9)
15	N7	Ga1	N1	90.68(9)
16	Cl1	Ga2	Cl4	109.27(7)
17	Cl1	Ga2	Cl2	109.60(8)
18	Cl1	Ga2	Cl3	108.44(8)
19	Cl2	Ga2	Cl4	106.13(7)

20	Cl2	Ga2	Cl3	110.62(9)
21	Cl3	Ga2	Cl4	112.74(7)

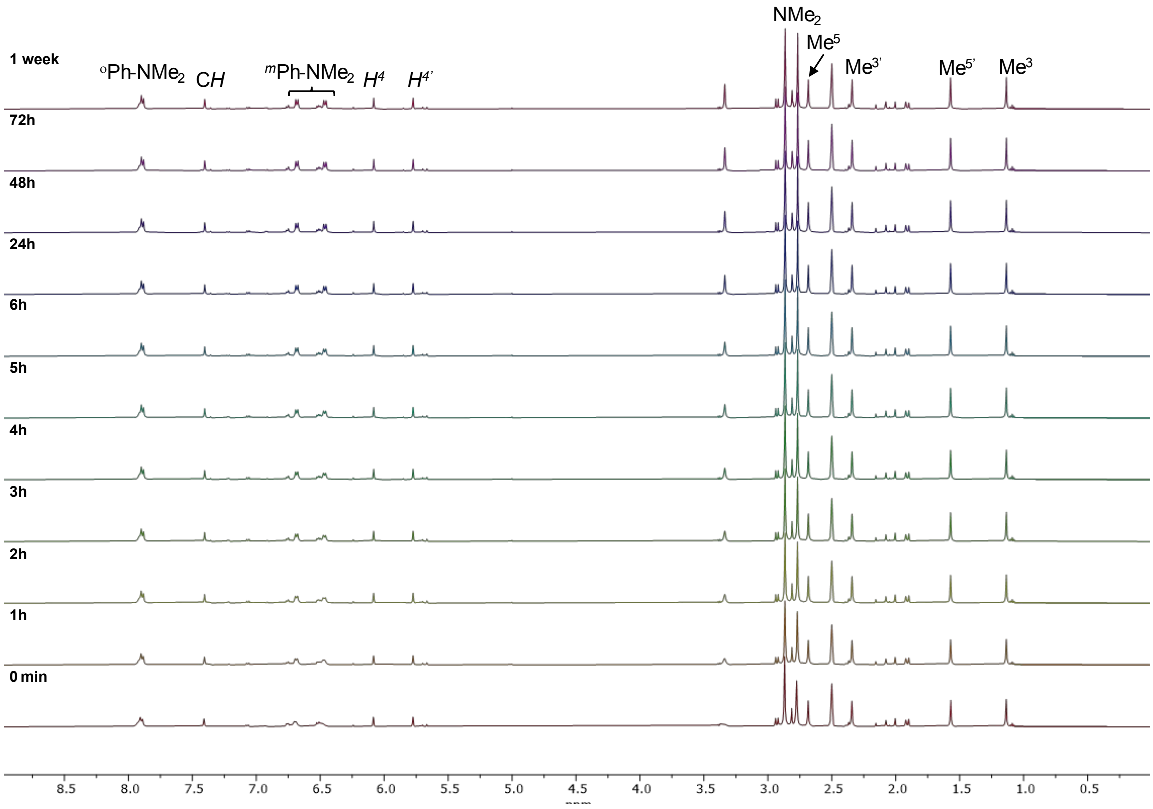

Fig. S25. Stability of **Ga6** in DMSO-d_6 over 1 week. ^{1}H NMR spectra at different times.

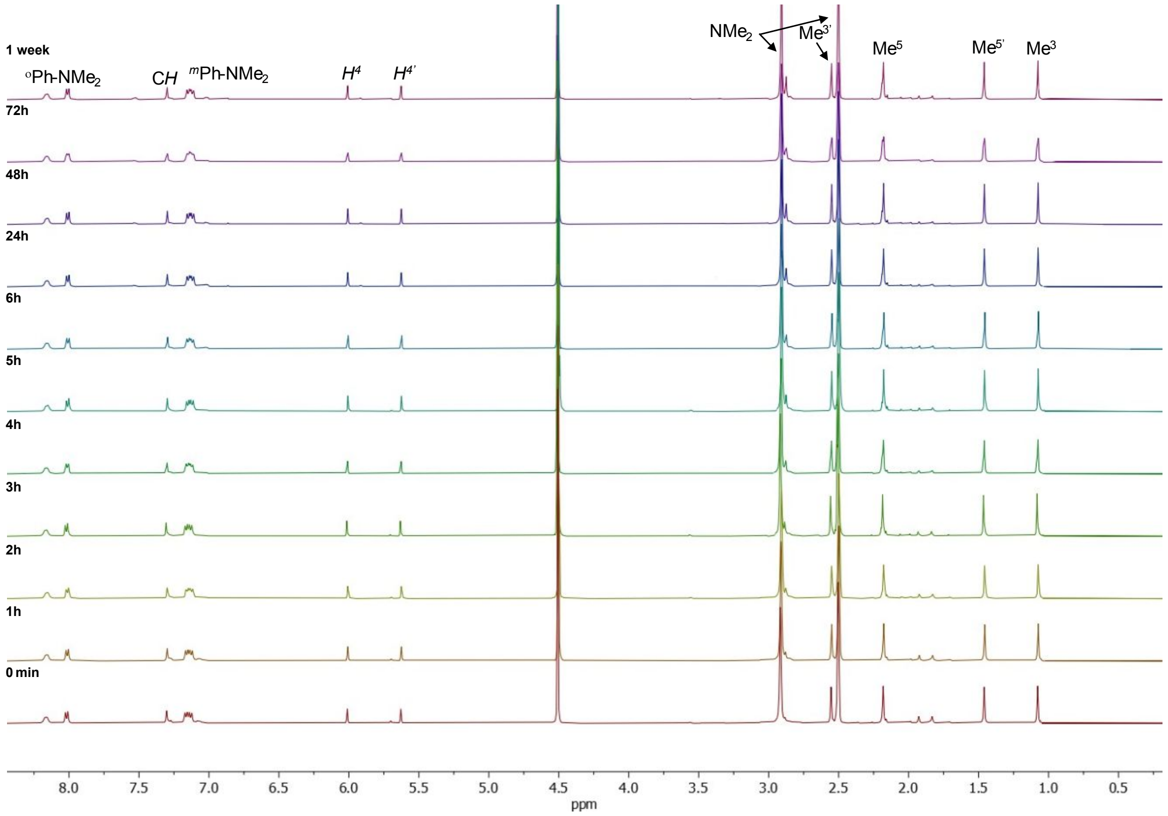

Fig. S26. Stability of **Ga6** in DMSO-d_6:D_2O (1:1) over 1 week. ^{1}H NMR spectra at different times.

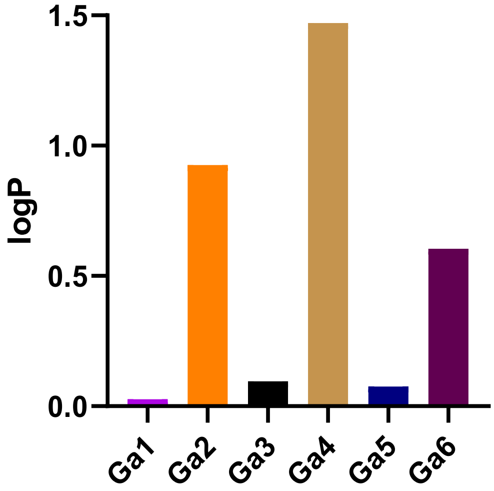

Fig. S27. Graph of the calculated lipophilicity of **Ga1-Ga6** complexes by logP.

Table S7. logP data for each complex.

Complex	logP
Ga1	0.027
Ga2	0.925
Ga3	0.095
Ga4	1.471
Ga5	0.076
Ga6	0.604

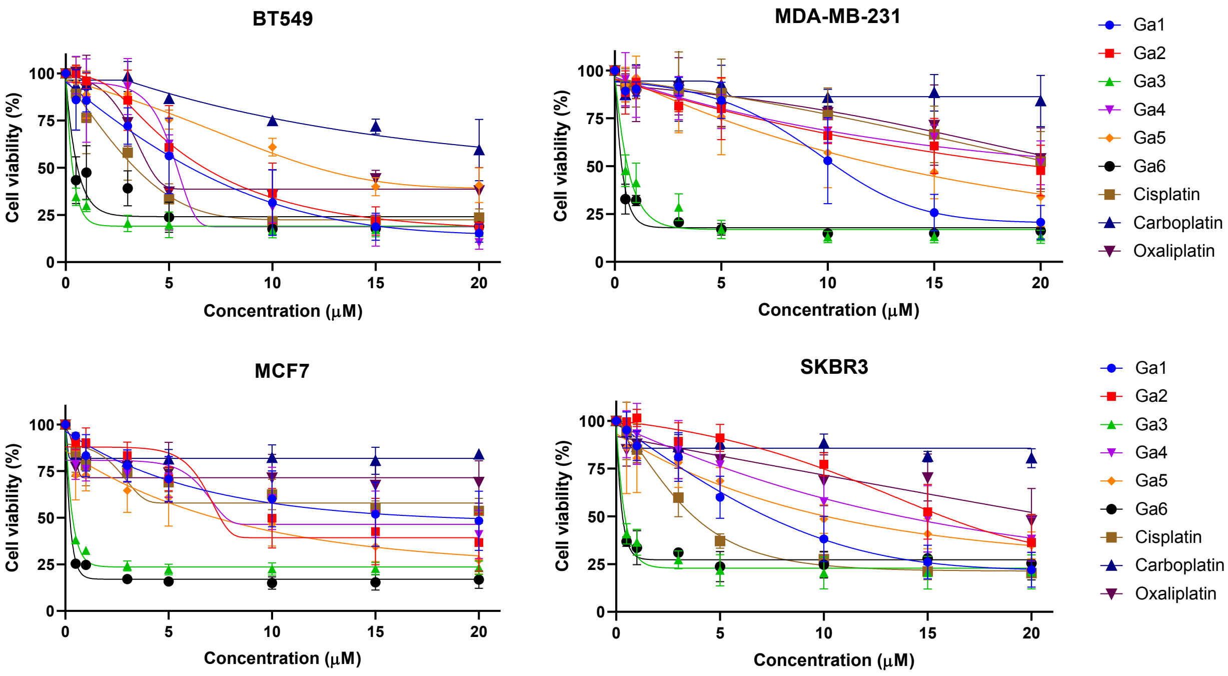

Fig. S28. Dose-response curves of **Ga1-Ga6**, cisplatin, carboplatin and oxaliplatin in four breast cancer cell lines.

A)

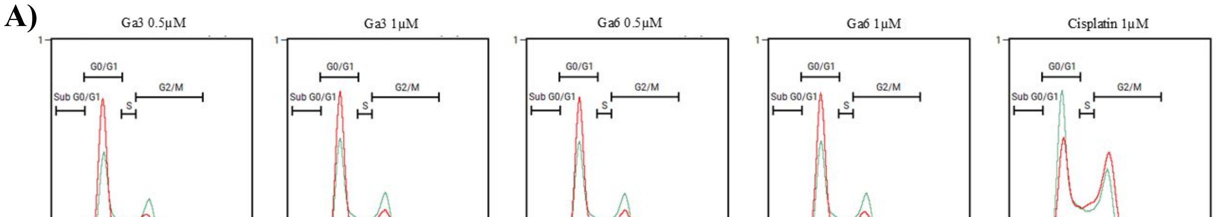

Fig. S29. Representative example of diagrams of cell populations in each phase of the cell cycle after 24h of treatment of MDA-MB-231 (A) and MCF7 (B) with **Ga3** and **Ga6**. Red histogram is for the treatment and green histogram is for the control. Histograms are normalized to the area. PE-A: propidium iodide.

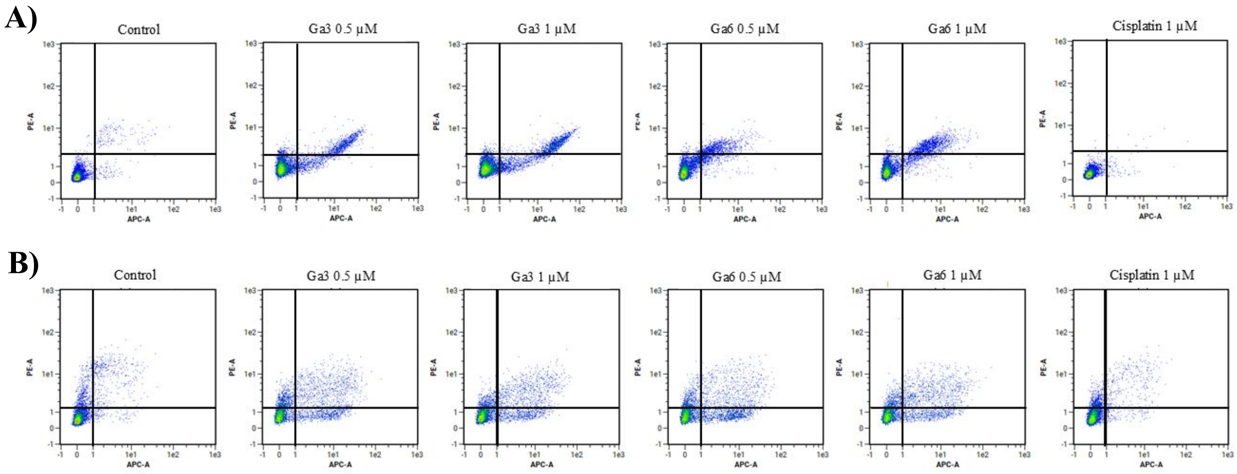

Fig. S30. Representative example of dot plot graphs from the apoptosis analysis of MDA-MB-231 (A) and MCF7 (B) after 72h of treatment with **Ga3** and **Ga6**. PE-A: propidium iodide. APC-A: annexin V.

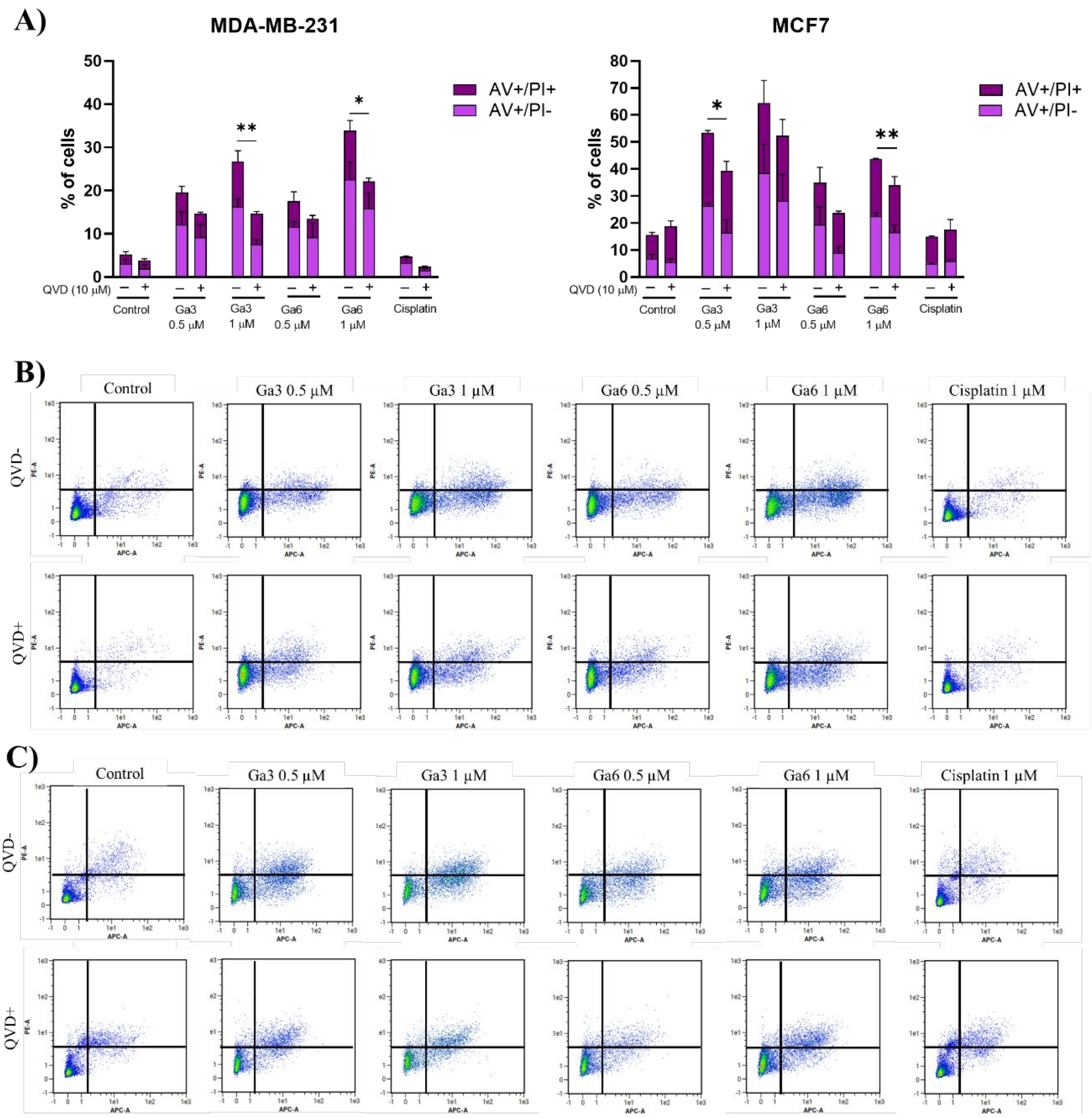

Fig. S31. Apoptosis assay was performed using pan-caspase inhibitor Q-VD-OPh before treatment with gallium compounds. A) Quantification of early (AV+/PI-) and late (AV+/PI+) apoptotic cells. To determine significant statistical differences, a Student's t-test between AV+ cells of each condition was used. The values for the statistical analyses are * $p \leq 0.05$; ** $p \leq 0.01$. Representative example of dot plot graphs for MDA-MB-231 (B) and MCF7 (C). PE-A: propidium iodide. APC-A: annexin V.

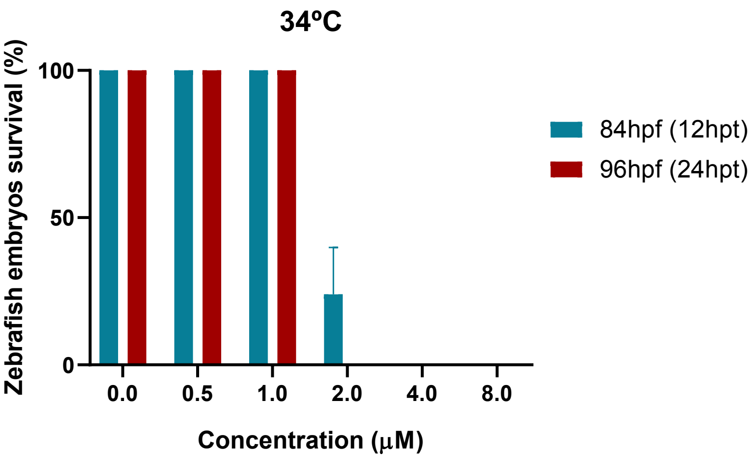

Fig. S32. Dose-dependent effects of **Ga6** on zebrafish embryo survival at 34°C. Data are represented as mean ± SEM from three independent experiments.

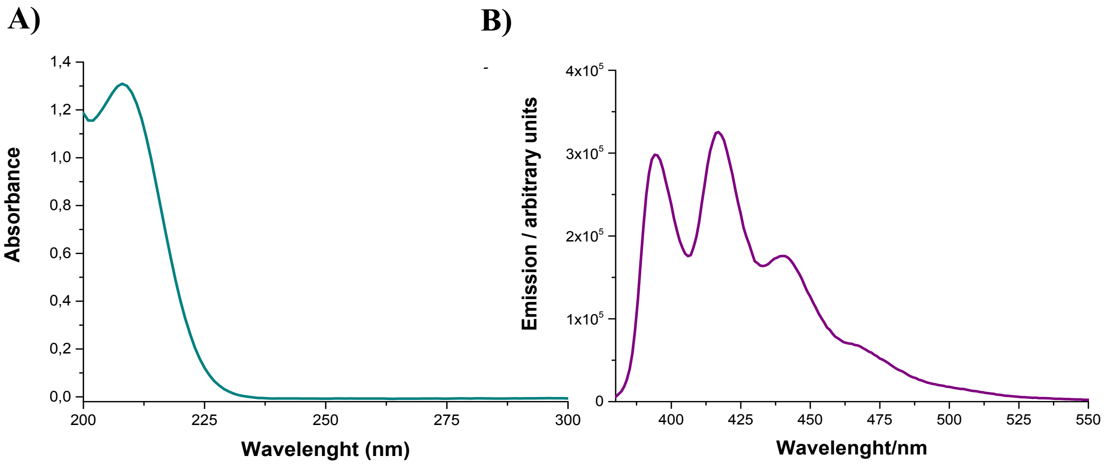

Fig. S33. Absorption (A) and emission (B) spectra of **Ga5**.
